# GCNPath: introspecting drug response prediction with pathway-guided graph convolution networks

**DOI:** 10.1038/s42003-026-09957-5

**Published:** 2026-04-01

**Authors:** Hyeon Jun Yoon, Minho Lee

**Affiliations:** Department of Life Science, Dongguk University, Seoul, Republic of Korea

**Keywords:** Machine learning, Virtual screening

## Abstract

Drug response prediction (DRP), accounting for the diverse biological characteristics of cancer types that affect sensitivity or resistance to treatment, is crucial for anticancer drug selection and discovery. Although numerous deep learning models for DRP have been developed, it has not been investigated whether these models can maintain reliable predictive power when applied to omics datasets not used for training, which is crucial for real-world applications. Moreover, they have not been rigorously examined through systematic benchmark tests to investigate the relationships between model architecture and performance. We present a new model, GCNPath, that exploits both graph convolution network (GCN) architectures and pathway-based feature reduction of gene expression data, which have previously been shown to improve DRP model performance. In comprehensive benchmark tests utilizing multiple cell omics platforms, GCNPath shows robust and competitive performances compared with state-of-the-art models including prediction of unseen drugs and the ability to overcome batch effects across various RNA datasets. This study demonstrates the validity of the pathway-level GCN model in DRP and suggests directions for developing DRP models with improved adaptability to diverse and heterogeneous datasets and enhanced usability for practical applications.

## Abstract (web summary)

We developed a graph convolutional drug response prediction model that captures inter-pathway relationships, enabling large-scale drug screening and realistic clinical response simulation.

## Introduction

Genomic and transcriptomic variability in cancers can result in differing responses to anticancer drugs, even among tumors of the same tissue type. Molecular features such as mutations and gene expression patterns influence therapy sensitivity and resistance, making, making a universal treatment approach less effective^1^. Therefore, considering the molecular landscapes of tumors is crucial when identifying candidate anticancer drugs. To address this, large-scale public databases like the Genomics of Drug Sensitivity in Cancer (GDSC)^2^ database and the Cancer Cell Line Encyclopedia (CCLE)^3^, have been established. These databases link cancer multi-omics profiles with drug efficacy at the cell line level. They contain data from exposing numerous cancer cell lines to hundreds of anticancer drugs at various doses. Drug efficacy is evaluated using metrics like half-maximal inhibitory concentration (IC_50_) and area under the drug response curve (AUC). Despite the availability of these resources, exploring every possible drug-cell line combination remains costly and labor-intensive, particularly for cell lines with unique characteristics not previously covered in drug response screenings. This challenge has driven the development of computational methods for drug response prediction (DRP), with machine learning increasingly employed for this purpose^4,5^.

Among the various machine learning methods, deep learning models stand out due to their superior prediction accuracy and interpretability^6,7^ (Supplementary Table 1). For example, tCNNS^8^ processes drug structures as atomic one-hot vectors and detects sequential atoms properties between with one-dimensional convolution neural network (CNN) layers. Recently, graph convolution networks (GCNs) have been widely used to process data with complex relationships between entities such as social networks, chemical networks of atom graphs, and protein‒protein interactions (PPIs) in cells^9^. In DRP, GCNs can capture intricate and diverse interactions between genes or atoms, and have been applied in multiple tools, such as GraphDRP^10^, TGSA^11^, and DRPreter^12^. Additionally, models like PaccMann^13^, HiDRA^14^ and DRPreter^12^ use attention networks to focus on critical genes and pathways that influence drug responses. These deep learning models have outperformed compared with traditional machine learning or other state-of-the-art models.

Despite the emergence of numerous deep learning models, there remain two major shortcomings or issues to consider in previous DRP studies. First, these various models have not been subjected to comprehensive and extensive benchmark testing representing diverse situations, which is necessary for assessing the versatilities of DRP model beyond training data. To ensure the practical usability of DRP models, it is important to carry out benchmark tests with combinations of cell lines and drugs, both of which are not included in the training data (strict-blind test)^5,7^. Notably, while some models^15,16^ undergone these intricate tests, comparisons are often limited to only a few DRP models. Furthermore, benchmark tests generally rely on a narrow set of drug response data, typically utilizing only one of the two available GDSC datasets or another similarly limited dataset for both training and testing. Most importantly, there is a lack of rigorous external validation with real-world datasets, such as ChEMBL^17^, which encompass a much broader chemical space.

A second major drawback is that the optimal preprocessing method to resolve technical biases inherent in cell-line expression data remains unclear. While transcriptome data is the most informative for drug response prediction^18,19^, it often contains systematic variability, such as batch effects during RNA expression measurement. To build models with generalizability beyond the training dataset, it is crucial to minimize sensitivity to such biases^7^. Notably, some models^20,21^ use compressed transcriptome data represented as pathway activation scores, which can improve performance and address batch effects across cell-line datasets. However, batch correction in deep learning-based DRP models has rarely been tested, and it remains to be explored whether pathway-based methods integrated with the advanced architectures such as GCNs can further enhance prediction powers.

In this study, we introduce GCNPath, a novel model that combines GCN architecture with pathway-based feature reduction of transcriptomic data. GCNPath represents cell line data using transcriptomic PPI and gene regulatory network (GRN) graphs compressed at the pathway level, along with a correlation graph of pathway activity scores. For drug data, our model uses graph representations to capture the intricate atomic and bond topologies, rather than relying on simplified 1-dimensional compound fingerprints. We validate competent performances of GCNPath through extensive benchmark tests, including strict-blind tests using GDSC1 and GDSC2 datasets, and external validation with ChEMBL. Additionally, we presented a benchmark test with TCGA dataset and a case study on small cell lung cancer (SCLC) to demonstrate the clinical utility of our model. Notably, our study introduces a novel approach that exploits the multifaceted crosstalk between pathways through GCNs in DRP model architectures.

## Results

### The overall workflow of GCNPath

**Fig. 1:**
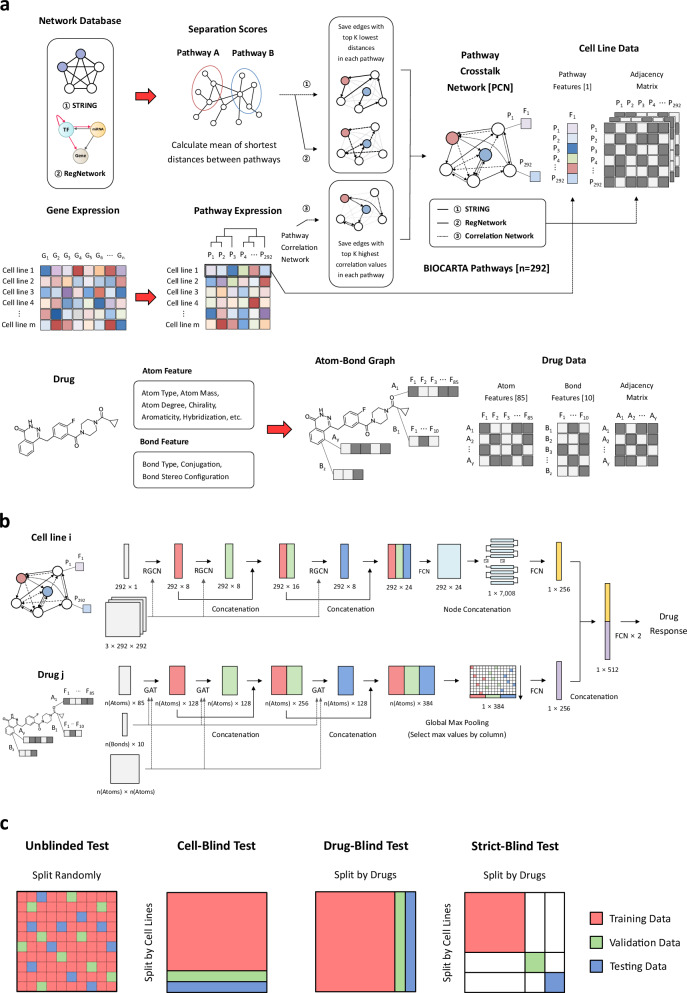
An overview of GCNPath. **a** Data Processing: GCNPath uses three pathway crosstalk network (PCN) graphs to represent cell lines: (1) Protein-protein interaction (PPI) networks from Search Tool for Retrieval of Interacting Genes/Proteins (STRING)^55^, (2) Gene regulatory network (GRN) from Regulatory Network Repository (RegNetwork)^56^, and (3) correlation networks from pathway activity scores. Networks (1) and (2) are compressed from gene/protein into pathway level using pairwise separation scores^23^ or gene overlap ratios, connecting the top 5 pathways with lowest separation scores or highest overlap. PCN graphs incorporate pathway scores derived from gene expression data via gene set variation analysis (GSVA)^22^, based on 292 BIOCARTA pathways from MSigDB^54^. Network (3) retains the top 5 pathways with the highest Pearson’s correlations calculated on GSVA scores. Note that the selection of neighbor pathways introduces directionality into PCN graphs: a pathway may connect to its nearest neighbors, but if those neighbors have stronger functional associations with other pathways, the reverse connection may not occur. Our study focused on horizontal rather than hierarchical relationships, and PCN can be constructed from any pathway databases such as BIOCARTA and KEGG. Drug information is converted into atom-pair graphs with 85 atoms and 10 bond features, including atom‒bond types, chirality, and hybridization. **b** Model Architecture: GCNPath predicts ln(IC_50_) values using cell and drug inputs. The cell module uses a relational GCN (RGCN)^24^ to process multiple PCN graphs, while the drug module uses a graph attention network (GAT)^69^ to extract atom graph features. Both modules adopt DenseGCN architectures^70^, where outputs of previous layers are concatenated to preserve information. For example, outputs from the first two layers (red and green) are concatenated and fed into the third layer (blue). Final embeddings from the cell and drug modules are combined and processed with FCNs to predict drug responses. **c** Testing types: The performances of DRP models were evaluated across unblinded and blind tests.

We developed GCNPath, a pathway-informed graph neural network model for cell-line based drug response prediction, designed to be robust and generalizable across diverse preclinical and clinical datasets (Fig. 1a). The framework begins by transforming RNA-seq transcriptomic profiles from cell lines into pathway activity scores using Gene Set Variation Analysis (GSVA)^22^, which reduces batch effects while preserving biologically relevant signals. Unlike conventional batch correction tools such as ComBat, GSVA operates in an unsupervised manner, requiring no sample labels (e.g., tissue type, disease status, or batch identity) or alignment to a reference cohort.

These pathway-level features are then structured into pathway crosstalk networks (PCN), computed using separation scores^23^ derived from two biological networks and pairwise pathway correlations. The construction of PCN graphs is motivated by two premises: (1) pathways whose genes or proteins are spatially or functionally proximate in biological networks, and (2) pathways that display similar activity score patterns are likely to exhibit functional similarity or co-expression. This representation captures the complex functional interplay between pathways— a rarely explored aspect in previous studies—as illustrated in Supplementary Note 1 (Supplementary Figs. 1, 2**;** Supplementary Table 2; Supplementary Data 1, 2). GCNPath leverages relational GCN (RGCN)^24^ and graph attention networks (GAT) to effectively model the inherent graph structures of both cell lines and drugs, enabling accurate prediction of drug sensitivity (Fig. 1b).

To assess the practical utility of GCNPath, we conducted extensive benchmarking experiments. Although GCNPath does not achieve overwhelming performance gains over existing state-of-the-art models, it consistently delivers the most balanced improvements across challenging prediction scenarios involving previously unseen cell lines and/or drug compounds (Fig. 1c). Notably, GSVA-derived features enhance the model’s robustness not only to batch effects but also across different expression platforms, including TPM, microarray, and proteomics. GCNPath outperformed other tools on ChEMBL dataset simulating large-scale chemical screening, and effectively identified biologically meaningful determinants of drug sensitivity in colorectal, breast, and small-cell lung cancer (SCLC) at the cell-line level. Finally, its applicability to real-world settings was demonstrated through external validations using the TCGA clinical dataset and an SCLC case study, where it successfully predicted drug efficacy and distinguished cancer from normal samples. These results support GCNPath as a practical and scalable framework for high-throughput drug screening and translational pharmacogenomics.

### Leveraging graph characteristics in cell and drug modules of GCNPath enhances performance

Pathway-based feature reduction effectively captures key transcriptomic characteristics of cell lines for the DRP problem, but pathway interplays have rarely been integrated to the feature reduction due to methodological limitations in identifying these interactions. This section presents a proof-of-concept for PCN graphs, evaluating whether pathway-guided GCN in the cell module optimizes DRP. Additionally, we investigated GCN adoption in the drug module, comparing it with FCNs that process 1-dimensional chemical fingerprints. Before graph-based representations, drugs were typically characterized using substructure-quantifying methods like Morgan fingerprints^25^ or sequential encodings like SMILESVec^26^. These features, processed through FCNs, partially incorporated graphical properties. Here, we test whether GCN architectures, designed to capture atom-bond graph structures, outperform 1-dimensional features.

**Fig. 2:**
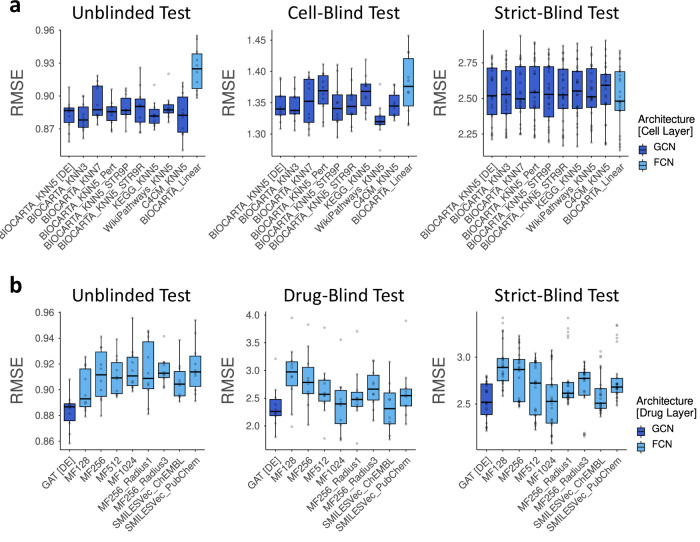
Ablation tests in cell and drug modules. During the tuning process, 25-fold and tenfold outer cross-validations were performed on the strict-blind and remaining test sets, respectively, using GDSC2 as the target label dataset. This resulted in 25 and 10 RMSE values per box (*n* = 10 or 25 for each box). For both (**a**) the cell and **b** drug modules, parameters were grouped into two categories depending on whether GCN or fully connected networks (FCNs) were used. These categories are highlighted in dark blue and light blue in each panel, respectively. The default architecture is marked as “[DE]” in x-axis. In all boxplots, each box spans the IQR, with whiskers extending 1.5×IQR. The source data are provided in Supplementary Data 3. KNN: K-nearest neighbors per pathway in each PCN graph (K = 3, 5, and 7). Pert: PCN graphs with perturbed edges, preserving node degree to maintain local topology. Noise: Gaussian noise (N(0, 1)) added to pathway activity scores. KEGG, WikiPathways, C4CM: PCN graphs constructed from KEGG, WikiPathways and cancer-specific gene modules curated from MSigDB (v2023.1). Linear: FCN in the cell module without PCN graph structure. GAT: graph attention network (GAT) in drug module processing atom-bond graph; MF: FCN in the drug module processing the Morgan fingerprint (128, 256, 512 or 1024 bits with 1, 2 or 3 radius)^25^; SMILESVec: FCN in the drug module processing the SMILESVec representation (100 bits, word-level embeddings trained with ChEMBL23 and PubChem)^26^.

Ablation tests used GDSC2, chosen for computational efficiency during training and testing compared to GDSC1 and the combined dataset of both GDSC1 and GDSC2 (GDSC1 + 2). Model performances were evaluated using RMSE, measuring errors between predicted and actual natural log-normalized IC_50_ (ln(IC_50_)) values, where lower RMSE indicates better performance. The results showed graph-based architectures achieved significantly lower RMSEs than FCNs in the cell module, highlighting the value of incorporating PCN graphs (Fig. 2a; Supplementary Data 3). We found that perturbations of PCN graphs led to decreased performance in cell-blind tests, suggesting that the topological structure of PCN graphs encodes biologically meaningful knowledge and insights. Other factors—such as the number of neighbors, the choice of pathway database, and the type of PCN construction— also influenced predictive powers, either marginally or not at all. In the drug module, GAT outperformed FCN for processing Morgan fingerprints and SMILESVec embeddings, demonstrating that graph structures better capture drug characteristics for DRP (Fig. 2b; Supplementary Data 3). In summary, GCNPath consistently achieves outstanding performances by exploiting graph-based representations in both cell and drug modules.

### GCNPath performance is comparable or superior to that of other DRP models in various test situations

After constructing PCN graphs, we evaluated whether integrating diverse pathway interactions enhances DRP performance, benchmarking against state-of-the-art DRP models. As stated in the “Introduction”, we systematically assessed multiple deep learning-based DRP models using various drug response datasets and test scenarios, focusing on architectures suitable for ln(IC_50_) regression. Specifically, we investigated whether incorporating GCN and/or attention mechanisms for drug–gene or drug–pathway interactions improves model performances^7^. For consistent and practical benchmarking, we focused on models that provide publicly available source code, require only standard omics and drug structure inputs, and do not rely on extensive hyperparameter tuning. To validate the performance advantage of deep learning approaches, we included two traditional non-deep learning methods—Random Forest^27^ and BMTMKL^28^—as baseline models.

Selected models were trained and tested on GDSC1, GDSC2, and GDSC1 + 2. GDSC was chosen for its comprehensive drug response data, enabling robust training and evaluation. Using both versions maximized available data. Four test scenarios addressed distinct aspects of predictive performance: (1) Unblinded tests assessed baseline predictability using known cell lines and drugs. (2) Cell-blind tests simulated precision oncology, predicting drugs for new cell lines. (3) Drug-blind tests focused on novel drug responses in established cell lines. (4) Strict-blind tests combined these challenges to evaluate robustness and generalizability. The cell lines and drugs for training, validation, and testing in blind tests were randomly selected.

Before presenting results, we clarified naming conventions for DRP model variants. Especially, to ensure a fair evaluation of DRP models, we considered discrepancies in the training and testing datasets caused by differences in the cell-line gene expression data sources, despite all models using IC_50_ values from GDSC as target label datasets (Supplementary Table 3). While GCNPath used TPM data from SANGER Cell Model Passports, other models like DRPreter, TGDRP, TGSA, and PaccMann used CCLE or GDSC data, limiting available cell lines. To address this, we separately trained models on both original training data utilized by the model developers and TPM from SANGER Cell Model Passports, designating results from the latter as “model_name_SG” (Supplementary Fig. 3a). Additionally, DRPreter_SA denotes a DRPreter variant incorporating the GCN modules introduced in TGSA, leveraging transcriptomic and drug structural similarities (Supplementary Fig. 3b).

**Fig. 3:**
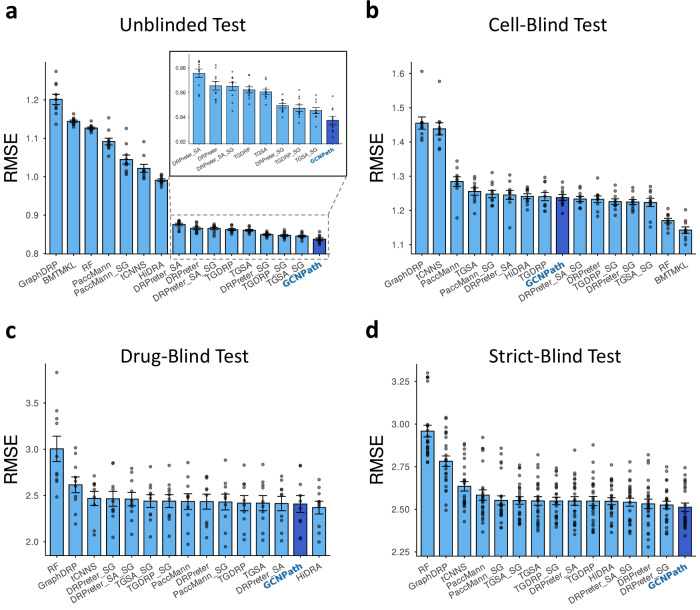
GCNPath exhibits superior or comparable performance to existing DRP models in tests under different blinding scenarios. All models were trained and evaluated using GDSC1 + 2 as a target label dataset. They were tested with tenfold outer cross-validation in (**a**) unblinded, **b** cell-blind, and **c** drug-blind tests, and 25-fold outer cross-validation in (**d**) strict-blind tests, resulting in (**a–c**) 10 RMSEs and **d** 25 RMSEs per model (*n* = 10 or 25 for each bar). GCNPath performance is highlighted in dark blue across all panels. Outliers from tCNNS were excluded (Supplementary Tables 4–6). RMSEs are reported as mean ± standard errors, represented by error bars. The source data are provided in Supplementary Data 4. RF: Random Forest. SG: model trained with cell line data from SANGER Cell Model Passports as cell-line input data. DRPreter_SA: DRPreter with a similarity augmentation module.

The model performances assessed using RMSEs, Pearson correlation coefficients (PCCs), and Spearman correlation coefficients (SCCs), demonstrated that low RMSE and high PCC/SCC values are crucial for effective DRP models. In benchmarks with GDSC datasets, GCNPath consistently achieved the best or comparable results (lowest RMSEs) across unblinded (Fig. 3a), cell-blind (Fig. 3b), drug-blind (Fig. 3c), and strict-blind tests (Fig. 3d), as shown in Supplementary Figs. 4–7 (Supplementary Data 4–8). Like GCNPath, TGDRP, TGSA, and DRPreter strongly outperformed non-GCN models in unblinded tests, but their advantages diminished in drug-blind and strict-blind scenarios. In contrast, GraphDRP, which applies GCNs only to drug data, exhibited higher RMSEs than other GCN-based models. Among non-GCN models, HiDRA outperformed tCNNS across all scenarios, likely due to its attention mechanisms, which enhance the capture of nuanced drug‒gene and drug‒pathway interactions. PaccMann, which is limited to drug–gene attention, showed better performance than tCNNS in cell-blind tests but performed similarly in other scenarios. Random Forest and BMTMKL, both non-deep learning methods, achieved notable results in cell-blind tests but exhibited limited generalizability in drug-blind and strict-blind scenarios. Performance patterns remained consistent regardless of whether RMSE, PCC, or SCC were used as metrics (Supplementary Figs. 8–13; Supplementary Tables 4–6; Supplementary Data 9–14). These results highlight the effectiveness of combining GCNs with pathway-informed feature extraction for DRP.

We calculated RMSE values by cell tissue types and drug target pathways (Supplementary Figs. 14, 15; Supplementary Data 15, 16). GCNPath showed performance trends consistent with its overall superiority, as seen in the bar plots. While performances were broadly similar across models, variations emerged in specific tissue types and pathways, as visualized in heatmaps and scatter plots comparing GCNPath with the top two models. Notably, GCNPath outperformed others in tissues such as blood cancers and thyroid (e.g., BLL in unblinded; CHDM and CLL in cell-blind; THCA in strict-blind), and in pathways related to hormone signaling (unblinded) and chromatin histone acetylation (drug-/strict-blind). Additionally, GCNPath demonstrated somewhat variable yet consistently balanced performance across most tissue types and target pathways, indicating its robustness and generalizability across diverse input categories (Supplementary Figs. 16, 17; Supplementary Data 17, 18). These patterns held across training datasets—GDSC1, GDSC2, and GDSC1 + 2 (Supplementary Figs. 18, 19; Supplementary Data 19, 20).

To this point, we examined GCNPath and other DRP models that were trained from scratch. To investigate whether the poorer performance of other tools compared with those of GCNPath was due to our independent training, we examined the performance of the pretrained models (i.e., the models as trained by their own developers). To achieve this, we evaluated the performance of both the DRP models trained with GDSC1 + 2 as a target label dataset in various test situations, and the pretrained ones (where available) for each cell line and drug, using RMSE metrics (RMSE_C_ and RMSE_D_, respectively). We found that the RMSE_C_ and RMSE_D_ values of the pretrained models show patterns similar to those of the trained models, especially in the drug-blind and strict-blind tests (Supplementary Fig. 20; Supplementary Data 21). This means that the pretrained models, like the models trained in this study, are weak at making predictions for unseen cells and/or drugs. TGDRP_Pre and TGSA_Pre, which incorporate self-supervised learning from ZINC15 and ChEMBL, showed no notable improvements over their counterparts without this step (Supplementary Figs. 21, 22; Supplementary Table 7**;** Supplementary Data 22, 23). In conclusion, the challenges in predicting unseen cells and drugs stem from the limitations in model architectures, rather than training processes, confirming the validity of the benchmark findings.

### The impact of inherent characteristics of cell lines and drugs on DRP performances were recapitulated

Training and testing DRP models with GDSC datasets showed that drug-blind and strict-blind tests are more difficult than unblinded and cell-blind tests. This suggests that deep learning models, despite diversity and complexity in their architectures, may still struggle to accurately predict unseen drug responses. To identify factors influencing DRP performance, we referenced Shen et al. ^6^ that revealed the consistent predictive abilities of DRP models, regardless of the model architectures. Shen et al. ^6^ also suggested that the ability to predict the effects of individual drugs depends on the intrinsic characteristics of the drugs; in particular, the variation in their observed responses exhibits an favorable predictability.

To explore these findings and other potential factors, we evaluated DRP model performances per cell line and drug using prediction performances with SCC metrics for each cell and drug (SCC_C_ and SCC_D_, respectively) across various test scenarios. We chose SCC, the same performance metric used in Shen et al. Our analysis revealed strong correlations of SCC_C_ and SCC_D_ between models across test types (Supplementary Figs. 23, 24; Supplementary Data 24, 25), supporting the findings of Shen et al. ^6^ Given the consistency of performance across models, we used prediction results from GCNPath for further analyses (Supplementary Fig. 25; Supplementary Data 26). We also confirmed a positive correlation between SCC_D_ and the standard deviation of actual ln(IC_50_) values for individual drugs, as well as a weaker or similar correlation between SCC_C_ and the standard deviation of ln(IC_50_). This relationship, which remained modest but consistent even under blind test conditions. Shen also emphasized two other factors: the bimodality of ln(IC_50_), which reflects selective anticancer activity, and density coverage, which represents the similarity between the ln(IC_50_) distribution for each drug or cell and that of the entire GDSC dataset. Although these factors showed notable influence in unblinded settings, their impact was diminished or inconsistent in blind test conditions.

We investigated additional factors not examined by Shen (Supplementary Fig. 25; Supplementary Data 26). The mean ln(IC_50_) values—reflecting overall sensitivity or drug toxicity—showed limited and inconsistent relevance across different test types. The number of available ln(IC_50_) measurements per cell or drug was also not a strong predictor of performance at the individual level; in many cases, performance varied significantly even when data were abundant. However, when grouped by tissue type (for cell lines) or by target pathway (for drugs), a clear trend emerged: groups with more data tended to show higher predictive performance. Lastly, we found that the intrinsic variability of these values, capturing the uncertainty in ln(IC_50_) measurements, had a minor yet measurable impact on the model’s ability to predict unseen drug responses (Supplementary Fig. 26; Supplementary Data 27).

We also assessed the impact of drug response datasets for training on overall model performance. GDSC1 + 2 contains the most drugs (432), and response values compared to GDSC1 (321) and GDSC2 (236). However, our benchmarks showed that combining GDSC1 and GDSC2 did not prominently enhance predictive performance in drug-blind or strict-blind tests (Supplementary Figs. 8–10, 18b, 19b; Supplementary Tables 4–6; Supplementary Data 9–11, 19, 20). Interestingly, training with GDSC1 led to lower PCCs and SCCs than training with GDSC2 or GDSC1 + 2, possibly due to differences in data quality. To estimate ln(IC_50_) values, cell viability was measured after treatment with multiple concentrations. GDSC2 employs a broader range between the maximum and minimum screening concentrations than GDSC1 (1000- or 1024-fold ratio vs. 256-fold ratio), allowing for more accurate ln(IC_50_) calculations. Thus, the characteristics of drug response datasets could influence model performance.

### GCNPath performs well in drug-blind scenarios and handling heterogeneous RNA platforms

To evaluate the usefulness of the trained models for predicting novel drug responses, we predicted IC_50_ values for 237,580 drug–cell line combinations mined from ChEMBL^17^. While external datasets in most DRP benchmarks are usually sourced from drug response databases like CCLE, CTRP, or NCI-60, we chose ChEMBL to better reflect real-world scenarios. Unlike high-throughput screening datasets, ChEMBL consolidates drug response data from various labs, offering a broader and more heterogeneous representation of experimental conditions. We excluded drugs present in both ChEMBL and GDSC1 + 2 to simulate a drug-blind test scenario.

**Fig. 4:**
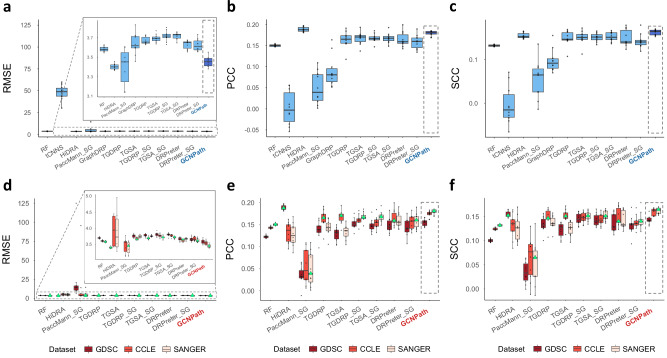
Performance of selected models in external tests with ChEMBL dataset. All DRP models were trained ten times using the entire GDSC1 + 2 as a target label dataset, with predictions performed on ChEMBL as a target label dataset (*n* = 10 for each box). The input data included either (**a–c**) the omics datasets used during model training or **d–f** modified versions, where expression profiles were replaced with those from external cell line datasets—applied only to models that take gene expression data as input. Models used one of three gene expression datasets—GDSC, CCLE, or SANGER Cell Model Passports—as input, denoted by green triangles (Supplementary Table 3). GCNPath performances are highlighted in dark blue within dotted square boxes in panels (**a–c**). In (**a–c**), outlier predictions from tCNNS were excluded (Supplementary Table 8), while in (**d–f**), tCNNS and GraphDRP were excluded because they do not utilize gene expression data. To improve visibility, RMSE scores below 5 were zoomed in for panels (**a**) and (**d**). Boxplots display the interquartile range (IQR), with whiskers extending 1.5×IQR. Source data are provided in Supplementary Data 28. RF Random Forest. SG: model trained with SANGER Cell Model Passports data as cell-line input data.

For this test, all DRP models were trained using the entire GDSC1 + 2 dataset without splitting it into separate training and testing subsets to maximize the available data. We excluded DRP models which suffer from the paucity in gene expression data (PaccMann) or show limited utility in drug-blind scenarios (DRPreter_SA, DRPreter_SA_SG). Consequently, all models showed higher RMSEs than even those from the strict-blind tests because of chemical heterogeneities and technical differences among the IC_50_ measurements in GDSC and ChEMBL (Fig. 4a–c; Supplementary Fig. 27; Supplementary Table 8; Supplementary Data 28, 29). In general, HiDRA outperforms the other models across RMSE and PCC, with GCNPath performing next best. Compared to other strong models such as DRPreter and TGSA, GCNPath achieved lower RMSE and slightly higher and more stable PCC and SCC values. PaccMann_SG exhibited extremely poor RMSE scores for certain model weights, and consistently lower PCC and SCC across all weights compared to other models, indicating its limited generalizability in drug-blind scenarios.

For DRP models to be universally applicable, they should provide reliable predictions using external cell data with different distributions from the training set. As noted in the “Introduction”, gene expression datasets have distinct distributions due to not only biological signals but also technical biases, which challenge DRP models relying on gene expression inputs. To evaluate which models maintained the most stable performance insensitive to these batch effects, we tested the models using gene expression datasets as inputs from 404 cell lines across GDSC, CCLE, and SANGER Cell Model Passports (Supplementary Fig. 28). Here, GCNPath consistently showed superior predictive performance for external cell line datasets (Fig. 4d–f; Supplementary Figs. 29–31; Supplementary Table 9; Supplementary Data 28, 30–32). HiDRA and PaccMann_SG exhibited significantly weaker performance than the other models, including our GCNPath model.

**Fig. 5:**
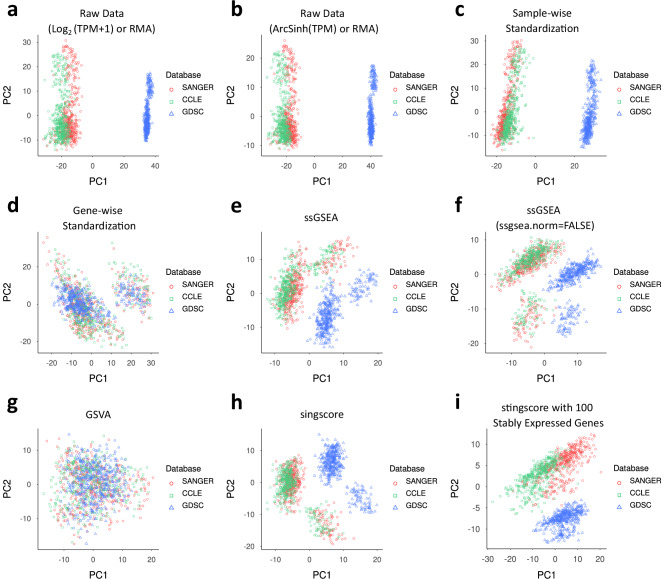
Visualization of cell-line gene expression datasets using various feature processing methods. The 404 cell lines common in TPM data from SANGER Cell Model Passports, CCLE, and the microarray data from GDSC were utilized to predict ln(IC_50_) values from ChEMBL, respectively. With these overlapping cell lines, distribution of these three datasets were visualized with principal component analysis (PCA) plots (*n* = 404 for each panel, respectively). Note that except the ArcSinh normalization, TPM data from SANGER and CCLE were basically normalized in log2-scale with pseudo-count 1. The source data are provided in Supplementary Data 33. **a** No_Norm: Raw counts with no additional normalization methods applied beyond log_2_ transformation in TPM data or RMA in microarray data; **b** ArcSinh: Raw counts with no additional normalization methods applied beyond ArcSinh transformation in TPM data or RMA in microarray data; **c** Z_Sample: z-score standardization of gene expression within a cell line; **d** Z_Gene: z-score standardization of gene expression for each gene across all cell lines; **e** ssGSEA: pathway activation scores calculated with single-sample gene set enrichment analysis; **f** ssGSEA_Norm_X: ssGSEA without across-sample normalization (parameter ssgsea.norm=FALSE); **g** GSVA: pathway activation scores calculated with gene set variation analysis; **h** singscore: pathway activation scores calculated with simple single-sample gene signature scoring; **i** stingscore: singscore calculated using 100 genes showing stable expression across various cancers; included in the 292 BIOCARTA pathways and the TPM data from SANGER Cell Model Passports. Of these genes, 100 and 93 overlapped with CCLE and GDSC datasets, respectively.

To determine the impact of batch effects on DRP performance, we analyzed cell-line feature preprocessing methods (Supplementary Table 10). As mentioned in “Results”, GCNPath uses GSVA pathway scores, summarizing gene expression into pathway-level metrics. PaccMann applies arcsine normalization to TPM values, while HiDRA uses sample-wise standardization, and other models rely on gene-wise standardization. Random Forest does not apply any normalization to the gene expression inputs. If these preprocessing methods effectively mitigate batch effects, gene expression data across datasets should align closely, with identical cells mapping similarly across different sources. Our observations showed that GSVA scores and gene-wise standardization effectively mitigated batch effects in TPM data from SANGER Cell Model Passports, CCLE, and microarray data from GDSC (Fig. 5; Supplementary Data 33). These methods align gene expression data across the same cell lines across distinct RNA datasets, while differentiating data from different cell pairs (Supplementary Fig. 32; Supplementary Data 34). This suggests that GSVA and gene-wise standardization are robust against batch effects and effectively capture biological signals. Notably, these methods outperform other unsupervised pathway-based feature reduction methods like ssGSEA^29^, singscore^30^, and stingscore^31^. This capability enhances the versatility of GCNPath and other gene-based models, especially when query cell line data is absent in the training dataset but present in external datasets.

Beyond predictive performance, we also measured the inference time of each model on ChEMBL dataset. While GCNPath was not the fastest, it exhibited a reasonably efficient prediction speed, suggesting its suitability for large-scale drug screening tasks (Supplementary Fig. 33; Supplementary Data 35).

### GCNPath could be partially applicable to clinical data

To enable the clinical application of DRP models for treatment selection, it is essential to evaluate their performance in predicting clinical drug responses in addition to cell line predictions. Shen et al. ^6^ assessed the performance of nine models using multi-omics data and clinical response information from TCGA patients treated with monotherapy. By analyzing prediction differences between responders and non-responders, they concluded that current cell-line-based models fail to accurately predict clinical responses.

Following a similar approach, we predicted drug responses for 414 cases (399 patients, 11 drugs). TPM data from TCGA were processed into GSVA scores, matching the procedure used for cell line data. Batch effects between TPM data from SANGER Cell Model Passports and TCGA were mitigated, although TCGA data showed greater inter-tumor heterogeneity (Supplementary Fig. 34; Supplementary Data 36). Patients with complete or partial responses (CR/PR) were classified as responders, while those with stable or progressive disease (SD/PD) were classified as non-responders. Most models trained ten times on the entire GDSC1 + 2 as a target label dataset without data splits, were used for predictions. Due to limited response data per drug, predictions were not averaged across models. The ability to distinguish responders from non-responders was evaluated.

**Fig. 6:**
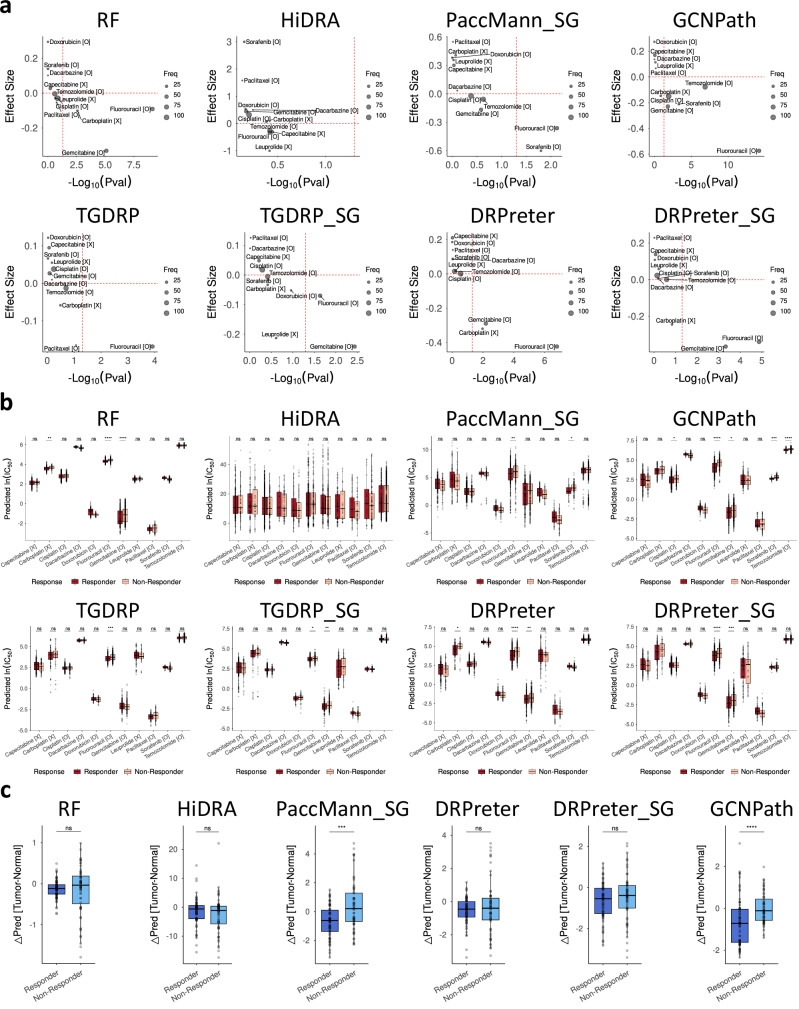
Benchmark test with clinical data from the TCGA dataset, without batch correction with reference ComBat. Most models were trained ten times using the entire GDSC1 + 2 dataset before application. tCNNS and GraphDRP were excluded because their required input features are not available in TCGA dataset. Additionally, TGSA, TGSA_SG, DRPreter_SA, and DRPreter_SA_SG—which are unable to predict drugs not included in GDSC dataset—were also not included. Since the response data for each drug were limited, the predictions were not aggregated across models (*n* = 11 drugs for each panel in (**a**); *n* = 414 clinical responses for each panel in (**b**); *n* = 13 clinical responses for each panel in (**c**)). In (**a**,** b**), ‘O’ and ‘X’ in brackets show whether the drugs were present in GDSC1 + 2 dataset (e.g., cisplatin [O] was screened in GDSC). In (**a–c**), *p* values were obtained from a one-tailed Mann–Whitney test, and effect sizes in (**a**,** b**) were calculated as the difference in average predictions between responder and non-responder classes. Drugs with significant predicted effectiveness for responders appear in the bottom right (*p* < 0.05, effect size < 0). Other regions indicate low predicted significance (left) or inconsistency between predicted and actual responses (up). In (**c**), the prediction distributions across 13 tumor and matched normal sample pairs were shown. For TGDRP and TGDRP_SG, mutation and copy-number variation (CNV) profiles from matched-normal samples were unavailable. In (**b**,** c**), boxes span the IQR, with whiskers extending 1.5×IQR. Source data are provided in Supplementary Data 37. RF random forest. SG: model trained with SANGER Cell Model Passports data as cell-line input data. *: *p* ≤ 0.05, **: *p* ≤ 0.01, ***: *p* ≤ 0.001, ****: *p* ≤ 0.0001.

GCNPath achieved the best performance, significantly distinguishing responders for five drugs: Fluorouracil (*P* = 6.3 × 10^−15^, effect size = −0.574), Gemcitabine (*P* = 1.5×10^−2^, effect size = −0.229), Sorafenib (*P* = 4.9 × 10^−4^, effect size = −0.211), Cisplatin (*P* = 1.1 × 10^−2^, effect size = −0.150) and Temozolomide (*P* = 1.3×10^−7^, effect size = −0.078) (Fig. 6a, b; Supplementary Data 37). Predictive performances were poorer for drugs prescribed to fewer patients, potentially reducing statistical significance and effect sizes in identifying well-treated groups. The superiority of GCNPath remained consistent even after correcting for batch effects in the gene expression data for each model using reference ComBat^32^ (Supplementary Fig. 35; Supplementary Data 38). Before and after applying reference ComBat, DRPreter, and Random Forest, as well as TGDRP and Random Forest, achieved the second-best performance, each correctly predicting responses to three and four drugs, respectively. According to GCNPath predictions, differences in predicted responses between tumor and matched normal tissues from 13 patients were significantly greater in responders than in non-responders (*P* = 1.1 × 10^−5^, Fig. 6c; Supplementary Data 37). These findings suggest that GCNPath partially bridges the gap between cell-line-based and clinical data in DRP.

### Validation of GCNPath with pre-established therapies in colorectal and breast cancers

To assess the practical applicability of GCNPath, we evaluated drug–response relationships established in colorectal and breast cancers, both with abundant cell-line expression profiles in Sanger Cell Model Passports. Analyses focused on subtype memberships and their associated biomarkers.

**Fig. 7:**
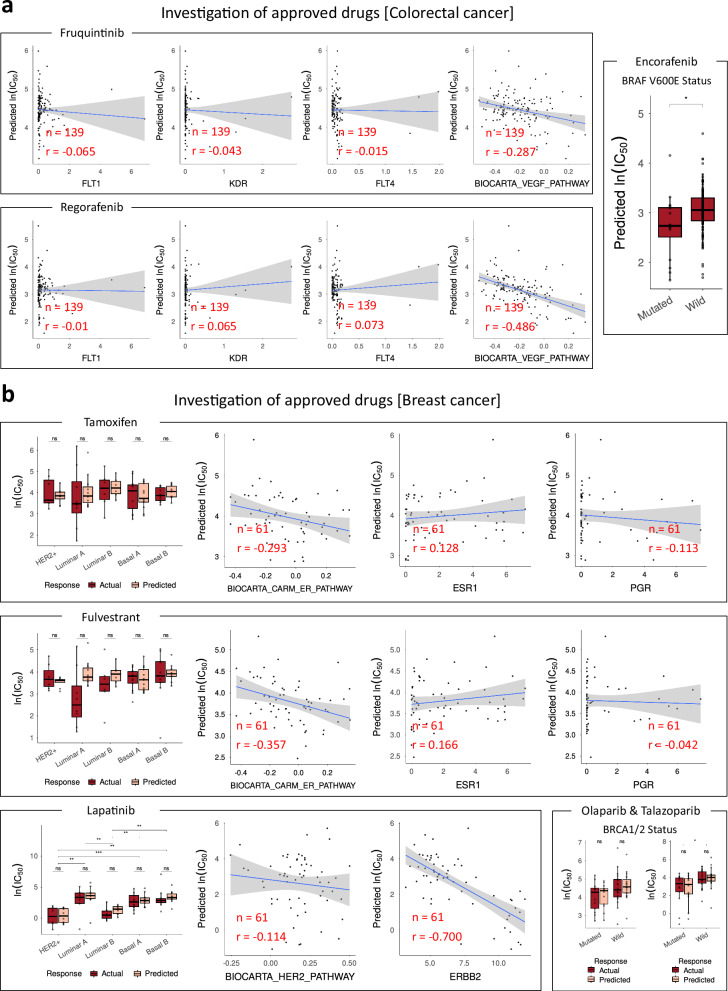
Investigation of cell-line-level prediction values with cancer subtypes and their associated biomarkers. All GCNPath models trained ten times with the entire GDSC1 + 2 as a target label dataset were utilized, and prediction values were averaged across models. Drug response predictions for approved therapies in (**a**) colorectal and **b** breast cancers were evaluated using 139 and 61 cell-line gene expression data from Sanger Cell Model Passports (*n* = 139 and 61 for each panel, respectively). Correlation values were calculated between predicted sensitivities and subtype biomarker expression, and boxplots were generated for cell lines with available subtype memberships or mutation status. Note that only 16 colorectal cancer cell lines and 13 breast cancer cell lines harbor BRAF V600E and BRCA1/2 mutations, respectively. In boxplots, FDRs for subtype comparisons and *p*-values for mutation status were obtained from a two-tailed Mann–Whitney test, and boxes span the IQR, with whiskers extending 1.5×IQR. Source data are provided in Supplementary Data 39. *: *p* or FDR ≤ 0.05, **: *p* or FDR ≤ 0.01, ***: *p* or FDR ≤ 0.001.

In colorectal cancer, consensus molecular subtypes (CMS) are characterized by druggable biomarkers such as VEGFR and the BRAF V600E mutation^33^. Across 139 cell lines, we tested three approved therapies (Fig. 7a; Supplementary Data 39). Fruquintinib and Regorafenib, both VEGFR inhibitors, showed negative correlations with VEGF pathway activity, though not with individual VEGFRs. Encorafenib was effective in BRAF V600E–mutant cell lines, suggesting that the model captured mutation-driven sensitivity despite not directly using mutation status as input.

In breast cancer, subtypes are defined by estrogen and progesterone receptor (ESR1 and PGR), and ERBB2^34^. Using 61 cell lines, five approved drugs were evaluated (Fig. 7b; Supplementary Data 39). Tamoxifen and fulvestrant showed no statistically significant selectivity for hormone-rich subtypes, likely due to training constraints that align predictions with observed ln(IC_50_) distributions. Nonetheless, high estrogen-pathway activity was linked to greater vulnerability. HER2-positive subtypes were sensitive to lapatinib, and HER2-negative subtypes with BRCA1/2 mutations were predicted to respond to olaparib and talazoparib.

Together, these results indicate that GCNPath could recapitulate drug-specific modes of action and holds potential utility for therapeutic decision-making by integrating gene- and pathway-level expression patterns.

### Application of GCNPath to investigate heterogeneity in drug responses according to cancer subtypes and drug targets in SCLC

After confirming the practical utility of GCNPath, we conducted a case study on small cell lung cancer (SCLC). Identifying distinct predicted drug responses based on cancer subtype or drug target expression can aid in selecting treatment strategies for patients and repurposing drugs for cancers with varying statuses or subtypes. We focused on SCLC^35^, which is notoriously difficult to treat due to rapid proliferation and early metastasis. The low survival rates associated with existing SCLC therapies may stem from overlooking molecular heterogeneity, underscoring the need for accurate DRP models. To address this, we first validated GCNPath using both standard and promising therapies for SCLC and further investigated optimal treatment strategies across molecular subtypes, referring to recent clinical findings by Liu, Qian, et al. ^36^.

**Fig. 8:**
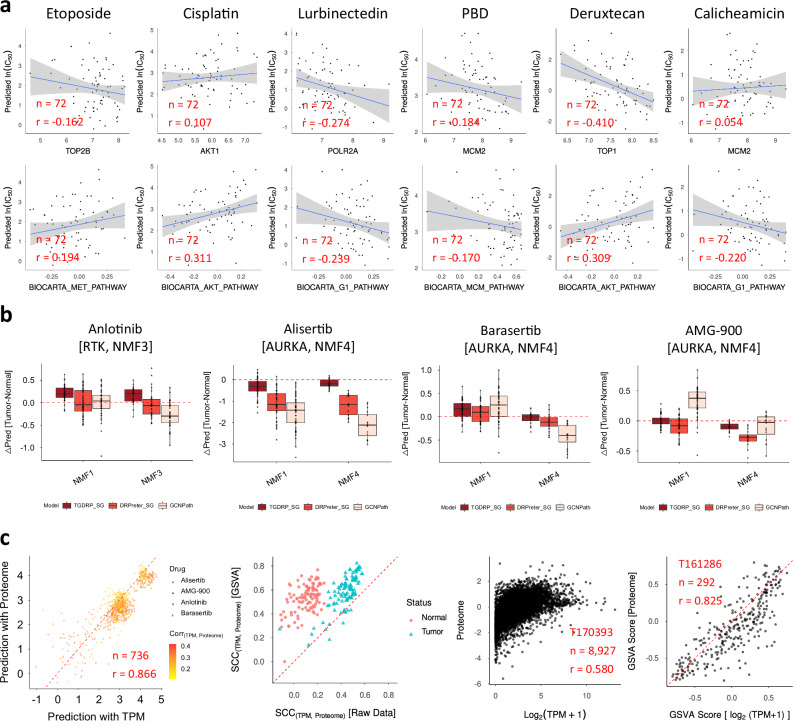
Application of GCNPath in SCLC with cell-line-based and clinical data. All GCNPath models trained ten times with the entire GDSC1 + 2 as a target label dataset were utilized, and prediction values were averaged across models. **a** For the cell-line-based analysis using 72 cell-line gene expression data from Sanger Cell Model Passports, drug response predictions were generated for three approved therapies—etoposide, cisplatin, and lurbinectedin—and three emerging antibody-drug conjugates (ADCs): pyrrolobenzodiazepine (PBD), deruxtecan, and calicheamicin (*n* = 72 for each panel). Correlation values were computed between predicted ln(IC_50_) values and the expression levels of corresponding targets or sensitivity- and resistance-associated biomarkers. MCM pathways represent DNA replication process. **b** For the clinical analysis using gene expression data from Liu et al. ^36^, drug responses to four compounds were predicted across tumor and matched-normal samples from patients classified as NMF1, NMF3, and NMF4 (*n* = 45, 31, and 16, respectively). Known targets and optimal subtypes for each drug are summarized. For TGDRP and TGDRP_SG, mutation and copy-number variation (CNV) profiles from matched-normal samples were unavailable, therefore zero-imputed. **c** GCNPath was further applied to proteomic data from the same patient cohort. Left: correlation analysis between prediction results using gene expression data (TPM) and proteome-derived GSVA scores. Middle left: correlation values at both raw and GSVA levels for each patient. Middle right and right: representative scatter plots comparing gene expression data and proteome data in two samples with the highest correlation at the raw (T170393) and GSVA (T161286) levels, respectively. Except for the left panel, a total of 214 tumor and matched-normal samples from 107 patients were utilized. Source data are provided in Supplementary Data 40.

We predicted drug responses for three well-established therapies—etoposide, cisplatin, and lurbinectedin—and three emerging antibody-drug conjugates (ADCs)—pyrrolobenzodiazepine (PBD), deruxtecan, and calicheamicin—using gene expression data from 72 SCLC cell lines in Sanger Cell Model Passports. Global correlation trends were examined between predicted drug sensitivities and the expression of known targets or biomarker genes (Fig. 8a; Supplementary Data 40). As expected, resistant markers showed positive correlations with predicted ln(IC_50_), while drug targets or sensitivity-associated markers showed negative correlations. For instance, the MET pathway contributes to therapeutic resistance against etoposide^37^, while the AKT pathway has been implicated in reduced drug sensitivity to cisplatin^38^ and deruxtecan^39^.

In addition to correlation analysis, we employed Gradient-based Class Activation Mapping (Grad-CAM) to compute pathway importance scores and enhance the interpretability of GCNPath at the individual prediction level. For each drug–cell line pair with high predicted sensitivity, the top five contributing pathways were identified (Supplementary Table 11). Notably, several of these pathways aligned with established findings in the literature. For instance, in the etoposide–LB647-SCLC pair, the p38 MAPK pathway was highlighted; it is reported that downregulation of this pathway enhances etoposide efficacy^40^. For cisplatin–NCI-H748, the MET pathway emerged as a key contributor, consistent with evidence that c-MET inhibition may overcome cisplatin resistance by suppressing AKT/mTOR signaling^41^. The NF-κB pathway was implicated in predictions for both PBD and calicheamicin, aligning with its known role in mediating multi-drug resistance via ABCB1 transporters^42^. These findings suggest that GCNPath effectively captures the molecular landscape underlying drug responses at both population- and sample-level resolutions.

Next, we applied GCNPath to gene expression data from 214 SCLC tumor and matched-normal samples derived from 107 patients in the study by Liu, Qian, et al. ^36^. Although several studies have proposed four molecular subtypes of SCLC—collectively termed ANPI—based on the expression of ASCL1, NEUROD1, and POU2F3^43,44^, these classifications have not been well validated in terms of therapeutic response. Therefore, we focused on subtypes identified in a relatively large clinical cohort with both multi-omics profiling and associated drug response data. In that study, four molecular subtypes (NMF1–NMF4) were defined, and two key drug–response findings for comparison between subtypes were experimentally validated: NMF3 was more sensitive to anlotinib (RTK inhibitor) than NMF1, and NMF4 was more vulnerable to AURKA inhibitors than NMF1.

In our prediction task, we compared GCNPath to TGDRP_SG and DRPreter_SG, which had previously demonstrated the most favorable and balanced performance in our benchmark tests. Like TCGA dataset, GSVA alleviated batch effects between SANGER Cell Model Passports and the clinical dataset (Supplementary Fig. 36; Supplementary Data 41). Using the four drugs tested in the Liu et al. ^36^ study, GCNPath successfully recapitulated the sensitivity of NMF3 and NMF4 to anlotinib and AURKA inhibitors, respectively, relative to NMF1 (Fig. 8b; Supplementary Fig. 37; Supplementary Data 40, 42). Additionally, we evaluated the versatility of GCNPath by applying it to proteomic data from the same patient cohort. Predictions based on GSVA scores derived from both gene expression data (TPM) and proteomic data were well correlated, suggesting that GCNPath has the potential to be extended to protein-based applications (Fig. 8c; Supplementary Data 40). This finding is particularly meaningful given that mRNA expression levels do not always directly reflect protein abundance^45,46^.

## Discussion

We developed GCNPath to address key challenges in the cell-line-based DRP. GCNPath models pathway interactions through PCN graphs processed with a GCN architecture, enhancing performance via pathway-based feature extraction. We focused on evaluating the model’s adaptability to unseen cell lines and drugs, with comprehensive benchmarks including strict-blind tests on two GDSC versions and external tests using ChEMBL dataset. Fairness was ensured by carefully considering the number of cell-line data input for training and validating predictions using models pretrained by each model developer. In these tests, GCNPath outperformed other models in diverse scenarios, including drug-blind and strict-blind tests, effectively capturing biological signals from several RNA datasets. This enabled robust predictions for clinical TCGA datasets and revealed inter-heterogeneity in drug responses across SCLC subtypes. These findings highlight the practical utility of GCNPath in enhancing the precise DRP, its adaptability to unseen scenarios, and its ability to uncover biologically meaningful insights, showing potential for broader applications in drug recommendation and discovery.

To further assess the competence of GCNPath, we explored the relationship between deep learning architectures and benchmark results. Our findings confirm that advanced architectures, particularly GCNs and attention modules, significantly enhance performance. However, leveraging cell and drug similarities based on gene expression and chemical structures, as seen in TGSA and DRPreter, provided minimal benefit. Through benchmark studies with GDSC datasets, we found that drug characteristics, cell line traits, and the quality of drug response data influenced predictive performance. These insights could guide future improvements in DRP models.

Our study has several limitations, which are common to all DRP models or beyond the scope of this study to address. First, like all deep learning models, ours shows weaknesses in drug-blind and strict-blind tests, likely due to the vast chemical feature space^7,11^. Our extensive benchmark tests with GDSC datasets revealed that expanding the drug set with GDSC1 + 2 did not notably improve performances for unseen drugs. Additionally, TGSA and DRPreter_SA, which incorporate drug similarity augmentation, showed limited benefits. Some models use drug-related information, such as drug targets, to tackle these challenges^21,47^, but directly incorporating such data may reduce model generalizability, especially for drugs lacking this information. Other approaches, such as pretrained drug modules^48^, additional drug response datasets^49^ or chemically perturbed gene expression data^50^, have been explored. Future benchmark tests will help determine which strategies most effectively enhance predictions for unseen drugs.

Second, persistent challenge for all DRP models is the scarcity of non-cancer cell line data in training datasets^51^. Many DRP models aim to be used in personalized medicine^11–13^. However, the goal of precision medicine is to recommend drugs that target cancer cells while minimizing harm to normal cells. Fortunately, leveraging around one thousand cell lines in GDSC, CCLE, and SANGER Cell Model Passports, DRP models learn how drug responses differ according to genetic mutation or expression status and therefore might be able to accurately infer the drug responses of non-cancer cells. Thus, predicting non-cancer cell responses may be as feasible as other cell-blind prediction tasks, which are easier than drug-blind tasks.

Lastly, our model is based on monotherapy data and is not directly applicable to more complex and clinically relevant tasks, such as predicting combinatory drug effects or responses to perturbed gene expression. However, several strategies could expand its versatility. For example, correlation analysis of predicted monotherapy responses across drug pairs could indirectly identify potentially optimal anticancer combination^51^. Additionally, incorporating transfer learning with perturbed gene expression profiles^52^ may further enhance the model’s adaptability to complex therapeutic scenarios. We also acknowledge that clinical data generally present more complex molecular landscapes than cell-line data, which complicates the direct application of DRP models. Nevertheless, DRP models may still serve as useful tools for primary screening to identify plausible drug candidates and may provide preliminary insights into their modes of action.

One caution in using our model is its applicability to certain external datasets. Both Precily^20^, a pioneer model that utilizes GSVA scores as cell-line features, and our model partially address the batch effects between RNA datasets. However, GSVA calculates pathway scores based on whether genes are over- or underexpressed within the context of sample populations, making it prone to errors when processing datasets with few or biased samples^30^. Nevertheless, we expect that the versatility of our model could be enhanced by exploiting more advanced pathway-based feature extraction, which not only corrects batch effects but also compensates for the weakness of GSVA.

## Methods

### Processing of cell line data

Cell line RNA-seq data were obtained by downloading TPM count data from SANGER Cell Model Passports database (v.2.9.0)^53^. Out of 1431 total cell lines, 972 had corresponding ln(IC_50_) values in GDSC1 + 2 dataset (Supplementary Data 43). TPM values were transformed into log2-scale values with a pseudo-count of 1. Gene lists from 292 BIOCARTA pathways were sourced from the MSigDB database (v.2023.1. Hs)^54^, with 1506 out of 1509 genes present in the TPM data. Genes not included in these pathways were filtered out, and the remaining TPM values were converted to pathway-level expression data using the gsva function in the GSVA R package (v.1.44.5)^22^ with default parameters. For biological interactions, networks were extracted from STRING (v.11.5)^55^ and RegNetwork^56^. For STRING, interactions with confidence scores of at least 0.7 (STRING_700) and 0.9 (STRING_900) were extracted. All interaction edges from the human GRN in RegNetwork were retained, as it lacks confidence scores.

To represent pathway interactions as a graph, we compressed the two networks using a separation score, as proposed in previous studies^23,57^ (Fig. 1a). The separation score (${{{{\boldsymbol{s}}}}}_{{{{\boldsymbol{AB}}}}}$) quantifies the distance between two pathways by calculating the difference in the average shortest distances within and between gene sets as follows:

1 $${{{{\boldsymbol{s}}}}}_{{{{\boldsymbol{AB}}}}}\equiv {{\langle }}{{{{\boldsymbol{d}}}}}_{{{{\boldsymbol{AB}}}}}{{\rangle }}-\frac{{{\langle }}{{{{\boldsymbol{d}}}}}_{{{{\boldsymbol{AA}}}}}{{\rangle }}+{{\langle }}{{{{\boldsymbol{d}}}}}_{{{{\boldsymbol{BB}}}}}{{\rangle }}}{{{{\bf{2}}}}}$$

where ${{{{\boldsymbol{d}}}}}_{{{{\boldsymbol{XY}}}}}$ is the mean of shortest distances between pathway X and Y. If no gene pair within and/or between gene sets is connected, no separation score is calculated for that pathway pair. Gene overlap ratios were also calculated as follow:

2 $${{{{\boldsymbol{OR}}}}}_{{{{\boldsymbol{AB}}}}}=\frac{{{{{\boldsymbol{g}}}}}_{{{{\boldsymbol{A}}}}}\cap \,{{{{\boldsymbol{g}}}}}_{{{{\boldsymbol{B}}}}}}{{{{{\boldsymbol{g}}}}}_{{{{\boldsymbol{A}}}}}\cup \,{{{{\boldsymbol{g}}}}}_{{{{\boldsymbol{B}}}}}}$$

where ${{{{\boldsymbol{g}}}}}_{{{{\boldsymbol{X}}}}}$ is the number of genes in pathway X.

In this way, PCN graphs were generated from the STRING_700, STRING_900 and RegNetwork networks via the distances function in the igraph R package (v.1.3.5)^58^. These networks were simplified by trimming into k-nearest neighbors (KNN) graphs, retaining only the closest pathways in each pathway based on the top K lowest separation scores (K = 5 in our study). When fewer than K separation scores were available, the remaining pathways were chosen based on the highest gene overlap ratios. Additionally, we generated a pathway correlation network derived from GSVA pathway scores, which was also processed into a KNN network with the same K value as STRING_900 and RegNetwork (Fig. 1a). STRING_700 and STRING_900 had 75.89% edge overlap, and STRING_900 was selected due to its higher confidence scores. The edges in the PCN graphs are listed in Supplementary Data 44.

We highlight two topological properties of PCN graphs. First, the KNN approach introduces edge directionality, as neighbor relationships are not always reciprocal. For example, pathway A may select B, C, D, E, and F as its nearest neighbors, while pathway B may choose a different set, such as C, E, G, P, and X. In this case, an edge from A to B exists, but not from B to A, illustrating the inherently asymmetric structure of the KNN-based network. Second, PCN graphs typically reflect horizontal interplays between pathways, rather than hierarchical relationships as in Gene Ontology (GO). Most gene set databases, such as BIOCARTA and KEGG, do not explicitly represent inter-pathway communication. However, PCN graphs can be constructed from any pathway database, if pathways are directly or indirectly linked through gene- or protein-level networks.

Finally, the GSVA features of the cell lines were standardized via the RobustScaler function in the scikit-learn python package (v.1.2.2)^59^, with z-scores exceeding an absolute value of ten capped. These steps ensure model training is insensitive to varying value scales between pathways and outliers. The normalized GSVA scores and the PCN graphs were integrated via the Data function in the PyTorch Geometric python package (v.2.1.0)^60^.

### Processing of drug data

For the drugs listed in GDSC database, PubChem^61^ compound identifiers (CIDs) were retrieved to obtain structural information in simplified molecular input line entry system (SMILES) format. We utilized the PubChem Identifier Exchange Service and get_cid function in the webchem R package (v.1.2.0)^62^. For drugs without CIDs, annotations from the Harvard Medical School–Library of Integrated Network-Based Cellular Signatures (HMS-LINCS)^63,64^ and a manual Google search using drug synonyms and/or targets were used. In total, 432 unique drug CIDs and corresponding SMILES structures were obtained (Supplementary Data 45). From these SMILES structures, we extracted physicochemical features, including atom and bond types, atom masses and bond stereotypes (Supplementary Table 12), via the dgllife python package (v.0.2.9)^65^. Finally, 85 atom and 10 bond features were integrated via the Data function in the PyTorch Geometric python package (v.2.1.0)^60^.

### Processing of IC_50_ data

GDSC database (Release 8.4)^2^ contains a total of 576,758 drug response data points in ln(IC_50_ μM) format, covering 988 unique cancer cell lines and 624 drugs. GDSC2 offers a broader drug concentration range (1000- or 1024-fold ratio vs. 256-fold ratio in GDSC1), enabling more precise IC_50_ estimation. We downloaded IC_50_ data from both datasets but used only those for the 432 drugs with available SMILES structures. For multiple values for a single cell line and drug combination, the average was used (Supplementary Fig. 38a, b; Supplementary Data 46). When data were present in both GDSC1 and GDSC2, we use only GDSC2 data following GDSC web portal’s recommendation (Supplementary Fig. 38c; Supplementary Data 46). In total, we selected 266,573 IC_50_ data points from GDSC1, 199,687 from GDSC2, and 373,681 from the combined GDSC1 + 2 datasets (Table 1; Supplementary Data 47–49).

**Table 1:** The numbers and summary statistics of ln(IC_50_) values obtained from GDSC1 + 2, GDSC1, GDSC2 and ChEMBL

	GDSC1 + 2	GDSC1	GDSC2	ChEMBL
Number of screened cells	978	970	969	404
Number of screened drugs	432	321	236	112,942
Number of ln(IC_50_) values	373,681	266,573	199,687	237,580
Number of duplicates	185,158	39,278	15,326	33,213
Number of duplicates after averaging	92,579	19,231	7,663	12,976
RMSD between duplicates	0.945	0.854	0.896	1.250
PCC between duplicates	0.938	0.946	0.962	0.930

* The root mean square deviations (RMSDs) and PCCs between the duplicated ln(IC_50_) values and their average values were calculated.

For external validation, we mined IC_50_ values from ChEMBL database (Release 33)^17^ using ChEMBL web resource client python package (v.0.10.8). Of the 1,483 human cell lines in ChEMBL, we identified SANGER, BROAD and/or COSMIC IDs for 915 cell lines by querying their names and Cellosaurus IDs. This approach allowed us to select cell lines with corresponding gene expression data from SANGER Cell Model Passports, GDSC and CCLE. IC_50_ values for these cell lines were converted to ln(IC_50_ μM) format, excluding high-throughput screening data from GDSC and NCI-60, as well as compounds already screened in GDSC1 + 2. Multiple ln(IC_50_) values for each cell line and drug combination were averaged. In total, we retrieved 237,580 values (Table 1**;** Supplementary Fig. 39a; Supplementary Data 50). The drug information and the ln(IC_50_) values from ChEMBL are listed in Supplementary Data 51 and 52, respectively. The ln(IC_50_) values from ChEMBL exhibit a similar distribution to those from GDSC1 + 2 via density plot visualization (Supplementary Fig. 39b; Supplementary Data 50).

### Processing of TCGA data

RNA-seq TPM and patient clinical data were downloaded from the TCGA database (v.37.0)^66^ via the TCGAbiolinks R package (v.2.25.3)^67^. Since the data are de-identified and publicly available, no additional ethical approval or patient consent was required. For TPM data, we converted gene IDs from ENSEMBL to ENTREZ format, normalized to log2 scale with pseudo-count 1, and calculated GSVA pathway scores for all 11,274 patients. GSVA scores were standardized with the RobustScaler fitted for TPM data from SANGER Cell Model Passports, using the fit_transform function in the scikit-learn Python package (v.1.2.2)^59^.

From clinical data, we selected patients with monotherapy records and unique treatment response data as clinical endpoints. We classified complete responses (CR) and partial responses (PR) as responders, and stable disease (SD) and progressive disease (PD) as non-responders, following Shen et al. ^6^ Drugs with multiple names were unified using the GDISC database^68^. PubChem CIDs for each drug were retrieved via the get_cid function in the webchem R package (v.1.2.0)^62^ and manual searches. Patients without TPM data and drugs with fewer than 10 response data points were excluded, leaving 399 patients, including 15 with duplicate samples (2 metastasis and 13 matched normal tissue samples). In total, 414 response data points for 11 drugs across 399 patients were included. The drug information and response values from TCGA are listed in Supplementary Data 53 and 54. Several discussions and limitations as for the treatment responses were depicted in Supplementary Note 2.

### Processing of SCLC clinical data

RNA-seq TPM, proteome data and subtype information for 107 patients were obtained from the Liu, Qian, et al. ^36^ via their supplementary tables (Table S1 and S6A). For TPM and proteome data, a total of 1452 and 1019 genes were included in 292 BIOCARTA pathways. Data preprocessing followed the steps in “Method 1–4”, except gene symbols were retained and not converted to ENTREZ format.

### GCNPath architecture

The GCNPath model comprises three modules: the cell module, the drug module, and the prediction module (Fig. 1b). The cell module utilizes three RGCN^24^ layers to process the three types of relations in the PCN graphs for each cell line. For model interpretability, we adopt Grad-CAM on the first hidden layer of the cell module, which calculates pathway importance values via backpropagation. The drug module applies three GAT^69^ layers with four attention heads emphasize key neighboring nodes in atom‒bond graphs through a multi-head self-attention mechanism.

To mitigate the over-smoothing issue in GCNs, both modules adopt the DenseGCN architecture^70^, inspired by the DenseNet framework^71^ in CNNs. This architecture concatenates all previous hidden features at each layer, preserving information from all GCN depths and reducing sensitivity to the number of layers. Hidden dimensions were set to [8, 8, 8] for the cell module and [128, 128, 128] for the drug module, yielding final dimensions of 24 and 378, respectively. These were summarized into cell and drug embeddings. For cell line graphs, we concatenated all pathway embeddings into one-dimensional vectors, as proposed in TGDRP model^11^, instead of applying global pooling. For drug graphs with varying atom counts, atom embeddings were aggregated using global max pooling. The resulting embeddings were each passed through a single FCN layer to produce 256-dimensional vectors.

Finally, the cell line and drug embeddings were then concatenated into a 512-dimensional vector and passed through two FCN layers in the prediction module to predict the ln(IC_50_) values. The model architecture was constructed using PyTorch and PyTorch Geometric python packages (v.1.11.0 and v.2.1.0, respectively)^60,72^.

### Ablation tests

Ablation tests for GCNPath were performed using GDSC2 as a target label dataset in both unblinded and strict-blind tests. The tests compared GCN and FCN architectures, examining whether interactions between pathways and atoms in PCN and atom-bond graphs were considered. The FCN architecture in the cell or drug module, which ignores graph structures, utilized GSVA pathway scores or one of three types of one-dimensional fingerprints as inputs: Morgan fingerprints calculated using the GetMorganFingerprintAsBitVect function from the RDKit Python package (v.2022.09.5)^25^ with parameters nBits = 128, 256, 512, 1024 and radius = 1, 2, 3; and a 100-bit SMILESVec fingerprint, derived from the SMILESVecProteinRepresentation GitHub repository, utilizing word-level embeddings trained with ChEMBL23 and PubChem (available at https://cmpe.boun.edu.tr/~hakime.ozturk/smilesvec.html).

### Benchmark tests with GDSC datasets

To evaluate the DRP models comprehensively, we conducted four types of performance tests: unblinded, cell-blind, drug-blind, and cell- and drug-blind (strict-blind) tests (Fig. 1c). In unblinded tests, the ln(IC_50_) values were split into training, validation and testing datasets at an 8:1:1 ratio to ensure that drug response values for each cell line and drug were represented in approximately the same proportions. For the cell-blind and drug-blind tests, the cell lines and drugs, along with their corresponding ln(IC_50_) values, were split at an 8:1:1 ratio. For the strict-blind tests, the cell lines and drugs were divided at a 3:1:1 ratio each, resulting in ln(IC_50_) splits of approximately 9:1:1. Since different splits were used for the strict-blind test and the remaining tests, different numbers of cross-validations are required (25 for the strict-blind test and 10 for the others, respectively). Each test was implemented using GDSC1, GDSC2 and GDSC1 + 2 as target label datasets. Detailed information on preparing omics datasets and training for each model is provided in Supplementary Note 3.

Model performances were evaluated using RMSE, PCC, and SCC metrics. Training epochs were capped at 300 with an early stopping patience of 10 epochs to prevent overfitting. If validation performance (measured by mean squared error, MSE) did not improve within the patience, training was halted. Differences in performance were assessed via pairwise one-tailed Mann‒Whitney tests using the wilcox.test function from the stats R package (v.4.5.0). The alternative = “less” parameter was used for RMSE comparisons, while alternative = “greater” was applied for PCC and SCC comparisons. We also employed pretrained models provided by their developers, if available, to predict GDSC datasets. Further details on pretrained models are included in Supplementary Note 3.

### Analysis of factors affecting DPR performances

Several factors influencing DRP performance were examined, inspired by the study of Shen et al. ^6^ For (1), prediction values from all DRP models trained in Methods (“Benchmark tests with GDSC datasets”) with all test scenarios were utilized. For (2–3), only GCNPath trained with unblinded tests in were considered.

#### Correlation analysis of prediction errors between DRP models

For the RMSE_C_ and RMSE_D_ values calculated as described above, pairwise PCC were computed between the RMSEs of different models for each test type.

#### Individual characteristics of cell lines and drugs

The count, mean, standard deviation, bimodality, and density coverage of ln(IC_50_) values from GDSC1 + 2 were calculated for each cell line and drug. These were compared with the SCC_C_ and SCC_D_ values derived from the prediction values of GCNPath. Additionally, cell-line and drug annotation files from GDSC were used to obtain tissue type and target pathway information. SCC values were also calculated separately for each tissue type and target pathway, then compared with the number of ln(IC_50_) values for the same groups.

#### Intrinsic variations of IC_50_ measurement

The 11,670 IC_50_ values from CCLE were obtained from the “Pharmacological Profiling Files” section of DepMap. These IC_50_ data were then natural log-normalized similar to GDSC data. Drug response values capped at 8 μM were excluded beforehand. From these, 2561 response values for 374 cell lines and 18 drugs, which were also measured in GDSC, were extracted. The relationship between the absolute difference in ln(IC_50_) values between GDSC and CCLE and the prediction error from GCNPath was investigated, with prediction error calculated as the absolute difference between the actual and predicted values.

### Benchmark tests with ChEMBL dataset

For ChEMBL predictions, most DRP models were trained separately ten times using the entire GDSC1 + 2 as a target label dataset for 100 epochs without data splitting or early stopping. Using these trained models, we predicted 237,580 ln(IC_50_) values for ChEMBL data, covering 112,942 drugs and 404 cell lines during training. Each model utilized cell-line input data from different sources and omics types (Supplementary Table 3). Notably, PaccMann, trained with TPM data from 457 GDSC cell lines, was excluded as it could predict only 60,539 ln(IC_50_) values for these cell lines. DRPreter models with similarity augmentation modules (DRPreter_SA, DRPreter_SA_SG) were also excluded as they cannot predict responses for drugs not encountered during training.

For RNA-based DRP model predictions, we evaluated batch correction by using input gene expression data from 404 cell lines obtained from three sources: (1) TPM data from SANGER Cell Model Passports (v.2.9.0), (2) TPM data from CCLE (DepMap 23Q2; DepMap 21Q4 for DRPreter and DRPreter_SG), (3) Microarray data from GDSC (Release 8.4). For each model, two of the three sources were external datasets not used during training. In the case of TGDRP and TGSA, which incorporate multi-omics data, only RNA data were replaced, while mutation and copy number variation data remained unchanged. tCNNS and GraphDRP models were excluded as they do not utilize RNA expression data. Performance was summarized according to gene expression data sources using the same metrics and statistical methods detailed in Methods (“Benchmark tests with GDSC datasets”). The preparation of gene expression data and batch correction analysis is described in Supplementary Note 4.

### Benchmark tests with TCGA dataset

The prediction was conducted using DRP models trained as outlined in Methods (“Benchmark tests with ChEMBL dataset”). For each drug, the mean predicted responses for responders and non-responders were calculated, and their differences were defined as the effect size. Statistical significance was then evaluated using a one-tailed Mann-Whitney test via the wilcox.test function in the stats R package (v.4.5.0) with alternative = “less”, testing if predicted responses for responders were significantly lower than those for non-responders. For 13 patients containing both tumor and matched-normal samples, predicted responses from tumors were subtracted from those of matched-normal ones. The resulting differences for responders and non-responders were compared using the same wilcox.test function and parameter settings. For TGDRP and TGDRP_SG, only 363 samples having gene expression, mutation and copy number variation (CNV) data were utilized in prediction. For each DRP model, gene expression data were batch-corrected using reference ComBat^32^, implemented via the ComBat function in the sva R package (v3.56.0), with cell-line gene expression data used as reference and cancer tissue type as the matching variable. Benchmark tests were conducted before and after ComBat adjustment.

### Validation of GCNPath with colorectal and breast cancer cell lines

GCNPath models trained as described in Methods (“Benchmark tests with ChEMBL dataset”) were used for prediction. Colorectal and breast cancer cell lines were selected from SANGER Cell Model Passports using GDSC annotations. The cell-line subtype annotation for breast cancer was retrieved from Table 1 of Dai, Xiaofeng, et al. ^34^, whereas the subtype information for colorectal cancer was not available. Eight approved therapies were evaluated, and drug structures (CIDs and SMILES) were retrieved from the PubChem database. Drug targets were identified using annotation files from GDSC and DrugBank. Mutation profiles of BRAF V600E for colorectal cancer and BRCA1/2 at the coding regions for breast cancer were retrieved in SANGER Cell Model Passports (the “Mutations All” category).

### Case study with SCLC

The prediction was conducted using GCNPath, DRPreter_SG, and TGDRP_SG models trained as outlined in Methods (“Benchmark tests with ChEMBL dataset”). For cell-line-based analysis, cell lines, six approved or emerging therapies and their target information were identified or processed following the same steps in Method (“Validation of GCNPath with colorectal and breast cancer cell lines”). For clinical analysis, predictions were made on 92 out of 107 patients whose tumor samples were classified as NMF1, NMF3 and NMF4, and the differences between tumor and matched-normal predicted responses were calculated for each drug and model. Mutation and CNV data from matched-normal samples were not available for TGDRP and TGDRP_SG and were therefore imputed with zeros to preserve feature alignment. Gene expression data from all 107 patients were batch-corrected using reference ComBat^32^ (*sva* R package, v.3.56.0), with 72 SCLC cell lines from SANGER Cell Model Passports used as reference and cancer/normal status as the matching variable. Benchmark tests were conducted before and after ComBat adjustment for comparison.

### Statistics and reproducibility

All performance analyses and visualizations were conducted using R (v.4.5.0) with the caret (v.7.0.1), ggplot2 (v.3.5.1), ggpubr (v.0.6.0), ggrastr (v.1.0.2), and pheatmap (v.1.0.12) packages. For benchmark testing on GDSC datasets, each model was trained using 10- or 25-fold outer cross-validation, yielding 10 or 25 performance replicates per dataset and test type. For other benchmark tests, models were trained with 10 different random seeds to generate 10 performance replicates. Model performance comparisons were conducted using one-tailed Mann–Whitney U tests via the wilcox.test function from the stats package (v4.3.3). Boxplots display interquartile ranges (IQR) with whiskers extending to 1.5×IQR, alongside individual data points. Bar plots depict mean ± standard deviation (SD) with individual data points overlaid.

### Reporting summary

Further information on research design is available in the Nature Portfolio Reporting Summary linked to this article.

## Supplementary information

Supplementary Information

Reporting Summary

## Data Availability

Cell-line TPM data were obtained from the SANGER Cell Model Passports database (version 2.9.0; https://cog.sanger.ac.uk/cmp/download/rnaseq_all_20220624.zip; “rnaseq_tpm_20220624.csv”) and the CCLE DepMap database (versions 21Q4 and 23Q2; https://depmap.org/portal/data_page/?tab=allData&releasename=DepMap%20Public%2021Q4&filename=CCLE_expression_full.csv; https://depmap.org/portal/download/all/?releasename=DepMap+Public+23Q2&filename=OmicsExpressionProteinCodingGenesTPMLogp1.csv). Cell-line microarray data were retrieved from GDSC Release 8.4 (https://www.cancerrxgene.org/gdsc1000/GDSC1000_WebResources/Data/preprocessed/Cell_line_RMA_proc_basalExp.txt.zip). Protein–protein interaction (PPI) and gene regulatory network (GRN) data were obtained from STRING (version 11.5; https://string-db.org/cgi/download?sessionId=btooBqgiAEcP&species_text=9606) and RegNetwork (https://regnetworkweb.org/download/human.zip; “human.source”; https://regnetworkweb.org/download/RegulatoryDirections.zip; “new_kegg.human.reg.direction.txt”), respectively. BIOCARTA pathway information was downloaded from MSigDB (version 2023.1.Hs; https://www.gsea-msigdb.org/gsea/msigdb/download_file.jsp?filePath=/msigdb/release/2023.1.Hs/msigdb_v2023.1.Hs_files_to_download_locally.zip; “c2.cp.biocarta.v2023.1.Hs.entrez.gmt”, “c2.cp.biocarta.v2023.1.Hs.symbols.gmt”). The PubChem compound identifiers (CIDs) of drugs from GDSC were retrieved using the PubChem Identifier Exchange Service (https://pubchem.ncbi.nlm.nih.gov/idexchange/idexchange.cgi) and the webchem R package (version 1.2.0). Corresponding SMILES representations were obtained from PubChem (https://pubchem.ncbi.nlm.nih.gov). Drug sensitivity data, along with cell-line and drug annotation information, were sourced from GDSC Release 8.4 (https://www.cancerrxgene.org/downloads/drug_data?screening_set=GDSC1; https://www.cancerrxgene.org/downloads/drug_data?screening_set=GDSC2; https://www.cancerrxgene.org/celllines; https://www.cancerrxgene.org/compounds). Drug sensitivity data from ChEMBL Release 33 were downloaded using the ChEMBL web resource client Python package (version 0.10.8). TCGA clinical and TPM data (version 37.0), along with standardized drug names, were obtained using the TCGABiolinks R package (version 2.25.3) and GDISC (https://gdisc.bme.gatech.edu/Data/DrugCorrection.csv; https://gdisc.bme.gatech.edu/Data/GoodlookingTable.csv), respectively. SCLC clinical data and TPM data were collected from Liu, Qian, et al. ^36^ via their supplementary tables (Table S1 and S6A), respectively. All raw data used in this study are publicly available. The preprocessing procedures are fully specified and reproducible, as described in the GitHub repository (https://github.com/MinhoLee-DGU/GCNPath2026), specifically within the *IC50_Prediction/project* subdirectory. Numerical data underlying the figures are provided as Supplementary Data 1–54 in the Zenodo repository (DOI: 10.5281/zenodo.18924547), as cited in the manuscript and Supplementary Information. Numerical data cited in the manuscript but not underlying the figures are also available as Supplementary Data files within the same repository (Supplementary Data 43–45, 47–49, and 51–54).

## Code availability

The source code for the GCNPath model and data processing steps are available at https://github.com/MinhoLee-DGU/GCNPath2026.
