# Supplementary material for: GCNPath: introspecting drug response prediction with pathway-guided graph convolution networks
Source: Commun Biol. 2026 Apr 1;9:720. doi: 10.1038/s42003-026-09957-5 (PMC13212977; doi:10.1038/s42003-026-09957-5)
Supplement: Supplementary file 1 — Supplementary Information [file 42003_2026_9957_MOESM1_ESM.pdf]

GCNPath: Introspecting Drug Response Prediction with Pathway-guided Graph Convolution Networks

Hyeon Jun Yoon[1], and Minho Lee[1,*]

[1]Department of Life Science, Dongguk University, Gyeonggi-do, 10326, Republic of Korea

***Corresponding author**:

Minho Lee

Department of Life Science, Dongguk University,

32 Dongguk-ro, Ilsandong-gu, Goyang-si, Gyeonggi-do 10326, Republic of Korea

Phone: +82-32-961-5138

E-mail: MinhoLee@dgu.edu

Keywords: anticancer drug response prediction, deep learning, graph convolution network, interactions between pathways, feature reduction

Supplementary Figures

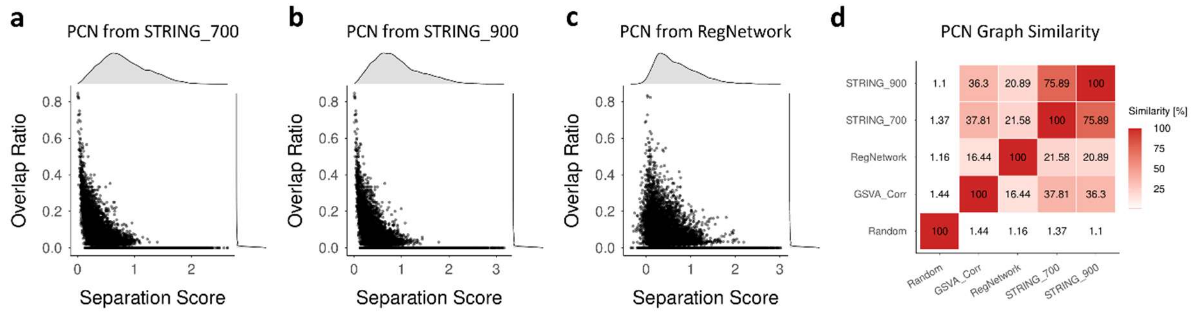

Supplementary Figure 1. Basic topological information of pathway crosstalk network (PCN) graphs. (**a-c**) Scatter plots of the separation scores and overlap ratios of each pathway pair in the STRING database with confidence scores equal to or greater than 0.7 or 0.9 ((**a**) STRING_700, (**b**) STRING_900) and (**c**) RegNetwork. For each PCN graph, 292 pathways from BIOCARTA were utilized. (**d**) Overlap ratios of edges between each PCN graph pair from STRING, RegNetwork, the correlation network of pathway activity scores calculated via gene set variation analysis (GSVA) and the network with randomized edges. Source data are provided in Supplementary Data 1. STRING: PCN graph from STRING with confidence scores equal to or greater than 0.7 or 0.9; RegNetwork: PCN graph from RegNetwork; GSVA_Corr: GSVA correlation graph; Random: PCN graph with randomized edges.

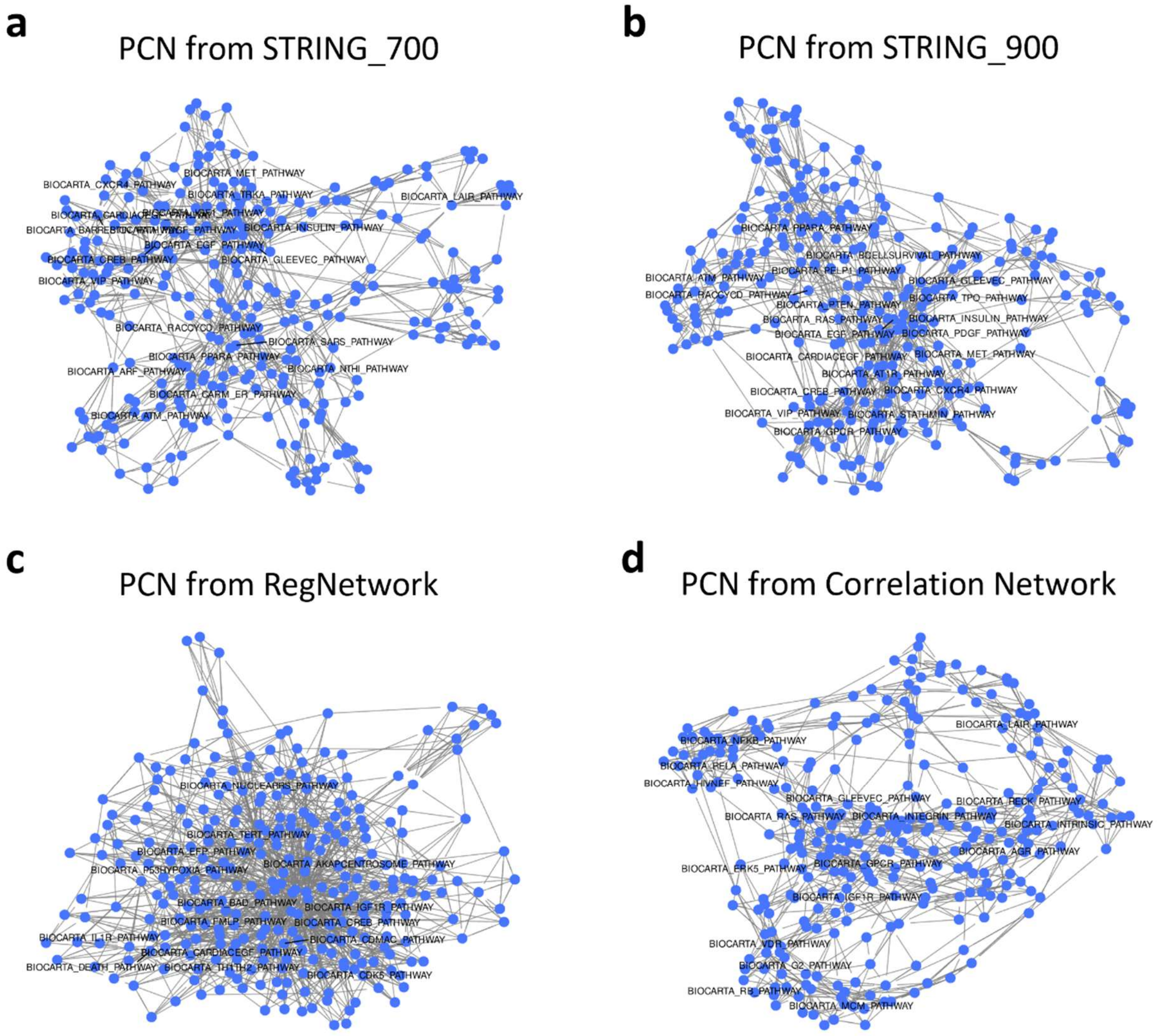

Supplementary Figure 2. Visualization of PCN graphs. PCN graphs compressed from (**a, b**) STRING with confidence scores equal to or greater than 0.7 and 0.9, (**c**) RegNetwork and (**d**) GSVA correlation network. For each PCN graph, 292 BIOCARTA pathways were utilized. The source data are provided in Supplementary Data 2.

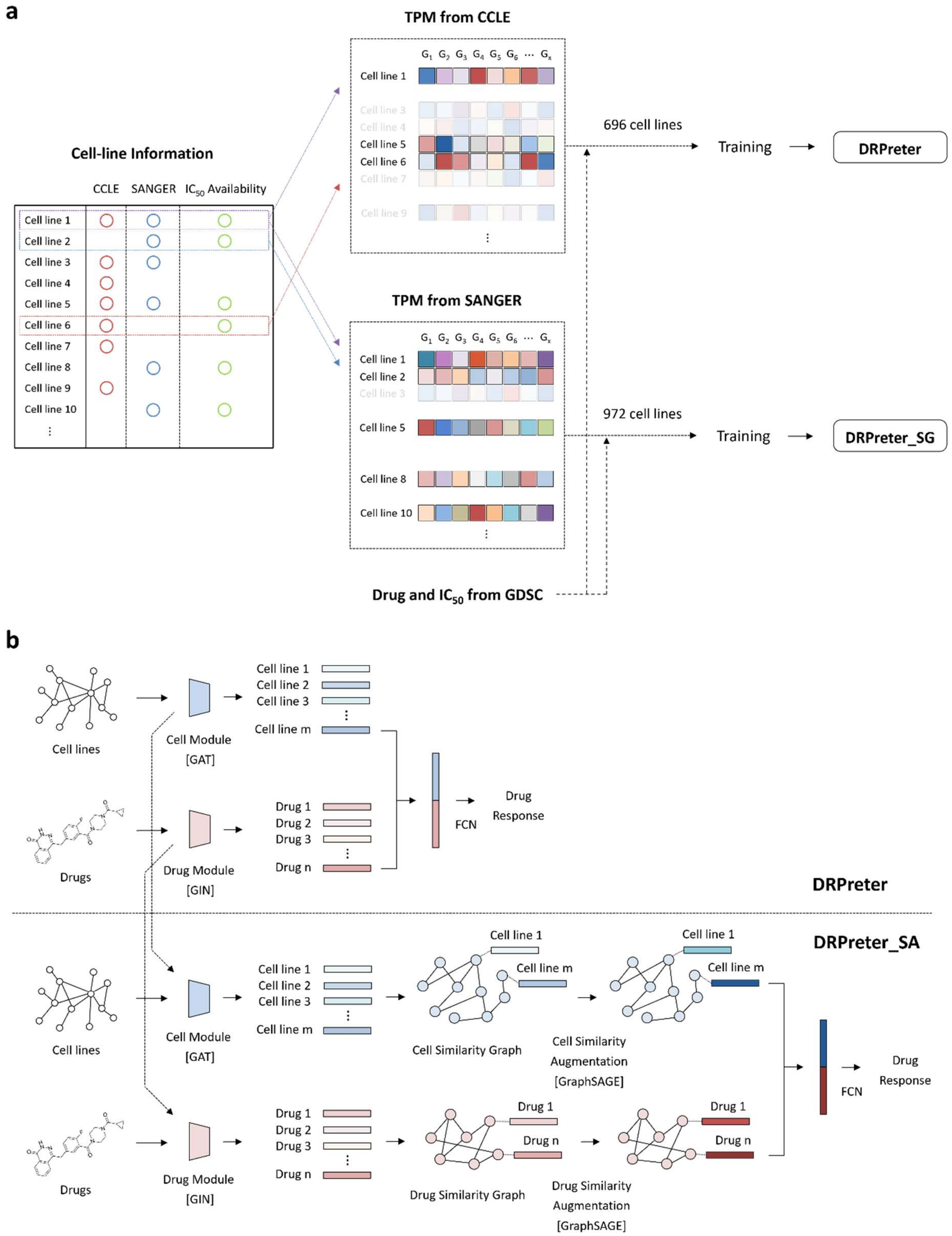

Supplementary Figure 3. Illustration of the model training process. (**a**) Schematic of "model_name_SG". Deep learning models for drug response prediction use cell line omics data from databases like GDSC, CCLE, and SANGER Cell Model Passports. The choice of database affects the amount of training and testing data (**Supplementary Table 3**). For instance, DRPreter was trained using

TPM data from CCLE (DepMap 21Q4 version). Of the 1,389 cell lines from CCLE, only 696 were screened for drug responses in GDSC. In contrast, in SANGER Cell Model Passports (v.2.9.0), 972 out of 1,431 cell lines were available, the same number as our proposed GCNPath model. Training models with SANGER data could address data scarcity and imbalance in deep learning. (**b**) Schematic of "DRPreter_SA". After training DRPreter, one-dimensional embeddings for cell lines and drugs were extracted from their respective modules. Similarity graphs between cell lines and drugs were generated using gene expression and chemical structure data. These graphs and embeddings were processed with graph convolution networks (GCNs) to align similar cell lines and drugs. Drug responses were then predicted based on these embeddings. The similarity augmentation modules were first introduced in TGSA. GAT: graph attention network, GIN: graph isomorphism network, FCN: fully connected network, GraphSAGE: Graph SAmple and aggreGatE network.

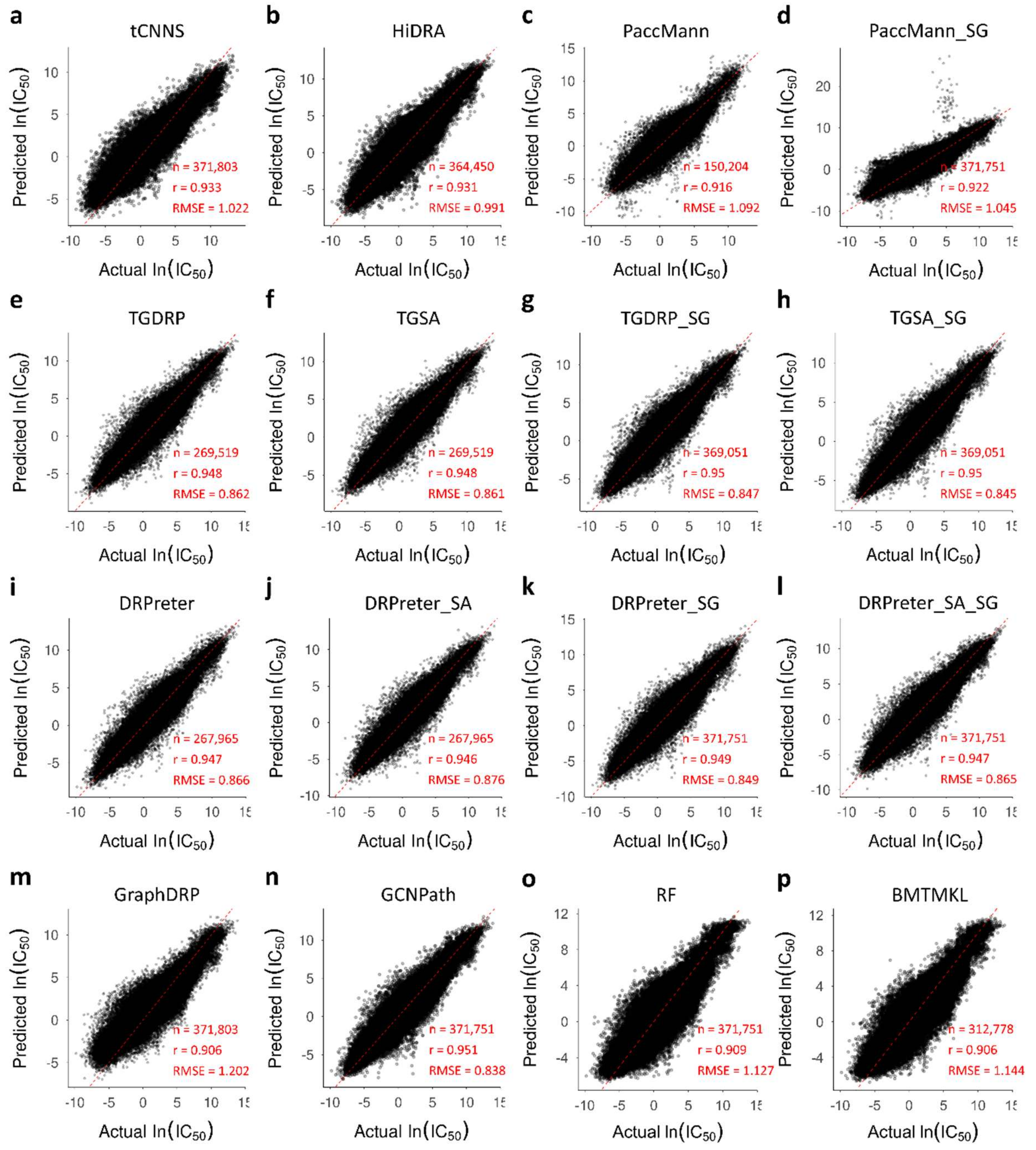

Supplementary Figure 4. Scatter plots of predictions in unblinded tests with GDSC1+2 as a target label dataset. All predicted values for trained models with 10-fold outer cross-validation were visualized. The number of predicted ln(IC_{50}) values (n), prediction PCC (r) and RMSE are indicated in each scatter plot using red text. Unexpectedly, tCNNS output a value of infinity for 37,180 of the 371,803 predicted ln(IC_{50}) values. The source data are provided in Supplementary Data 5. SG: model trained with cell line data from SANGER Cell Model Passports as cell-line input data; DRPreter_SA: DRPreter with a similarity augmentation module.

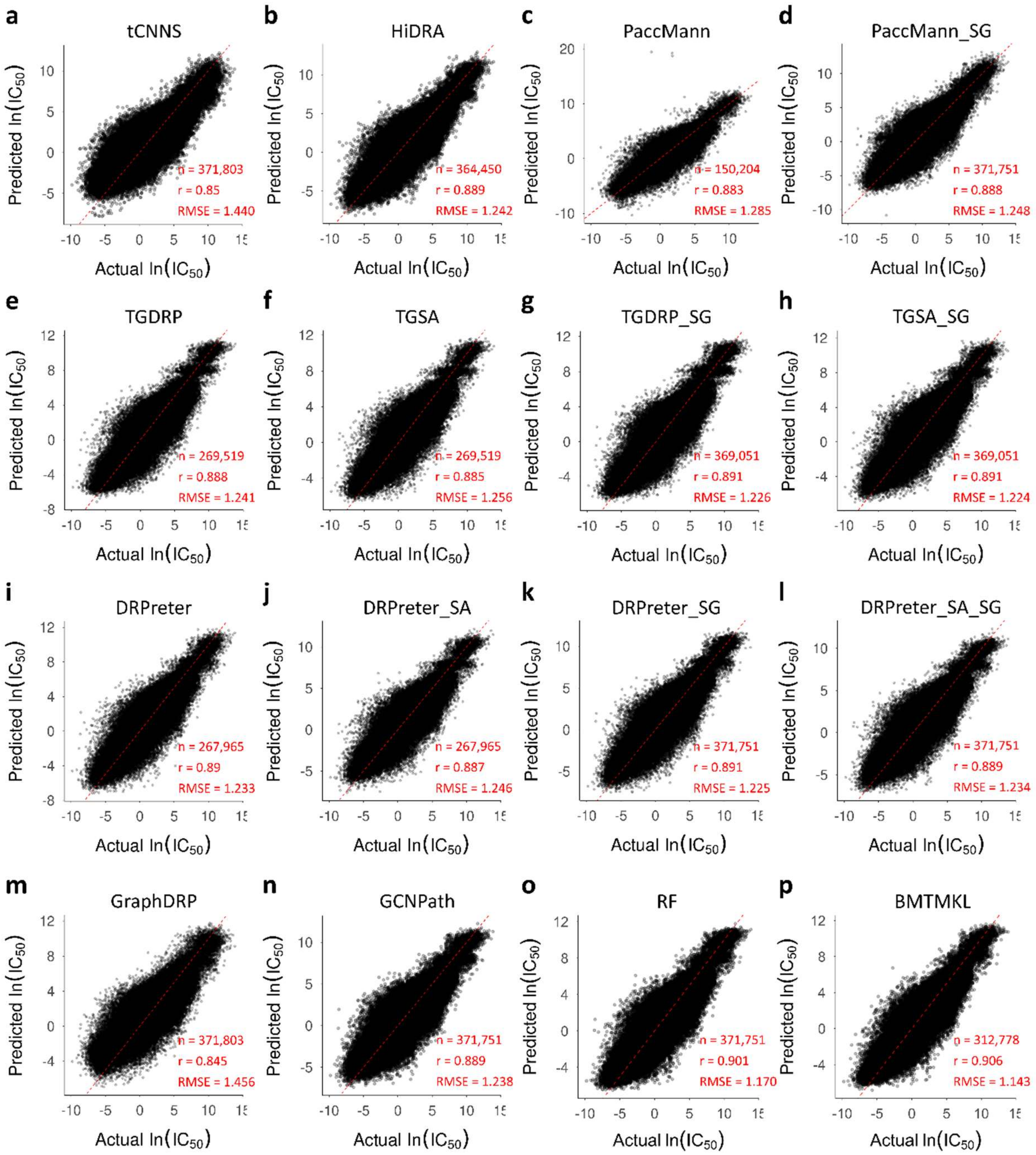

Supplementary Figure 5. Scatter plots of predictions in cell-blind tests with GDSC1+2 as a target label dataset. All predicted values for trained models with 10-fold outer cross-validation were visualized. The number of predicted ln(IC_{50}) values (n), prediction PCC (r) and RMSE are indicated in each scatter plot using red text. The source data are provided in Supplementary Data 6. SG: model trained with cell line data from SANGER Cell Model Passports as cell-line input data; DRPreter_SA: DRPreter with a similarity augmentation module.

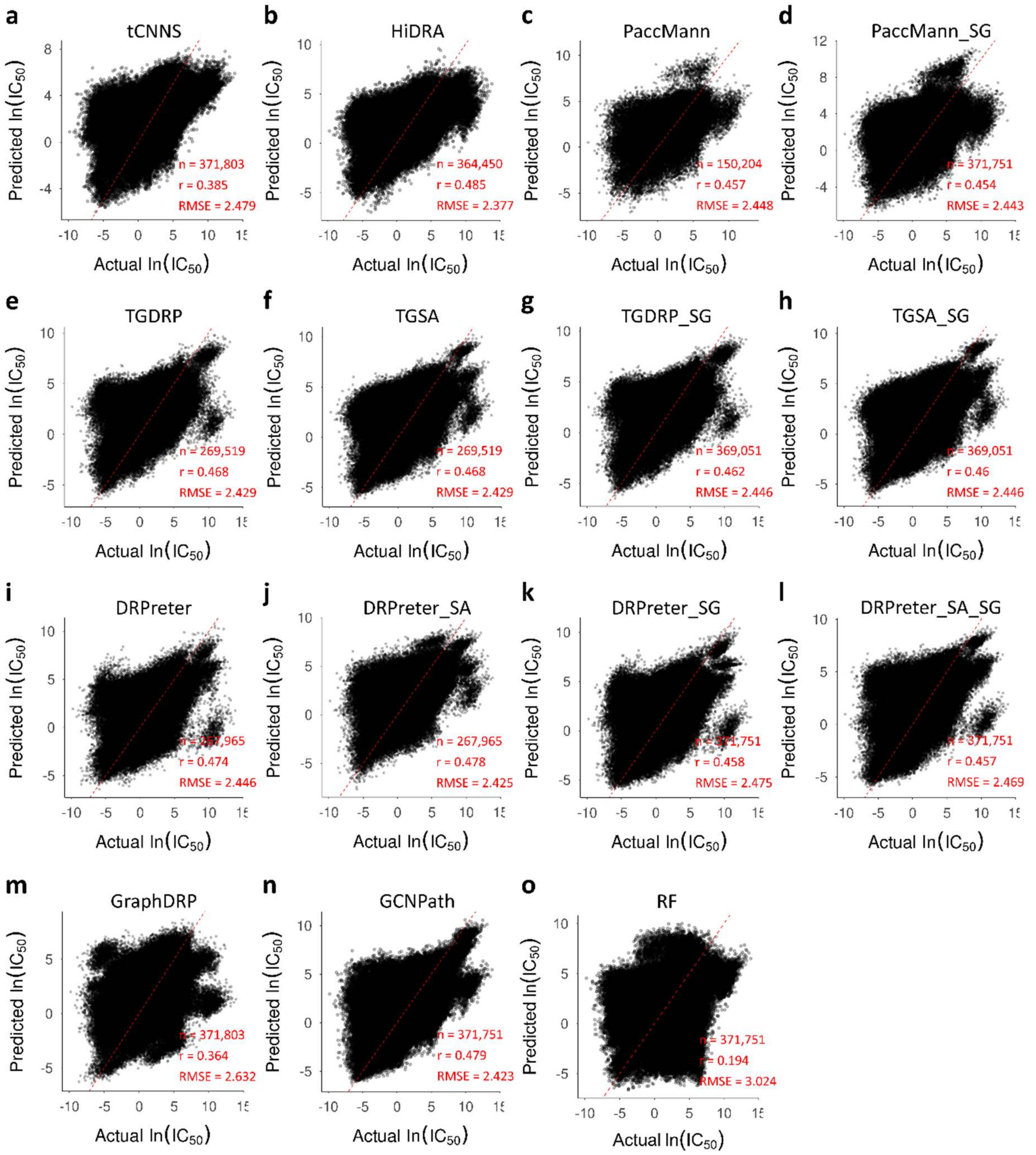

Supplementary Figure 6. Scatter plots of predictions in drug-blind tests with GDSC1+2 as a target label dataset. All predicted values for trained models with 10-fold outer cross-validation were visualized. The number of predicted ln(IC_{50}) values (n), prediction PCC (r) and RMSE are indicated in each scatter plot using red text. tCNNS output a value of infinity for 36,970 of the 371,803 predicted ln(IC_{50}) values. The source data are provided in Supplementary Data 7. SG: model trained with cell line data from SANGER Cell Model Passports as cell-line input data; DRPreter_SA: DRPreter with a similarity augmentation module.

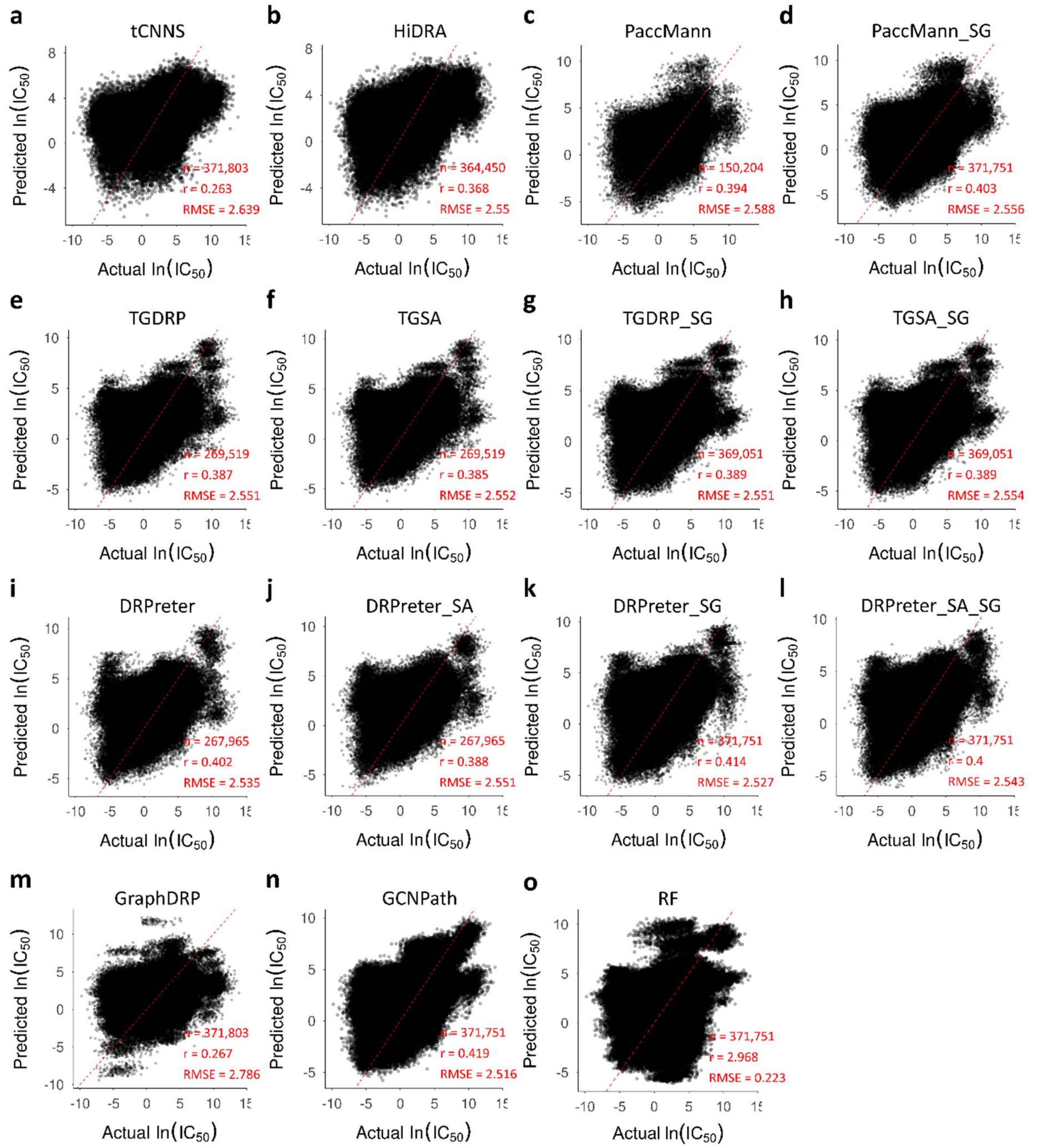

Supplementary Figure 7. Scatter plots of predictions in strict-blind tests with GDSC1+2 as a target label dataset. All predicted values for trained models with 10-fold outer cross-validation were visualized. The number of predicted ln(IC_{50}) values (n), prediction PCC (r) and RMSE are indicated in each scatter plot using red text. Unexpectedly, tCNNS output a value of infinity for 15,086 of 371,803 predicted ln(IC_{50}) values. The source data are provided in Supplementary Data 8. SG: model trained with cell line data from SANGER Cell Model Passports as cell-line input data; DRPreter_SA: DRPreter with a similarity augmentation module.

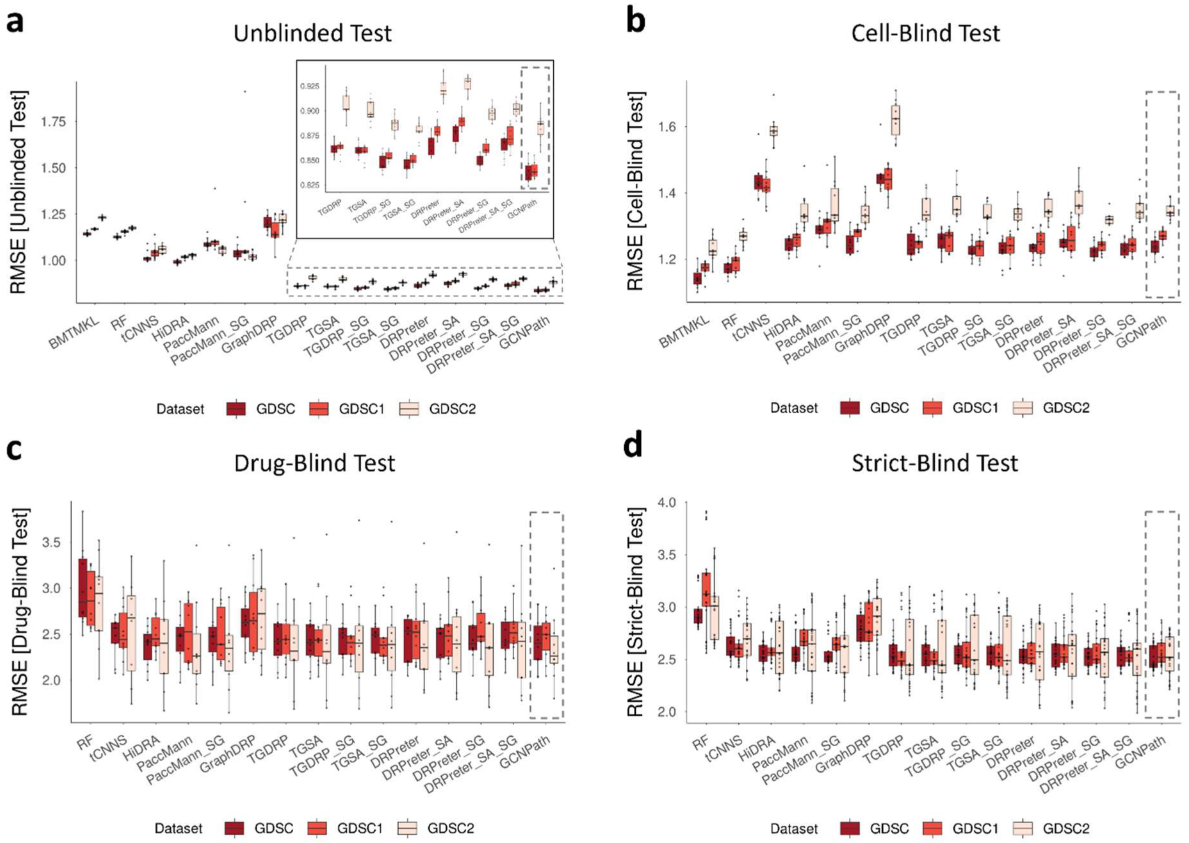

Supplementary Figure 8. RMSEs of different models from testing with individual GDSC as target label datasets. Each model was trained and tested with GDSC as target label datasets, employing 10-fold outer cross-validation in (**a**) unblinded, (**b**) cell-blind and (**c**) drug-blind tests and 25-fold outer cross-validation in (**d**) strict-blind tests. This procedure produced (**a-c**) 10 RMSEs and (**d**) 25 RMSEs per box (n=10 or 25 in each box). The performances of GCNPath are shown within dotted square boxes across all box plots. Outlier (infinity) values produced in a small number of tCNNS calculations were excluded from the calculations (**Supplementary Table 4**). Boxplots display the interquartile range (IQR), with whiskers extending 1.5×IQR. The source data are provided in Supplementary Data 9. SG: model trained with cell line data from SANGER Cell Model Passports as cell-line input data. DRPreter_SA: DRPreter with a similarity augmentation module.

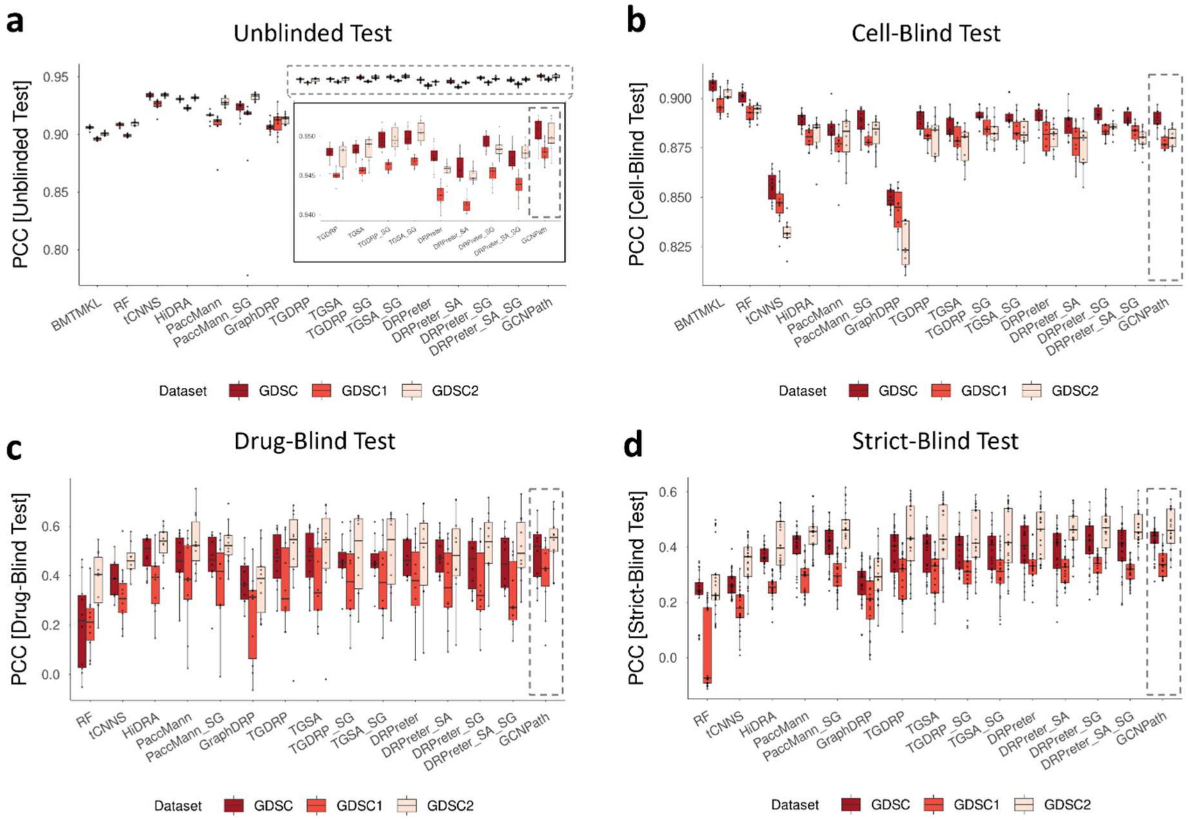

Supplementary Figure 9. PCCs of different models from testing with individual GDSC as target label datasets. Each model was trained and tested with GDSC as target label datasets, employing 10-fold outer cross-validation in (**a**) unblinded, (**b**) cell-blind and (**c**) drug-blind tests and 25-fold outer cross-validation in (**d**) strict-blind tests. This procedure produced (**a-c**) 10 PCCs and (**d**) 25 PCCs per box (n=10 or 25 in each box). The performances of GCNPath are shown within dotted square boxes across all box plots. Outlier (infinity) values produced in a small number of tCNNS calculations were excluded from the calculations (**Supplementary Table 5**). Boxplots display the IQR, with whiskers extending 1.5×IQR. The source data are provided in Supplementary Data 10. SG: model trained with cell line data from SANGER Cell Model Passports as cell-line input data; DRPreter_SA: DRPreter with a similarity augmentation module.

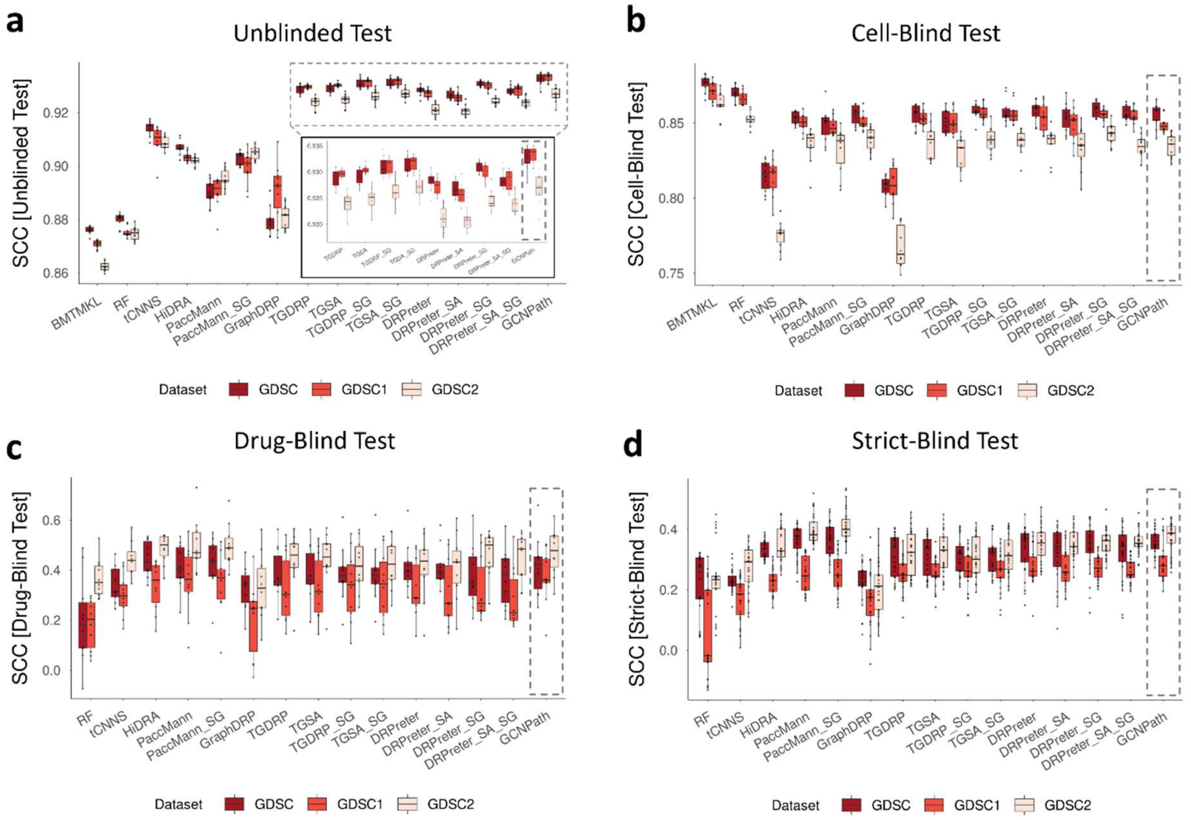

Supplementary Figure 10. SCCs of different models from testing with individual GDSC as target label datasets. Each model was trained and tested with GDSC as target label datasets, employing 10-fold outer cross-validation in (**a**) unblinded, (**b**) cell-blind and (**c**) drug-blind tests and 25-fold outer cross-validation in (**d**) strict-blind tests. This procedure produced (**a-c**) 10 SCCs and (**d**) 25 SCCs per box (n=10 or 25 in each box). The performances of GCNPath are shown within dotted square boxes across all box plots. Outlier (infinity) values produced in a small number of tCNNS calculations were excluded from the calculations (**Supplementary Table 6**). Boxplots display the IQR, with whiskers extending 1.5×IQR. The source data are provided in Supplementary Data 11. SG: model trained with cell line data from SANGER Cell Model Passports as cell-line input data; DRPreter_SA: DRPreter with a similarity augmentation module.

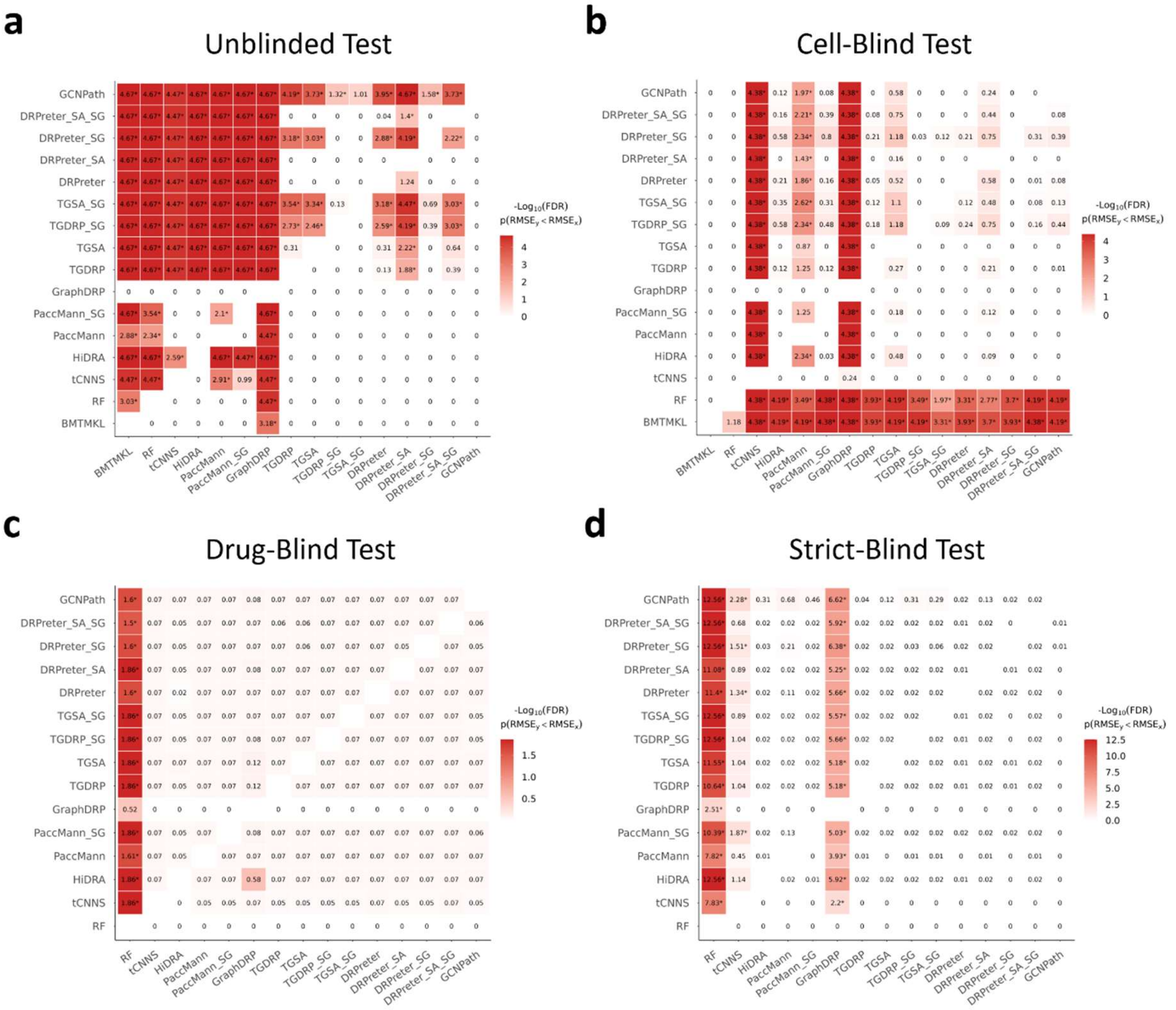

Supplementary Figure 11. U tests of the RMSEs between models trained with the GDSC1+2 as a target label dataset. Each model was trained and tested with GDSC1+2 as a target label dataset, employing 10-fold outer cross-validation for the unblinded, cell-blind, and drug-blind tests and 25-fold outer cross-validation for the strict-blind tests. Consequently, for the strict-blind tests, 25 RMSEs were calculated for each model, and for the other test types, 10 RMSEs were calculated for each model. Then, U tests were performed to compare the calculated RMSEs from the (**a**) unblinded, (**b**) cell-blind, (**c**) drug-blind and (**d**) strict-blind tests. The $-\log_{10}$-scaled FDR indicates whether the model on the y-axis has a significantly lower RMSE than that on the x-axis. FDR values were obtained from the one-tailed Mann–Whitney test. The source data are provided in Supplementary Data 12. *: FDR ≤ 0.05.

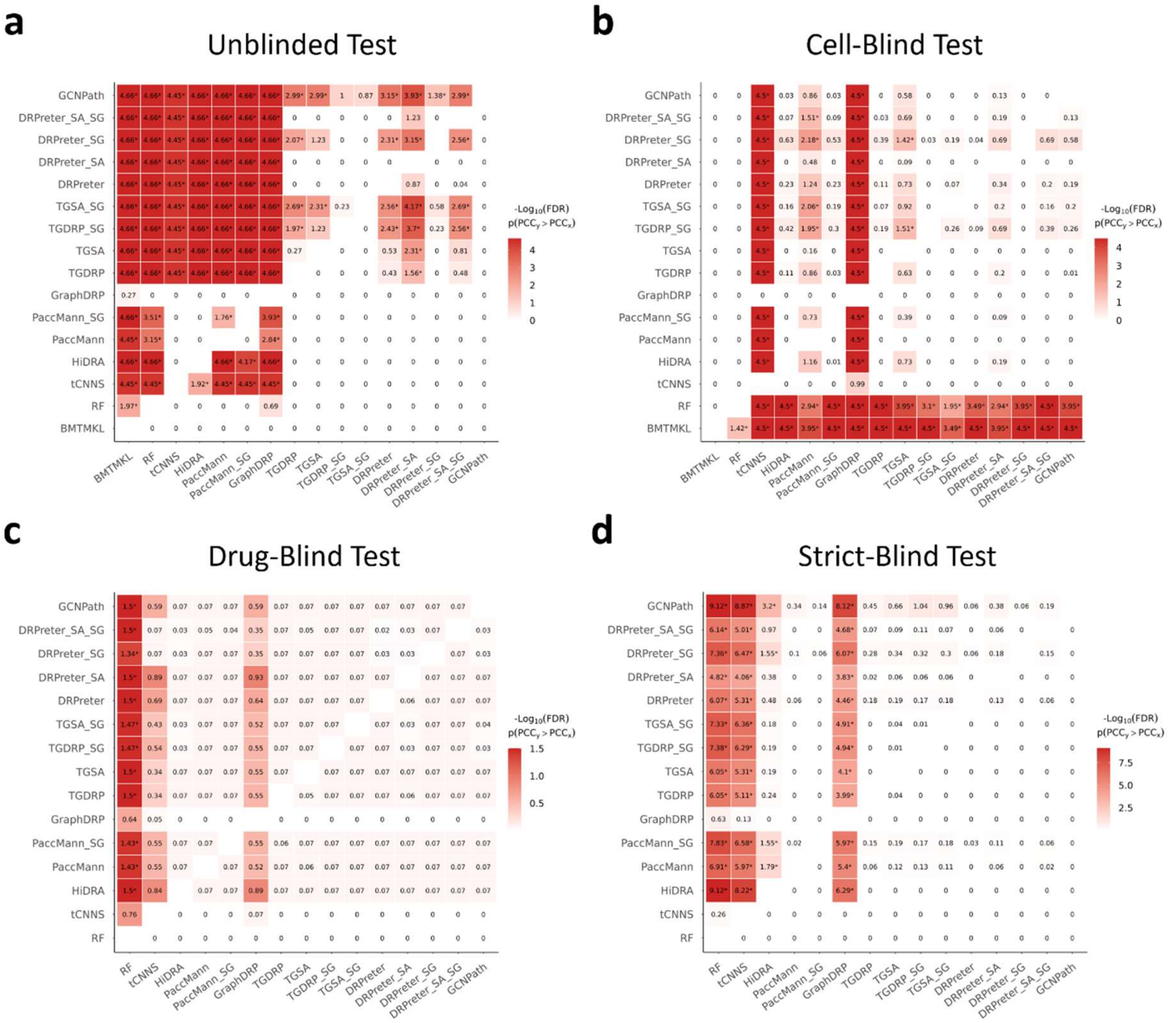

Supplementary Figure 12. U tests of PCCs between models trained with the GDSC1+2 as a target label dataset. Each model was trained and tested with GDSC1+2 as a target label dataset, employing 10-fold outer cross-validation for the unblinded, cell-blind, and drug-blind tests and 25-fold outer cross-validation for the strict-blind tests. Consequently, for the strict-blind tests, 25 PCCs were calculated for each model, and for the other test types, 10 PCCs were calculated for each model. U tests were used to compare the PCCs calculated in the (**a**) unblinded, (**b**) cell-blind, (**c**) drug-blind and (**d**) strict-blind tests. The $-\log_{10}$-scaled FDR indicates whether the model on the y-axis has a significantly greater PCCs than that on the x-axis. FDR values were obtained from the one-tailed Mann–Whitney test. The source data are provided in Supplementary Data 13. *: FDR ≤ 0.05.

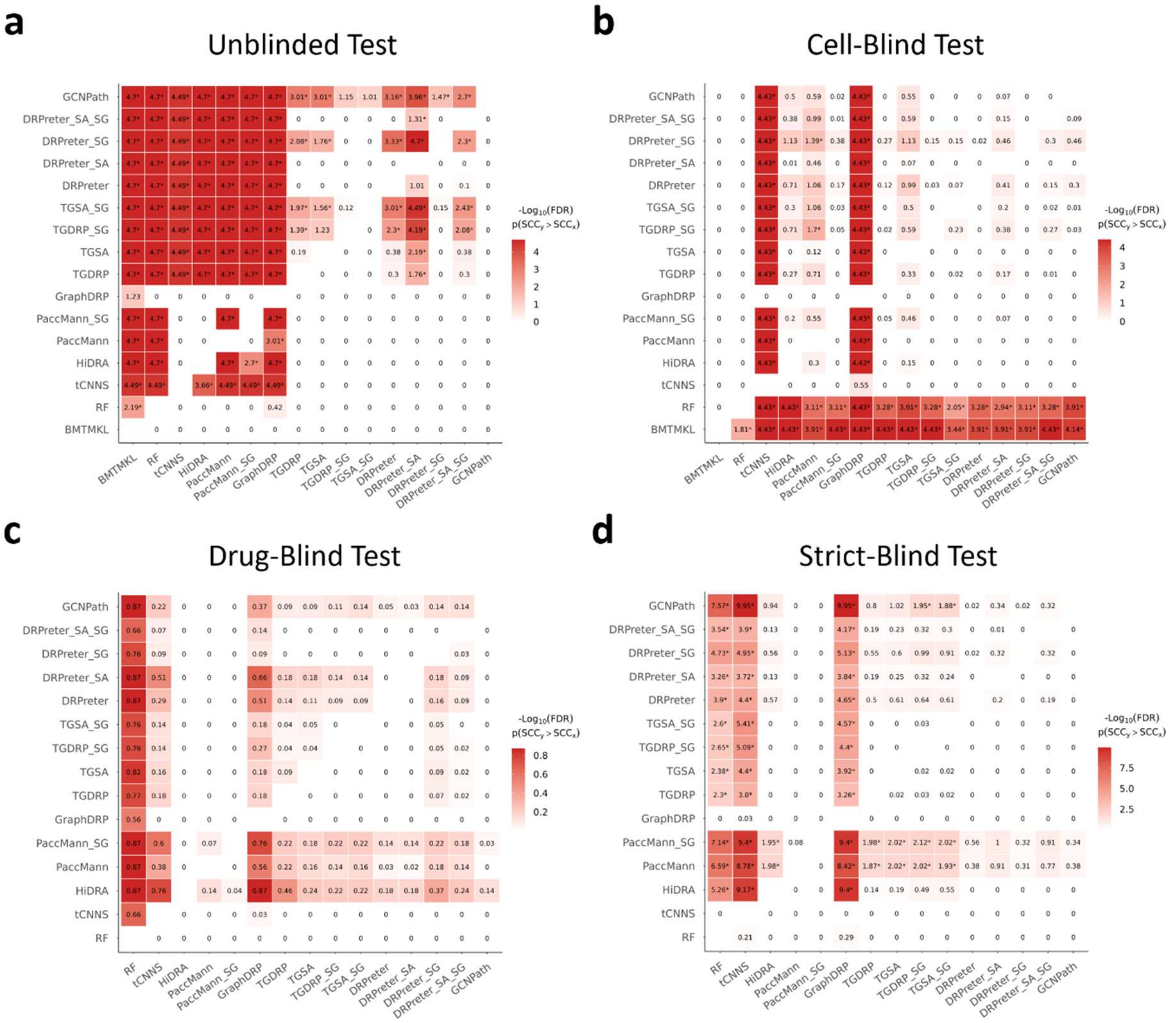

Supplementary Figure 13. U tests of SCCs between models trained with the GDSC1+2 as a target label dataset. Each model was trained and tested with GDSC1+2 as a target label dataset, employing 10-fold outer cross-validation for the unblinded, cell-blind, and drug-blind tests and 25-fold outer cross-validation for the strict-blind tests. Consequently, for the strict-blind tests, 25 SCCs were calculated for each model, and for the other test types, 10 SCCs were calculated for each model. U tests were used to compare the SCCs calculated in the (**a**) unblinded, (**b**) cell-blind, (**c**) drug-blind and (**d**) strict-blind tests. The $-\log_{10}$-scaled FDR indicates whether the model on the y-axis has a significantly greater SCC than that on the x-axis. FDR values were obtained from the one-tailed Mann–Whitney test. The source data are provided in Supplementary Data 14. *: FDR ≤ 0.05.

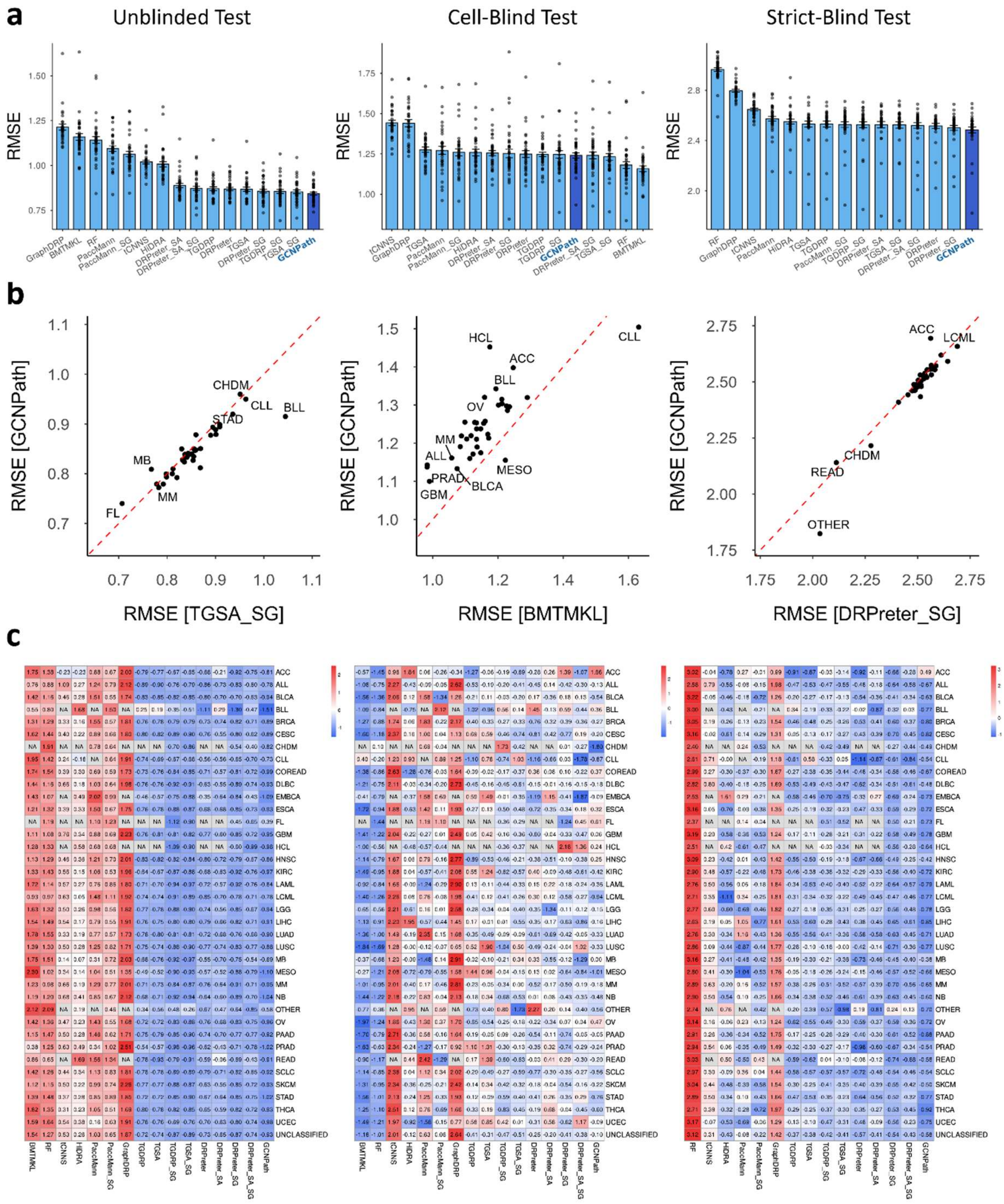

Supplementary Figure 14. RMSEs of various models tested using GDSC1+2 as the target label datasets, stratified by cell tissue types. Each model was trained and evaluated using GDSC as the target label dataset, with 10-fold outer cross-validation applied in the (left) unblinded and (middle) cell-blind settings, and 25-fold outer cross-validation used in the (right) strict-blind test. RMSE values were computed for 38 cell tissue types, as annotated in the GDSC database. The results are presented as (**a**) bar plots, (**b**) scatter plots comparing GCNPath to the first- or second-best performing model, and

(**c**) heatmaps showing z-normalized RMSE values across tissue types (row-wise normalization). In (**a**), RMSEs are reported as mean ± standard deviation, represented by error bars. The source data are provided in Supplementary Data 15. SG: model trained with cell line data from SANGER Cell Model Passports as cell-line input data. DRPreter_SA: DRPreter with a similarity augmentation module.

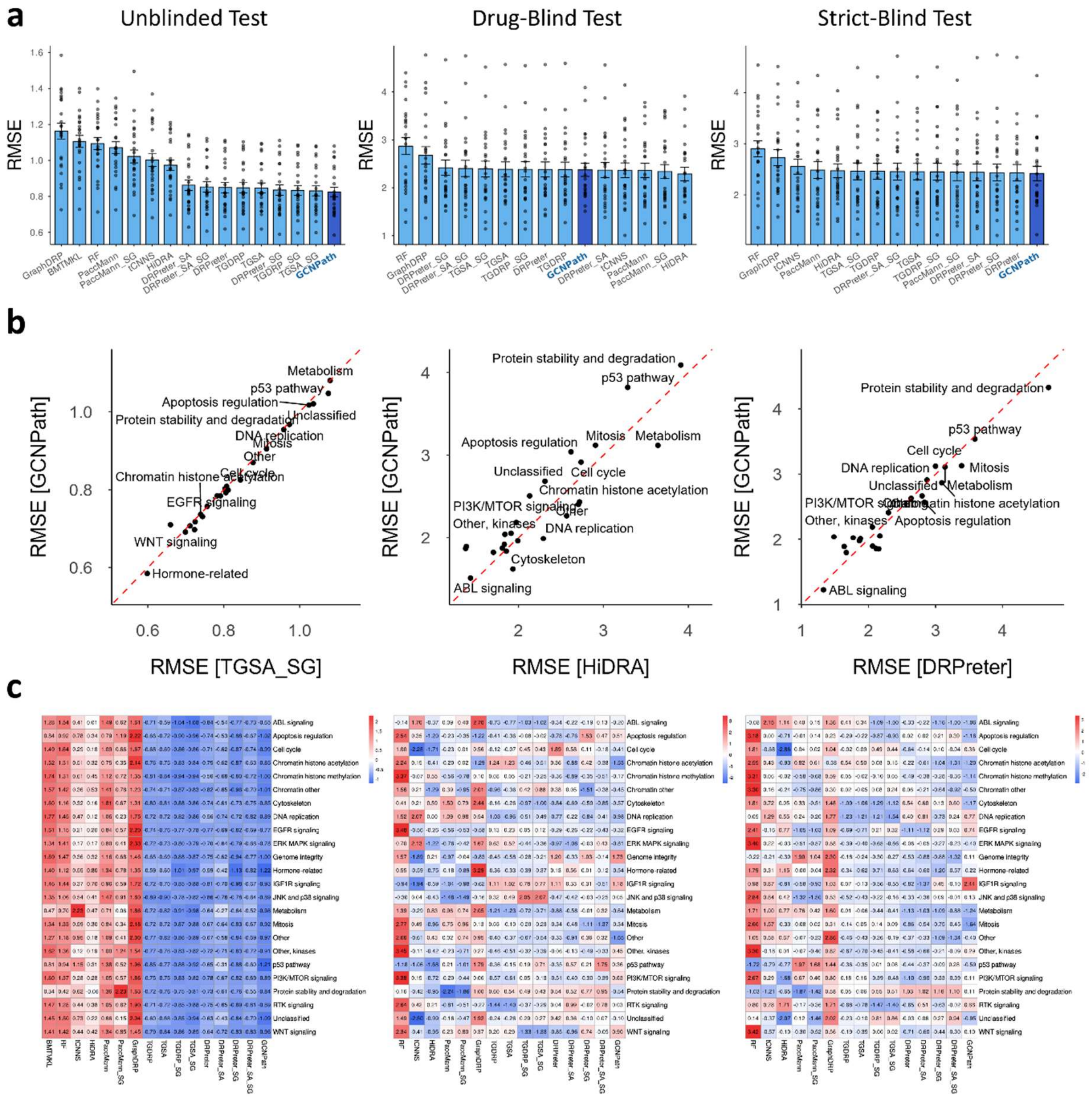

Supplementary Figure 15. RMSEs of various models tested using GDSC1+2 as the target label datasets, stratified by drug target pathways. Each model was trained and evaluated using GDSC as the target label dataset, with 10-fold outer cross-validation applied in the (left) unblinded and (middle) cell-blind settings, and 25-fold outer cross-validation used in the (right) strict-blind test. RMSE values were computed for 24 drug target pathways, as annotated in the GDSC database. The results are presented as (**a**) bar plots, (**b**) scatter plots comparing GCNPath to the first- or second-best performing model, and (**c**) heatmaps showing z-normalized RMSE values across tissue types (row-wise normalization). In (**a**), RMSEs are reported as mean ± standard deviation, represented by error bars. The source data are provided in Supplementary Data 16. SG: model trained with cell line data from

SANGER Cell Model Passports as cell-line input data. DRPreter_SA: DRPreter with a similarity augmentation module.

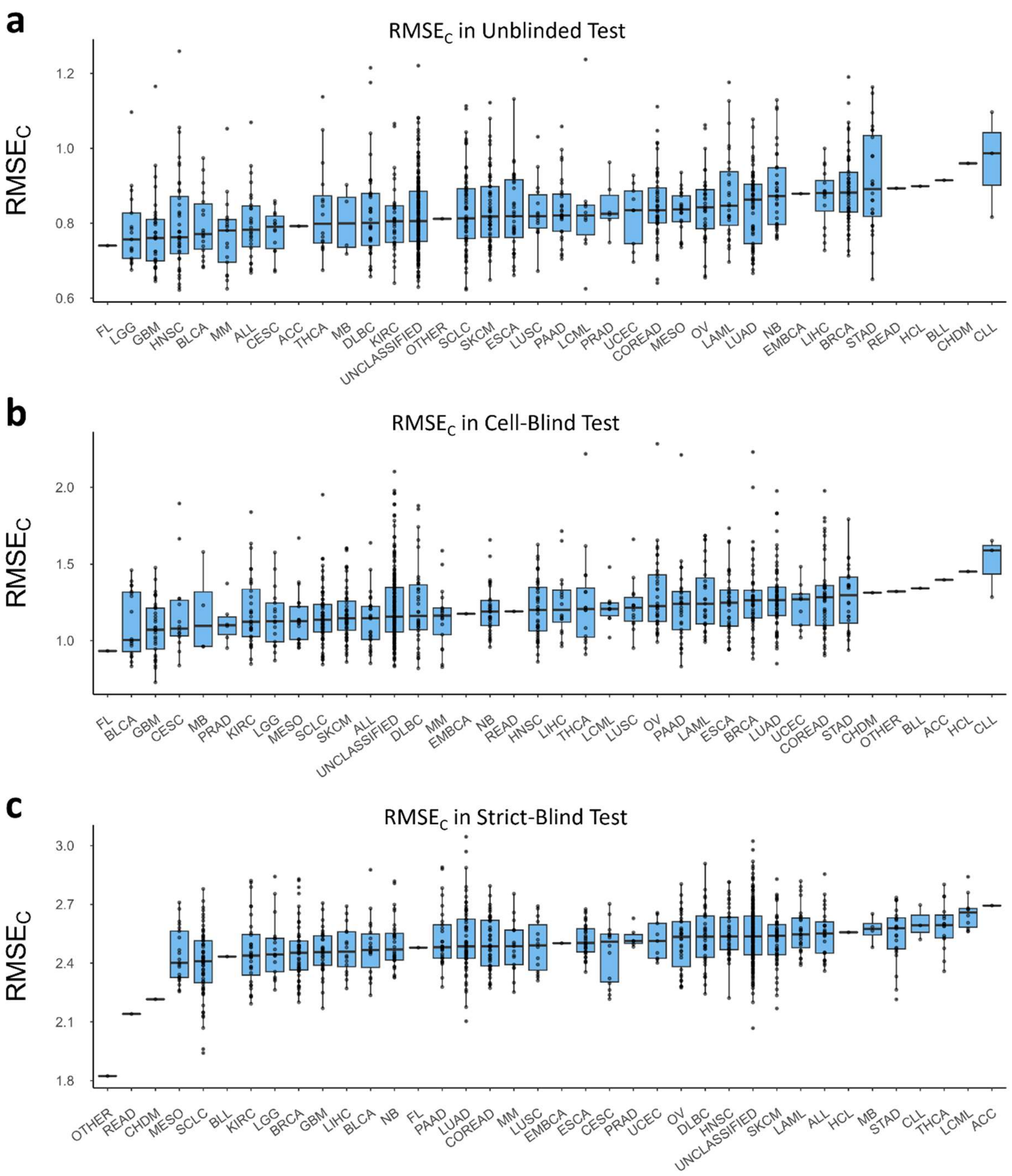

Supplementary Figure 16. RMSE values for each cell line (RMSE$_C$) in GCNPath tested using GDSC1+2 as the target label datasets, stratified by cell tissue types. GCNPath was trained and evaluated using GDSC as the target label dataset, with 10-fold outer cross-validation applied in the (**a**) unblinded and (**b**) cell-blind settings, and 25-fold outer cross-validation used in the (**c**) strict-blind test. RMSE$_C$ values were computed, and the median value for each of the 38 tissue types as annotated in the GDSC database was sorted in ascending order for visualization. The source data are provided in Supplementary Data 17.

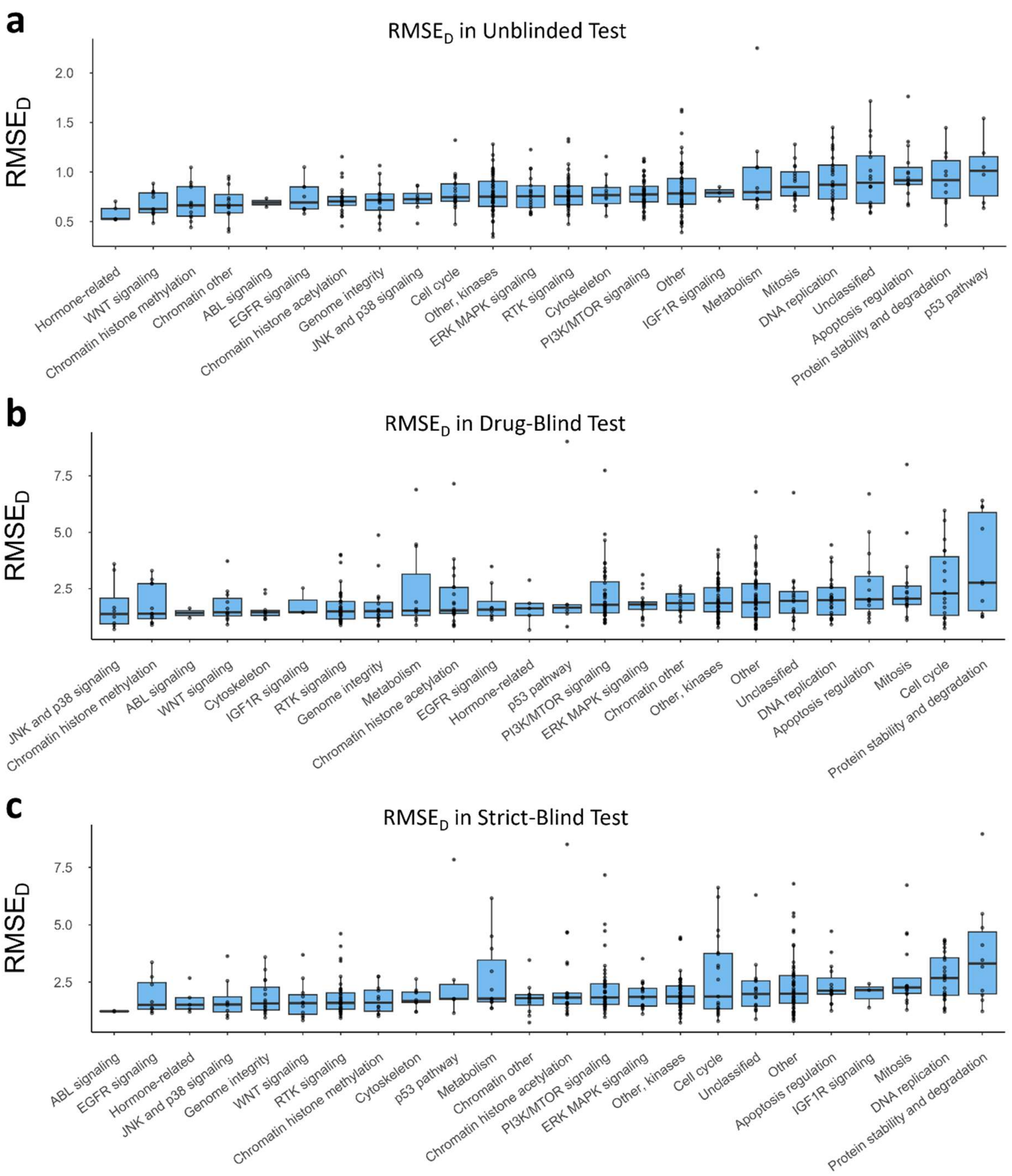

Supplementary Figure 17. RMSE values for each drug ($RMSE_D$) in GCNPath tested using GDSC1+2 as the target label datasets, stratified by cell tissue types. GCNPath was trained and evaluated using GDSC as the target label dataset, with 10-fold outer cross-validation applied in the (**a**) unblinded and (**b**) cell-blind settings, and 25-fold outer cross-validation used in the (**c**) strict-blind test. $RMSE_D$ values were computed, and the median value for each of the 24 target pathways as annotated in the GDSC database was sorted in ascending order for visualization. The source data are provided in Supplementary Data 18.

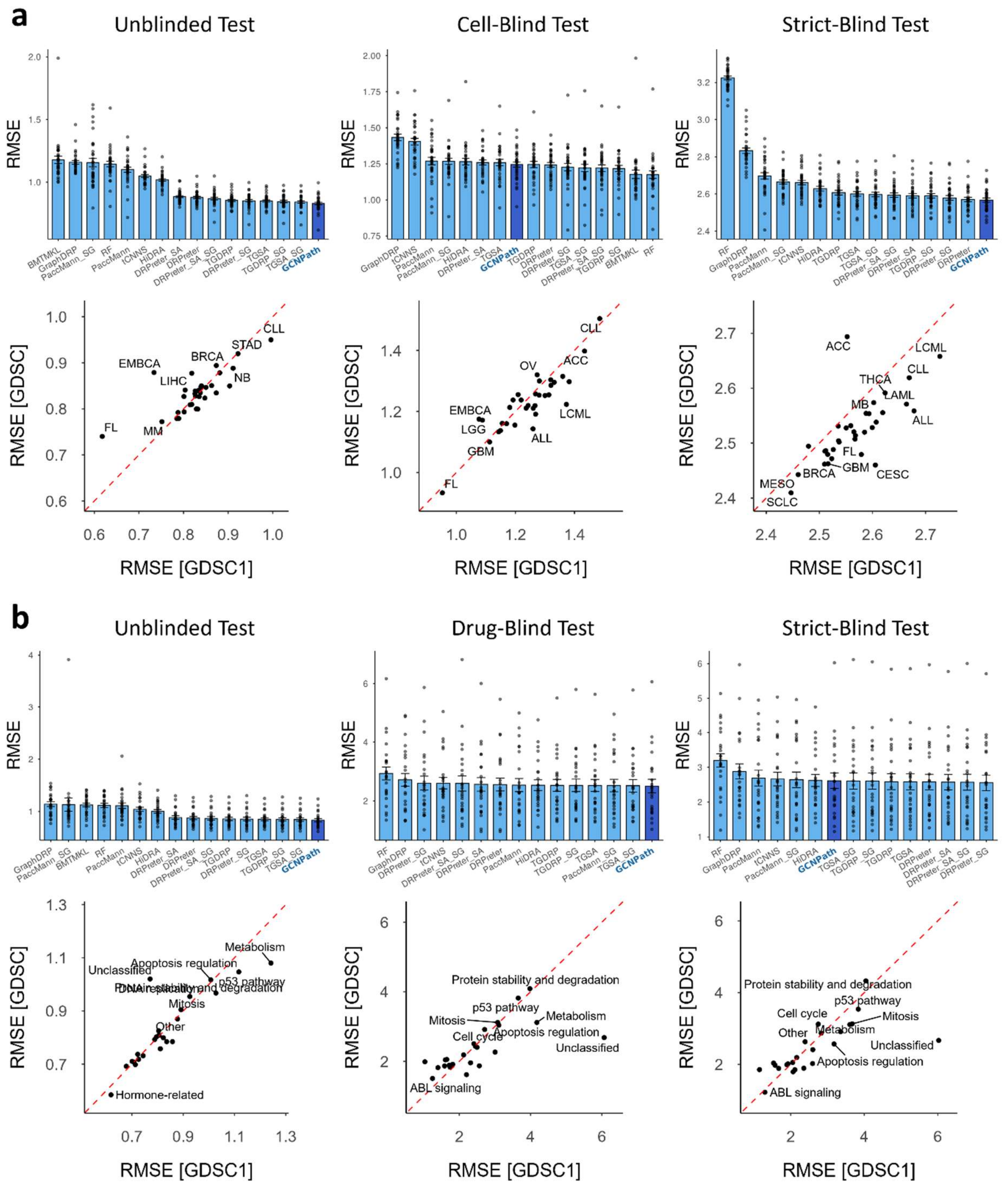

Supplementary Figure 18. RMSEs of various models tested using GDSC1 as the target label datasets, stratified by cell tissue types and drug target pathways. Each model was trained and evaluated using GDSC1 as the target label dataset, with 10-fold outer cross-validation applied in the (left) unblinded and (middle) cell-blind or drug-blind settings, and 25-fold outer cross-validation used in the (right) strict-blind test. RMSE values were computed for (**a**) 38 cell tissue types and (**b**) 24 drug target pathways, as annotated in the GDSC database. The results are presented as bar plots (top of

each panel), and scatter plots (bottom of each panel) comparing the RMSE values from GCNPath trained from GDSC1 and GDSC1+2, respectively. In all barplots, RMSEs are reported as mean ± standard deviation, represented by error bars. The source data are provided in Supplementary Data 19.

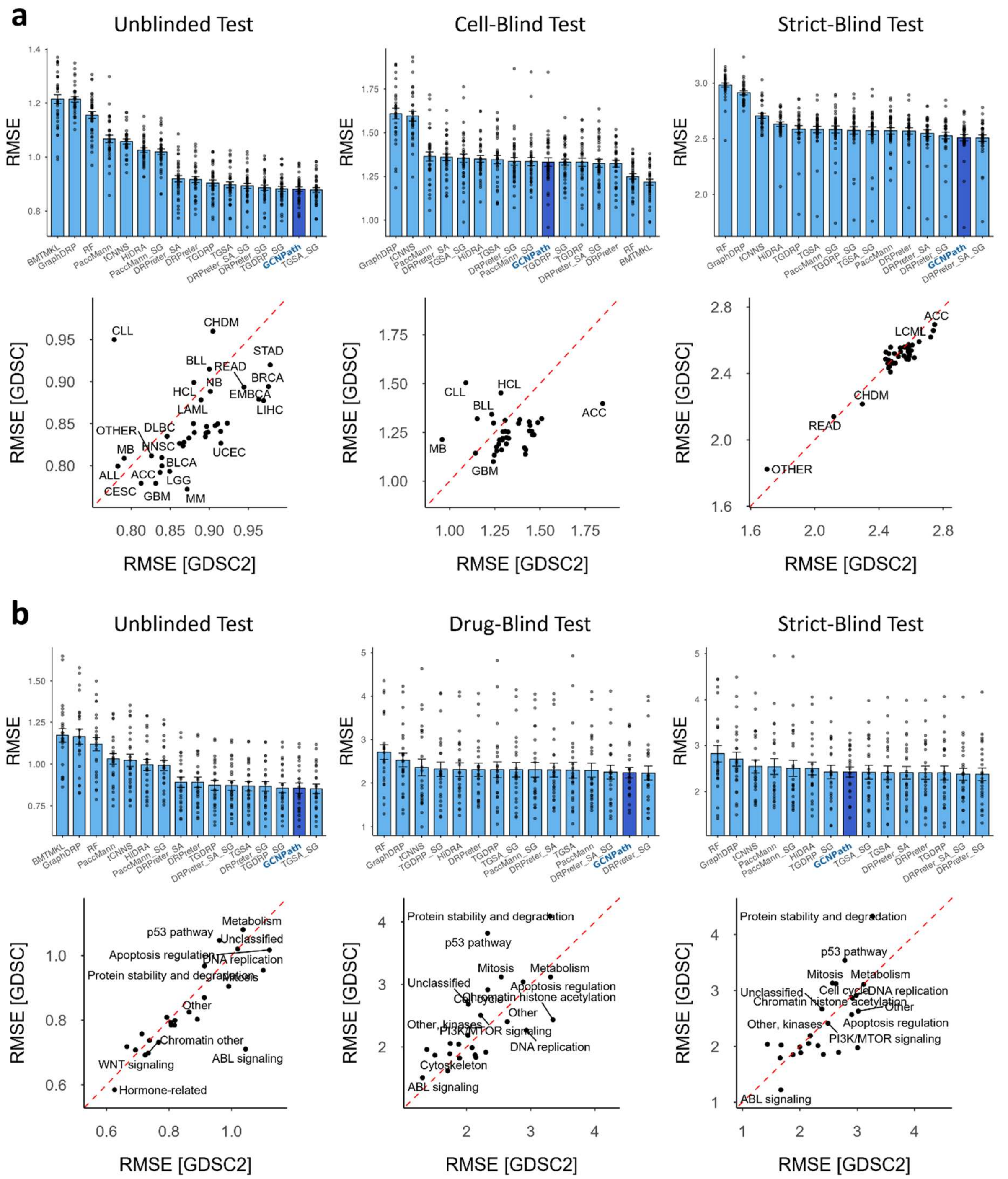

Supplementary Figure 19. RMSEs of various models tested using GDSC2 as the target label datasets, stratified by cell tissue types and drug target pathways. Each model was trained and evaluated using GDSC2 as the target label dataset, with 10-fold outer cross-validation applied in the (left) unblinded and (middle) cell-blind or drug-blind settings, and 25-fold outer cross-validation used in the (right) strict-blind test. RMSE values were computed for (**a**) 38 cell tissue types and (**b**) 24 drug target pathways, as annotated in the GDSC database. The results are presented as bar plots (top of

each panel), and scatter plots (bottom of each panel) comparing the RMSE values from GCNPath trained from GDSC2 and GDSC1+2, respectively. In all barplots, RMSEs are reported as mean ± standard deviation, represented by error bars. The source data are provided in Supplementary Data 20.

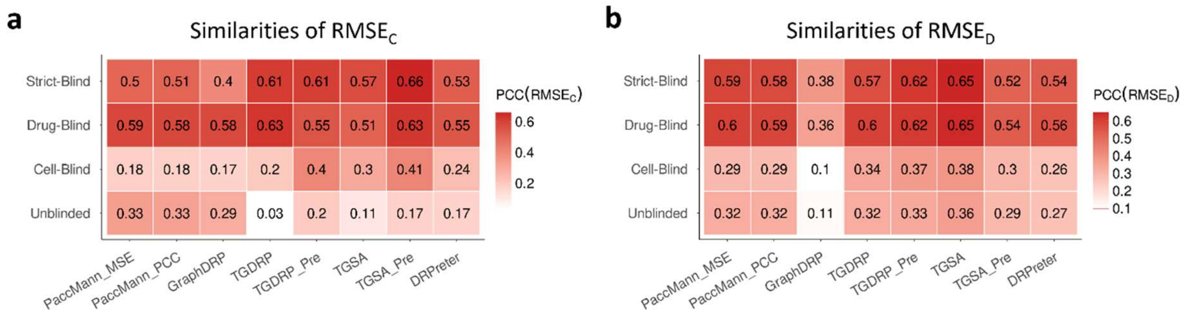

Supplementary Figure 20. PCCs of RMSE$_C$ and RMSE$_D$ between models trained in this study and those pretrained by their original developers using GDSC1+2 as a target label dataset. PaccMann, GraphDRP, TGDRP, TGSA, and DRPreter were trained from scratch and tested via 10-fold outer cross-validation for unblinded, cell-blind, and drug-blind tests, and 25-fold outer cross-validation for strict-blind tests. RMSE$_C$ and RMSE$_D$ were calculated for each cell line and drug, respectively. Predictions from pretrained models by their creators, also based on GDSC1+2, were evaluated using the same procedure. PCCs of (**a**) RMSE$_C$ and (**b**) RMSE$_D$ were then calculated to assess the similarity between trained and pretrained models in predicting cell lines and drugs, respectively. For DRPreter, ten models were pretrained with random seeds (2, 16, 33, 61, 79, 100, 220, 653, 1004, 4001), and results from the model with the lowest RMSE (seed 4001) are shown. The source data are provided in Supplementary Data 21. PaccMann_MSE, PaccMann_PCC: PaccMann pretrained to minimize MSE or maximize PCC. TGDRP_Pre, TGSA_Pre: TGDRP and TGSA pretrained with self-supervised learning of the GCN drug module using ZINC15 and ChEMBL datasets, respectively.

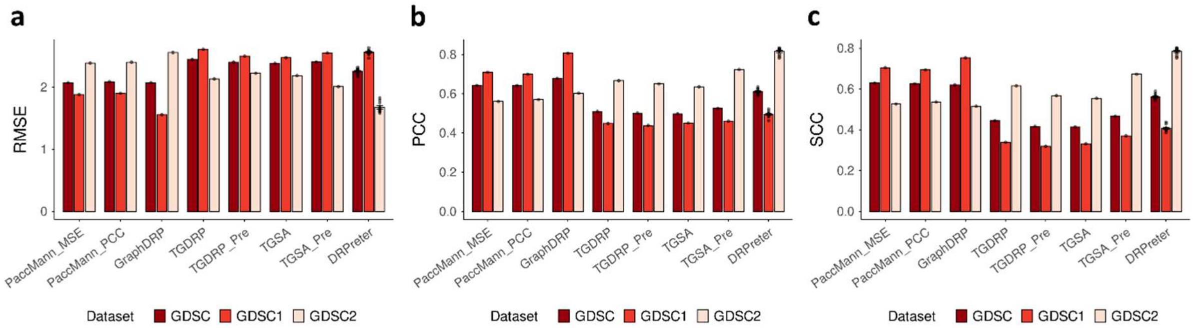

Supplementary Figure 21. Performance of the models pretrained by their original developers using GDSC datasets as target label datasets. PaccMann, GraphDRP, TGDRP, TGSA, and DRPreter, pretrained by their respective creators, were evaluated on GDSC datasets. The following metrics were calculated: (a) RMSEs, (b) PCCs, and (c) SCCs. For DRPreter, ten models were pretrained using random seeds (2, 16, 33, 61, 79, 100, 220, 653, 1004, 4001). All ten models were used for prediction, generating ten RMSEs, PCCs, and SCCs. These metrics are reported as mean ± standard deviation, represented by error bars. The source data are provided in Supplementary Data 22. PaccMann_MSE, PaccMann_PCC: PaccMann pretrained to minimize MSE or maximize PCC, respectively. TGDRP_Pre, TGSA_Pre: TGDRP and TGSA pretrained via self-supervised learning of the GCN drug module using ZINC15 and ChEMBL datasets.

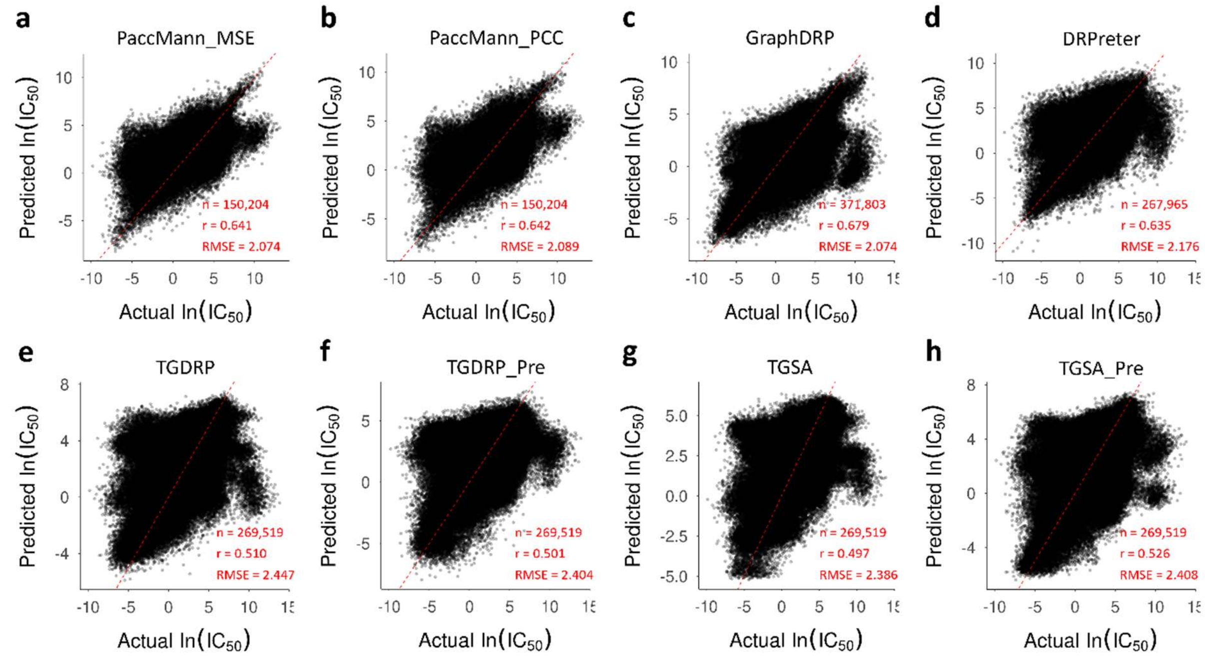

Supplementary Figure 22. Scatter plots of predictions from models pretrained by their respective developers using GDSC1+2 as a target label dataset. Each model, pretrained by its original developers, was used to predict $\ln(IC_{50})$ values with GDSC1+2 as a target dataset. The scatter plots display the number of predicted values (n), prediction PCC (r), and RMSE, annotated in red text. For DRPreter, predictions were averaged based on ten models pretrained with random seeds (2, 16, 33, 61, 79, 100, 220, 653, 1004, 4001). The scatter plot shows results from the model with the lowest RMSE (seed 4001). The source data are provided in Supplementary Data 23. PaccMann_MSE, PaccMann_PCC: PaccMann pretrained to minimize MSE or maximize PCC, respectively. TGDRP_Pre, TGSA_Pre: TGDRP and TGSA pretrained via self-supervised learning of the GCN drug module using ZINC15 and ChEMBL datasets.

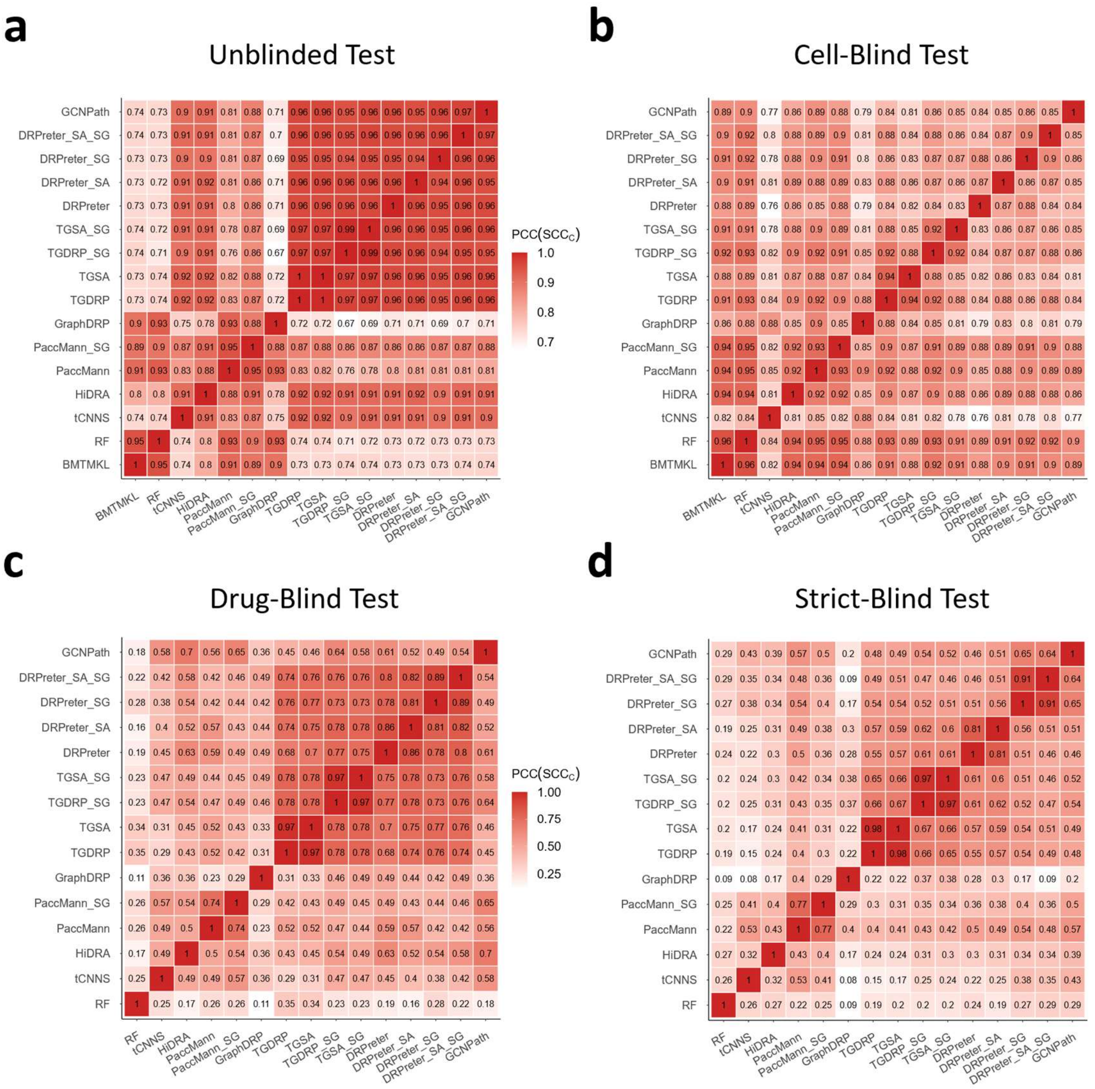

Supplementary Figure 23. PCCs of prediction SCC values per cell line (SCC_C) for models trained with GDSC1+2 as a target label dataset. Each model was trained and tested using GDSC1+2 as a target label dataset, with 10-fold outer cross-validation for unblinded, cell-blind, and drug-blind tests, and 25-fold outer cross-validation for strict-blind tests. SCC values per cell line (SCC_C) were computed based on model predictions in each test to assess cell line prediction performance. PCCs of SCC_C values were calculated for each model pair and test type to evaluate the similarity in cell line predictability. Results from the (**a**) unblinded, (**b**) cell-blind, (**c**) drug-blind, and (**d**) strict-blind tests were compared. The source data are provided in Supplementary Data 24.

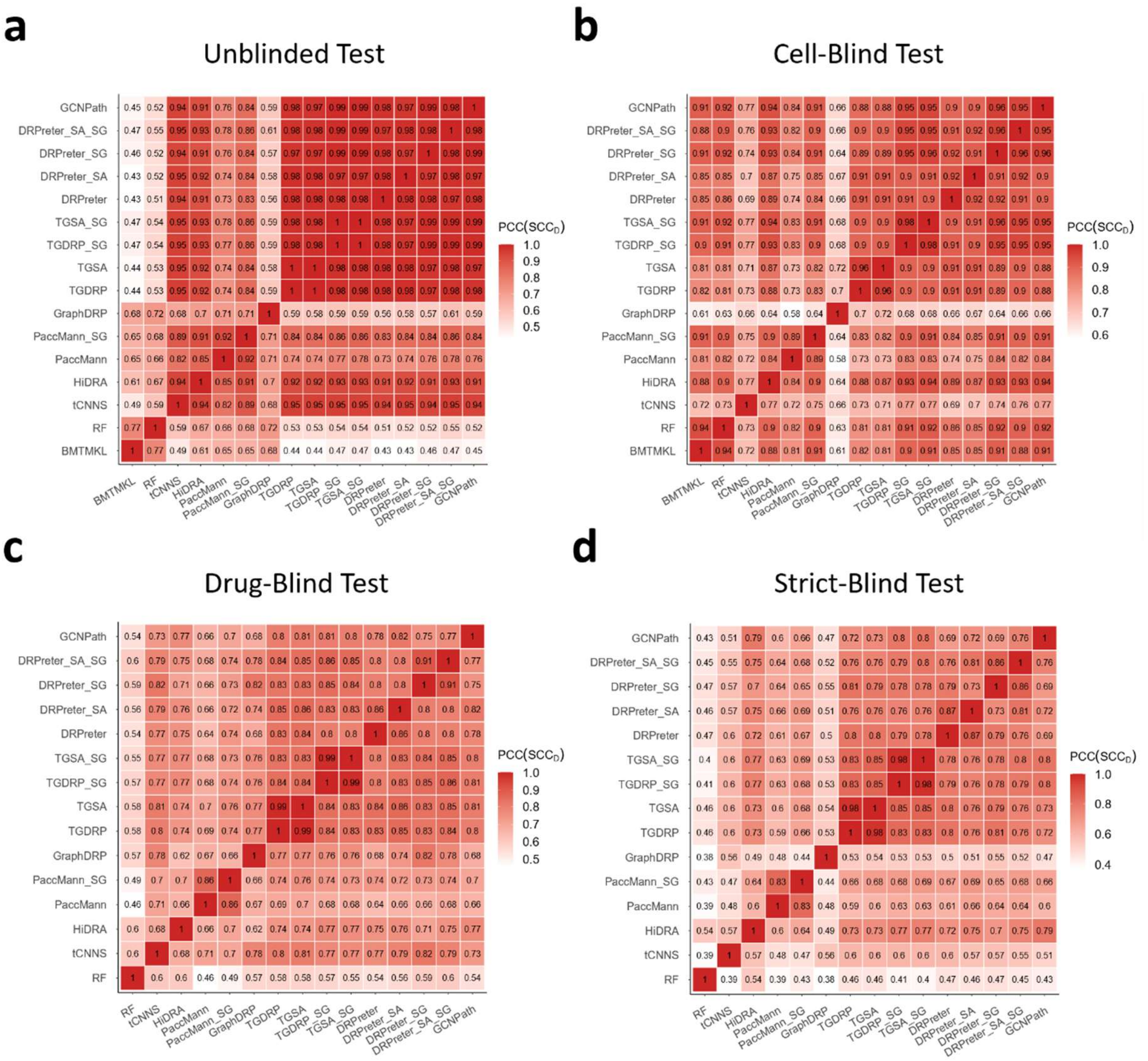

Supplementary Figure 24. PCCs of prediction SCC values per drug (SCC$_D$) for models trained with GDSC1+2 as a target label dataset. Each model was trained and tested using GDSC1+2 as a target label dataset, employing 10-fold outer cross-validation for unblinded, cell-blind, and drug-blind tests, and 25-fold outer cross-validation for strict-blind tests. SCC values per drug (SCC$_D$) were computed from model predictions in each test to evaluate drug prediction performance. PCCs of SCC$_D$ values were calculated for each model pair and test type to assess similarity in drug predictive capacity. Results from the (**a**) unblinded, (**b**) cell-blind, (**c**) drug-blind, and (**d**) strict-blind tests were compared. The source data are provided in Supplementary Data 25.

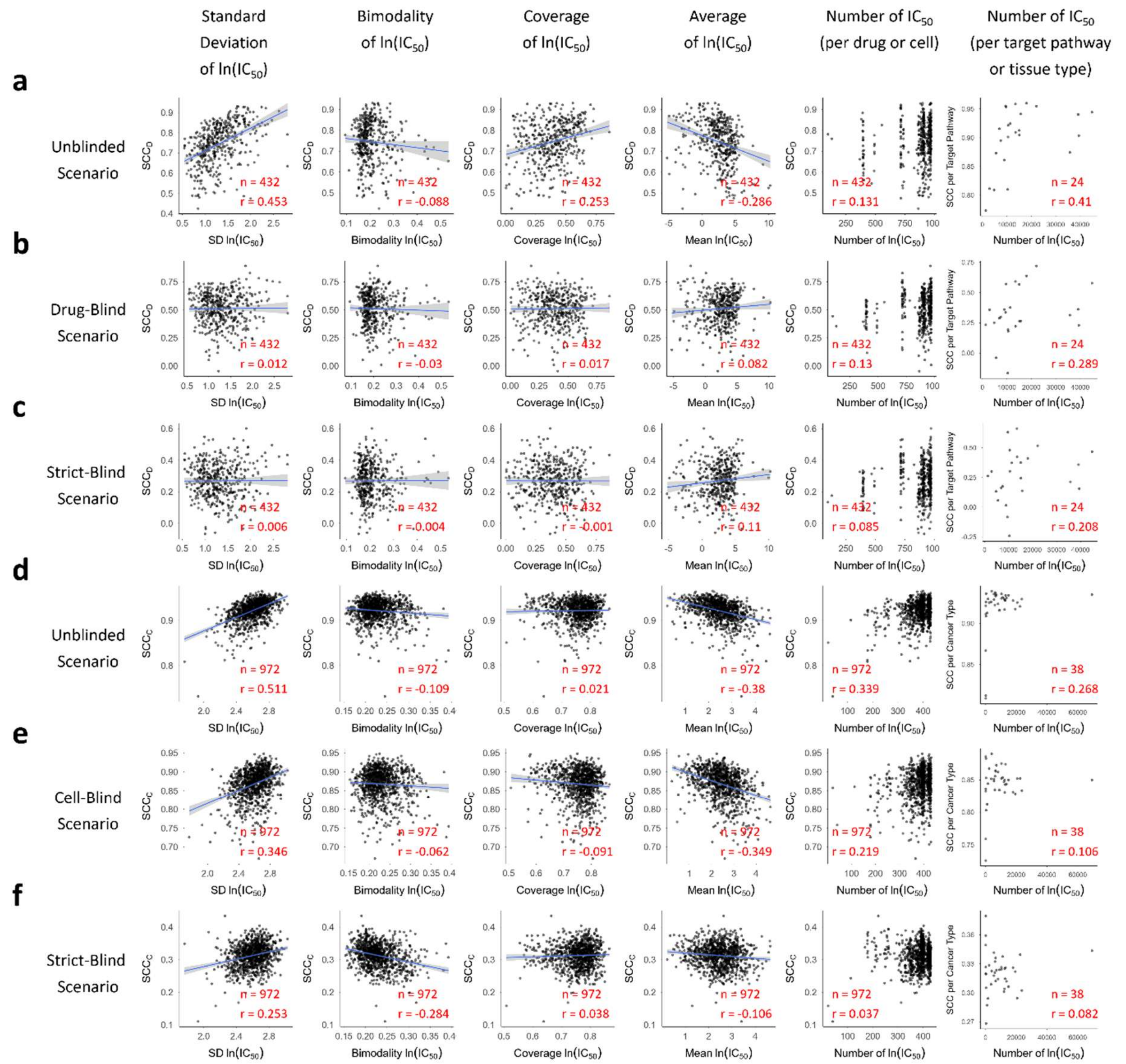

Supplementary Figure 25. Performance analysis using GDSC1+2 dataset. GCNPath was trained and tested on GDSC1+2 as a target label dataset using 10-fold outer cross-validation for unblinded, cell-blind or drug-blind tests and 25-fold outer cross-validation for strict-blind tests. Prediction performance was assessed using (**a-c**) SCC_D and (**d-f**) SCC_C, calculated across 432 drugs and 972 cell lines. PCCs were computed between these SCC_D and SCC_C values with the following six metrics: (1) Standard deviation of ln(IC_{50}) indicating the dispersion of drug responses for each drug or cell line, (2) Bimodality of ln(IC_{50}) reflecting selective anticancer activity, (3) Density coverage of ln(IC_{50}) representing the similarity between the ln(IC_{50}) distribution for each drug or cell line and that of the entire GDSC1+2 dataset, (4) Average of ln(IC_{50}) indicating the overall level of anticancer efficacy, (5, 6) Number of available ln(IC_{50}) values per drug or cell line, or per target pathways or tissue types. Each scatter plot shows the number of data points (n) and the corresponding PCC (r), displayed in red, for

(**a-c**) drugs (left and middle) and their target pathways (right), and cell lines (left and middle) and their cancer tissue types (right). The source data are provided in Supplementary Data 26.

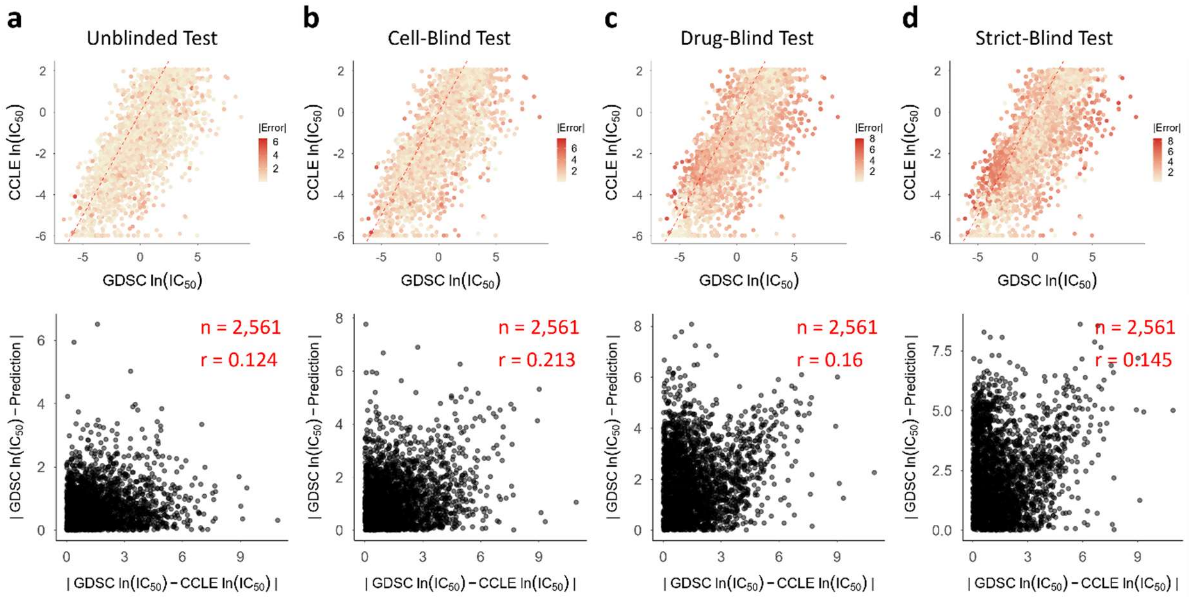

Supplementary Figure 26. **Correlation analysis of prediction errors and intrinsic variation in ln(IC_{50}) values.** GCNPath was trained and tested on the GDSC1+2 as a target label dataset using 10-fold outer cross-validation for (**a**) unblinded, (**b**) cell-blind and (**c**) drug-blind tests, and 25-fold outer cross-validation for (**d**) strict-blind tests. Prediction performances were calculated as the absolute differences between predicted and actual ln(IC_{50}) values from GDSC1+2, based on 2,561 common drug response entries between GDSC1+2 and CCLE. In the CCLE dataset, drug response values capped at 8 μM were excluded beforehand. Prediction errors were then visualized with red intensity indicating higher error (top panel) and the y-axis (bottom panel). Intrinsic variation in ln(IC_{50}) values, defined as the absolute differences between actual values from GDSC1+2 and CCLE, is shown on the x-axis (bottom panel). The number of ln(IC_{50}) values (n) and PCC (r) were displayed in red text (bottom panel). The source data are provided in Supplementary Data 27.

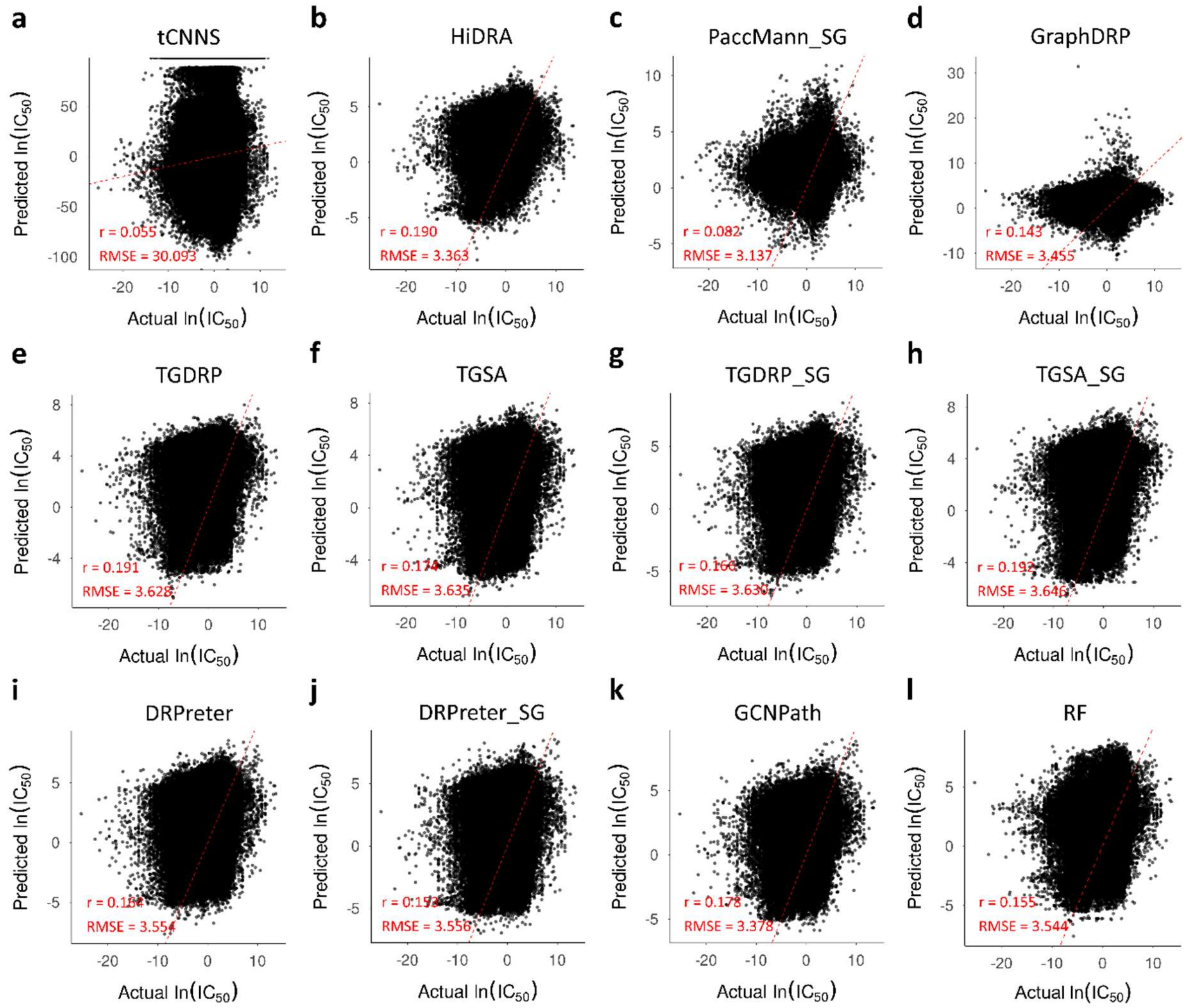

Supplementary Figure 27. Scatter plots of model predictions with the ChEMBL as a target label dataset. Each model was trained ten times with the entire GDSC1+2 and tested with ChEMBL as target label datasets, and the results of the model with the lowest RMSE are plotted. The prediction PCC (r) and RMSE are indicated in each scatter plot using red text. Unexpectedly, tCNNS output values of infinity for 29,233 of the 237,580 ln(IC_{50}) values. These outliers were excluded from the calculations. DRPreter models with a similarity augmentation module (DRPreter_SA, DRPreter_SA_SG) were excluded because they cannot predict the responses of drugs not included during the training phase. The source data are provided in Supplementary Data 29. SG: model trained with cell line data from SANGER Cell Model Passports as cell-line input data.

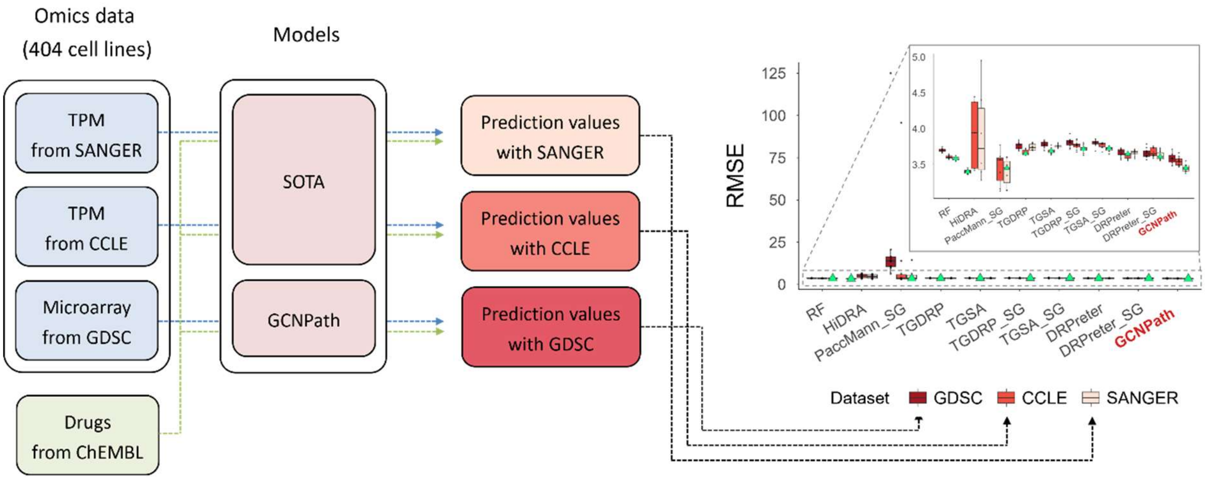

Supplementary Figure 28. The workflow in prediction of ChEMBL as a target label dataset using a set of gene expression datasets. Each model was first trained on the entire GDSC1+2 dataset without any data splitting. Gene expression data from 404 cell lines, obtained from SANGER Cell Model Passports, CCLE, and GDSC, were then input into the trained models separately for each gene expression dataset, generating three types of prediction values. These predictions were compared to the actual ln(IC_{50}) values from ChEMBL to compute the prediction metrics—RMSE, PCC, and SCC—for each model and gene expression dataset. For models using multi-omics input (TGDRP, TGSA, TGDRP_SG, and TGSA_SG), only the gene expression data were varied; mutation and CNV data were kept constant.

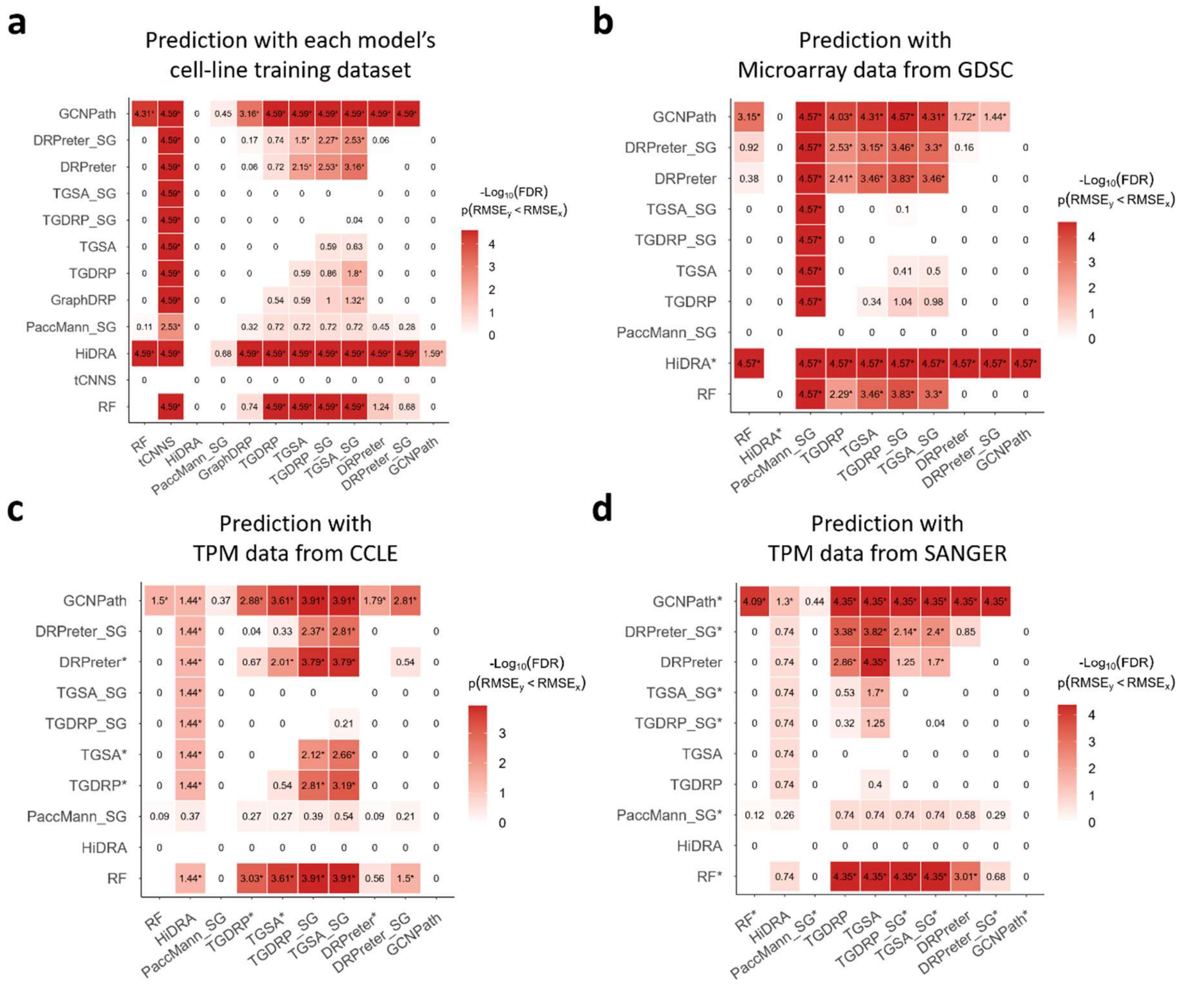

Supplementary Figure 29. U tests of model RMSEs with the ChEMBL as a target label dataset. All models utilizing gene expression data were trained ten times with the entire GDSC1+2 and tested with the ChEMBL as target label datasets. For prediction of ChEMBL, the following cell line datasets were used as inputs: (**a**) the training dataset specific to each model, (**b**) microarray data from GDSC, (**c**) TPM data from CCLE, and (**d**) TPM data from SANGER Cell Model Passports. Consequently, ten RMSEs were computed for each model and cell line dataset combination. The performance of each model with its own training cell line dataset is indicated with an asterisk in (**b-d**). The $-\text{Log}_{10}$-scaled FDRs were calculated to assess whether the models on the y-axis exhibit significantly lower RMSEs than those on the x-axis. FDR values were obtained from the one-tailed Mann–Whitney test. The source data are provided in Supplementary Data 30. *: FDR ≤ 0.05. SG: model trained with cell line data from SANGER Cell Model Passports as cell-line input data.

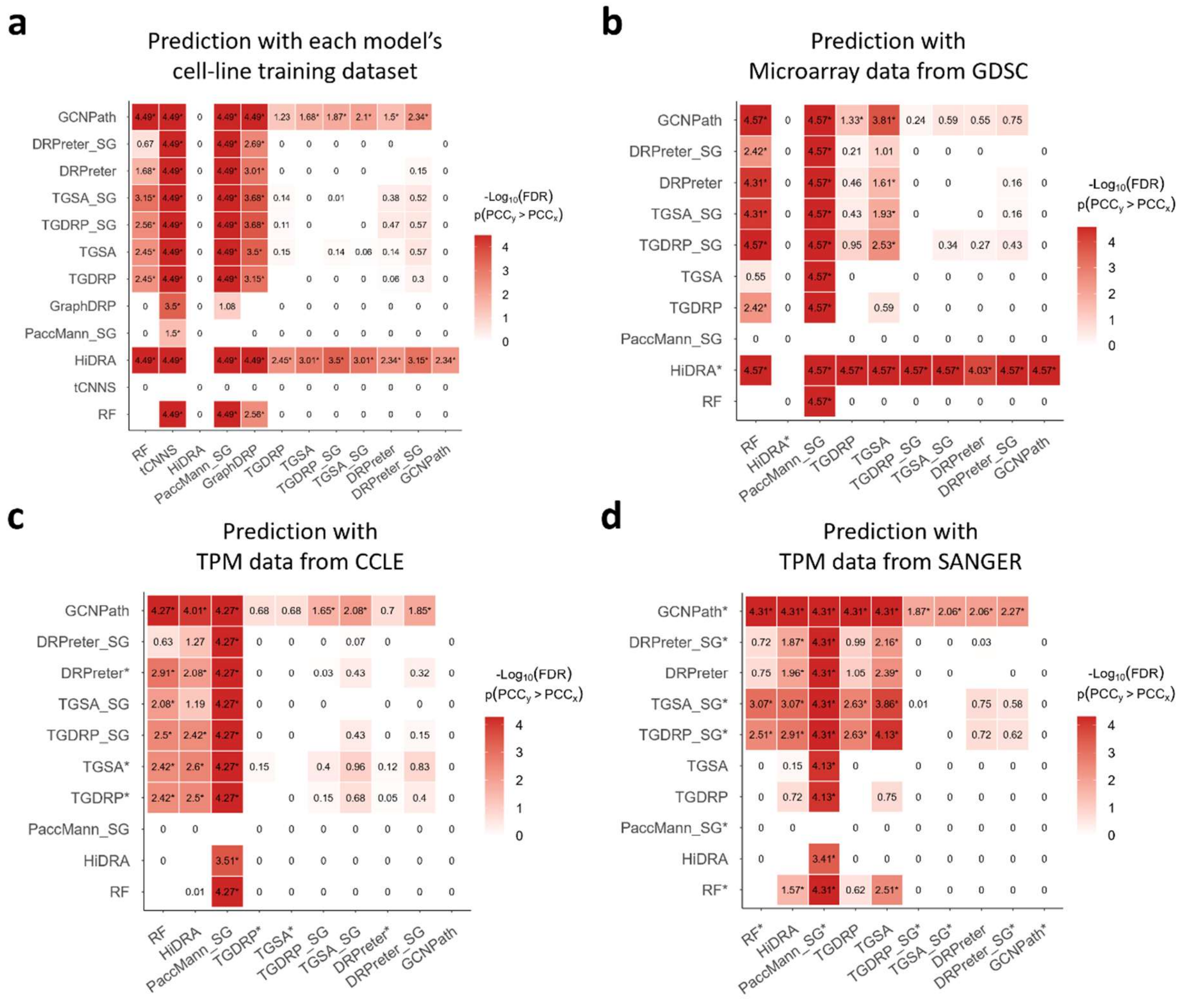

Supplementary Figure 30. U tests of model PCCs with the ChEMBL as a target label dataset. All models utilizing gene expression data were trained ten times with the entire GDSC1+2 and tested with the ChEMBL as target label datasets. For prediction of ChEMBL, the following cell line datasets were used as inputs: (**a**) the training dataset specific to each model, (**b**) microarray data from GDSC, (**c**) TPM data from CCLE, and (**d**) TPM data from SANGER Cell Model Passports. Consequently, ten PCCs were computed for each model and cell line dataset combination. The performance of each model with its own training cell line dataset is marked with an asterisk in (**b-d**). The $-\text{Log}_{10}$-scaled FDRs were calculated to assess whether the models on the y-axis exhibit significantly greater PCCs than those on the x-axis. FDR values were obtained from the one-tailed Mann–Whitney test. The source data are provided in Supplementary Data 31. *: FDR ≤ 0.05. SG: model trained with cell line data from SANGER Cell Model Passports as cell-line input data.

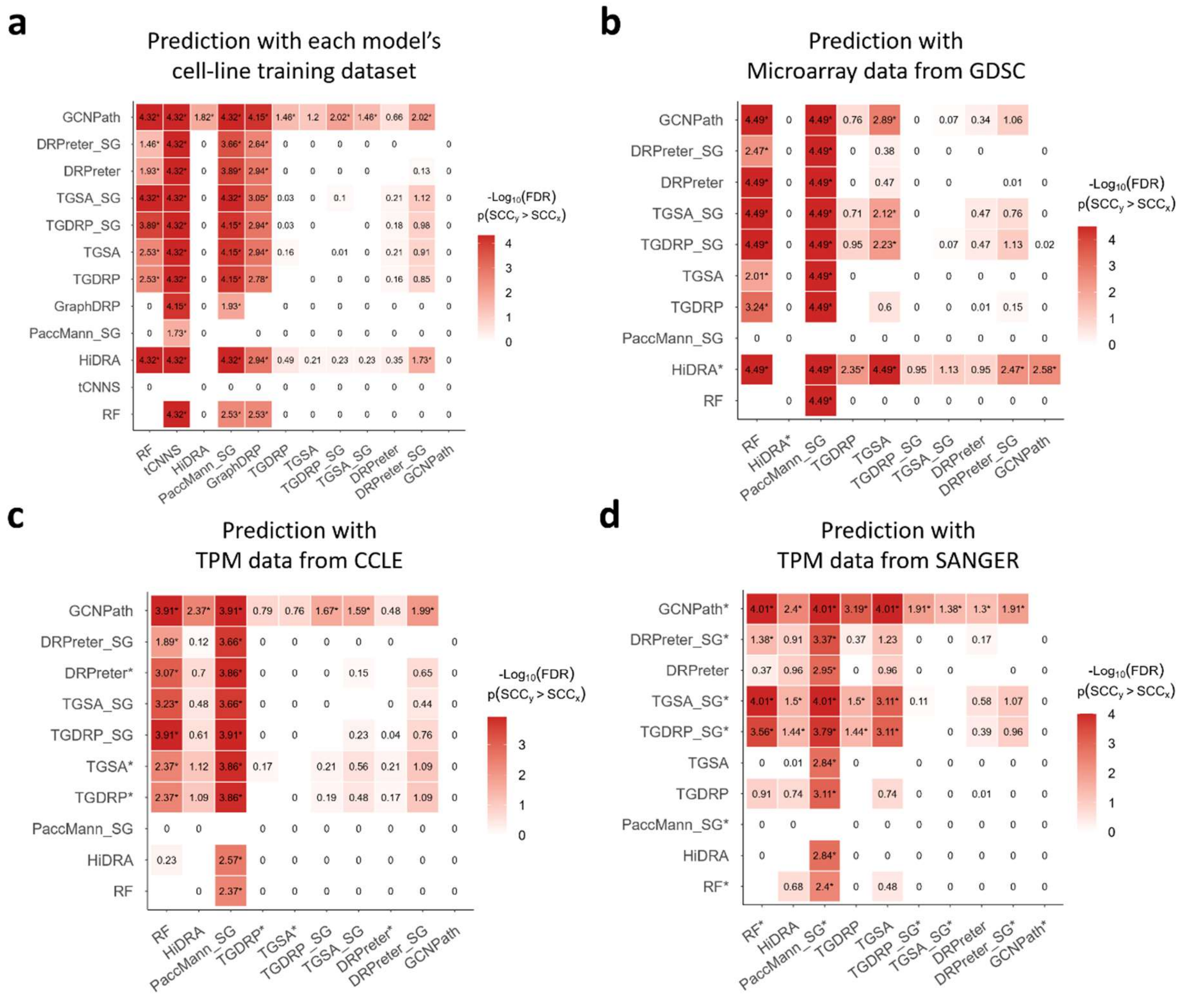

Supplementary Figure 31. U tests of model SCCs with the ChEMBL as a target label dataset. All models utilizing gene expression data were trained ten times with the entire GDSC1+2 and tested with the ChEMBL as target label datasets. For prediction of ChEMBL, the following cell line datasets were used as inputs: (**a**) the training dataset specific to each model, (**b**) microarray data from GDSC, (**c**) TPM data from CCLE, and (**d**) TPM data from SANGER Cell Model Passports. Consequently, ten SCCs were computed for each model and cell line dataset combination. The performance of each model with its own training cell line dataset is marked with an asterisk in (**b-d**). The $-\text{Log}_{10}$-scaled FDRs were calculated to assess whether the models on the y-axis exhibit significantly greater SCCs than those on the x-axis. FDR values were obtained from the one-tailed Mann–Whitney test. The source data are provided in Supplementary Data 32. *: FDR ≤ 0.05. SG: model trained with cell line data from SANGER Cell Model Passports as cell-line input data.

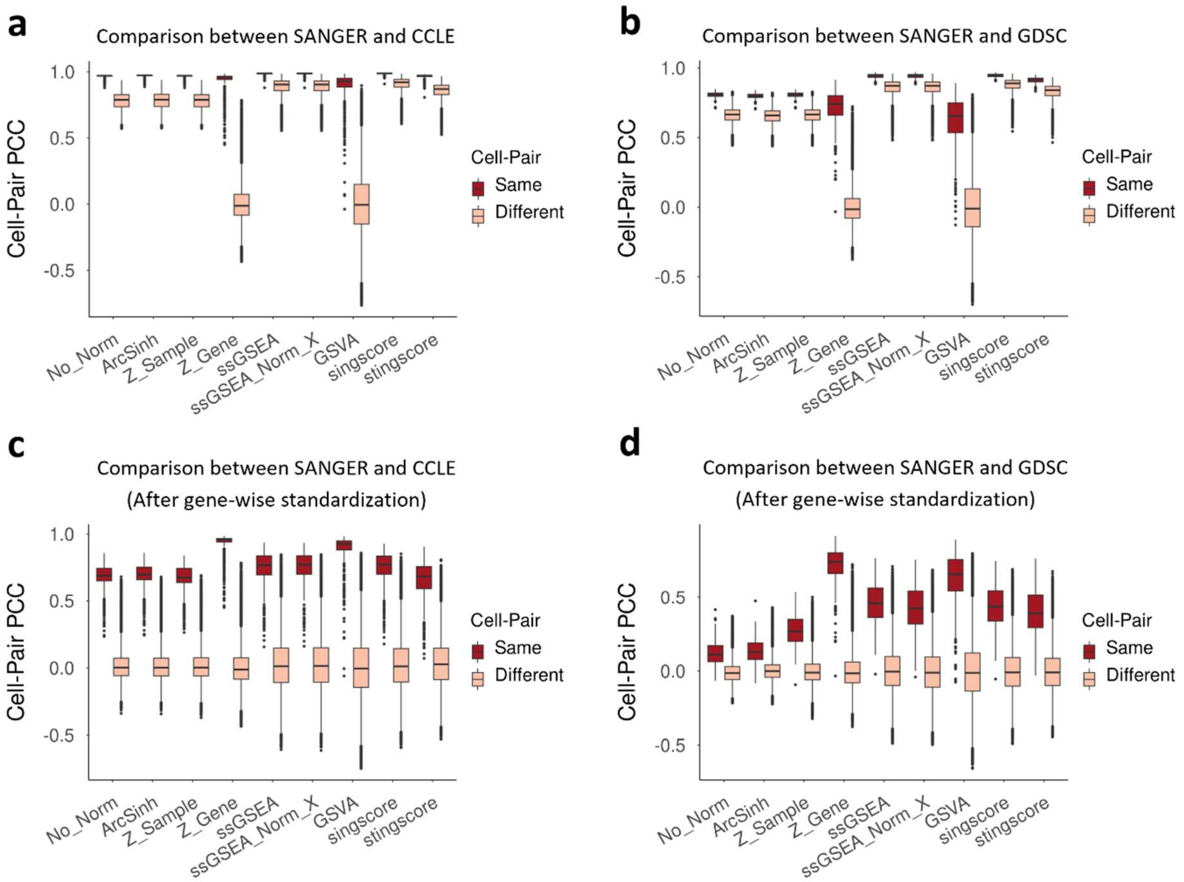

Supplementary Figure 32. Boxplots assessing batch correction via gene expression feature preprocessing. The 404 cell lines common in TPM data from SANGER Cell Model Passports, TPM data from CCLE, and the microarray data from GDSC were utilized for prediction of ln(IC_{50}) values from ChEMBL, respectively. Using these overlapping cell lines, the distributions of the three datasets were visualized with boxplots, categorized by identical and different cell pairs. The gene expression data from SANGER were compared with those from (**a**) CCLE and (**b**) GDSC. The same analysis was performed after gene- or pathway-wise standardization (**c, d**), transforming CCLE and GDSC datasets with means and standard variations in gene expression from SANGER. Note that except the ArcSinh normalization, TPM data from SANGER and CCLE were basically normalized in log2-scale with pseudo-count 1. Boxplots display the IQR, with whiskers extending 1.5×IQR. The source data are provided in Supplementary Data 34. No_Norm: no additional normalization methods applied beyond $\log_2$ transformation or RMA; Z_Sample: z-score standardization of gene expression within a cell line; Z_Gene: z-score standardization of gene expression for each gene. The standardization were performed with means and standardizations of gene expression from each dataset; ssGSEA: pathway activation scores calculated with single-sample gene set enrichment analysis; ssGSEA_Norm_X:

ssGSEA without across-sample normalization (parameter ssgsea.norm=FALSE); GSVA: pathway activation scores calculated with gene set variation analysis; singscore: pathway activation scores calculated with simple single-sample gene signature scorings; stingscore: singscore calculated using 100 genes showing stable expression across various cancers; which are also included in the 292 BIOCARTA pathways and the TPM data from SANGER Cell Model Passports. Of these genes, 100, and 93 overlapped with the datasets from CCLE, and GDSC, respectively.

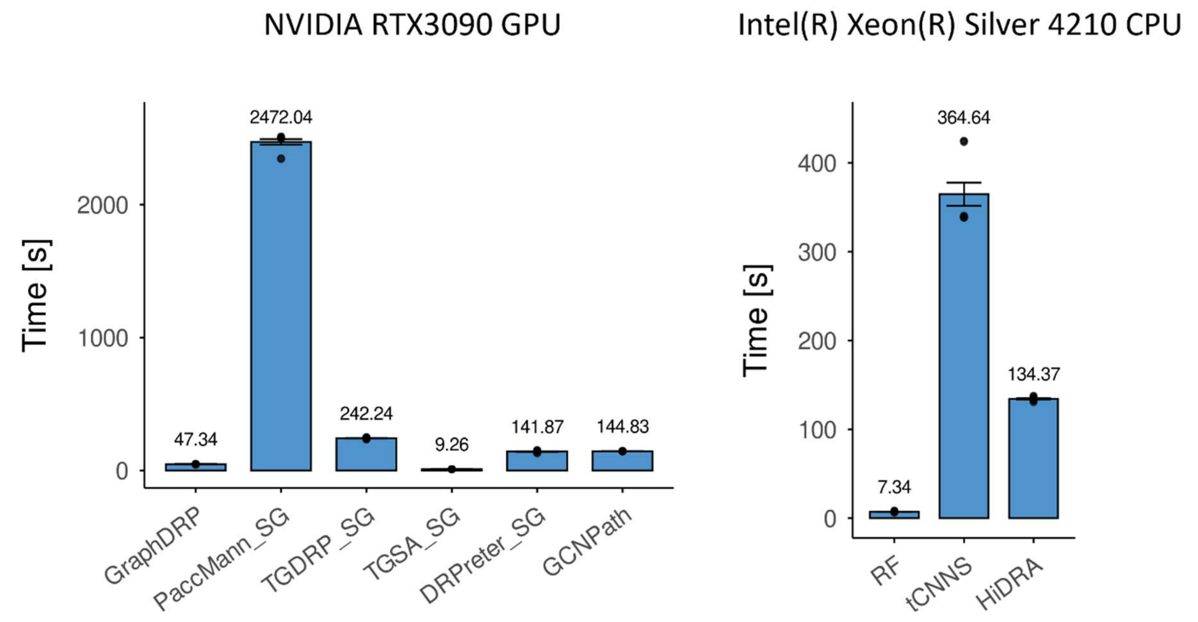

Supplementary Figure 33. Comparison of inference times for each model using the ChEMBL dataset. Inference time was measured in seconds for models that were compatible with (left) an NVIDIA RTX 3090 GPU and (right) those that were not, the latter being run on an Intel® Xeon® Silver 4210 CPU. All models utilized a unified setting of four subprocesses on the Intel® Xeon® Silver 4210 CPU for data loading — implemented via num_workers=4 in the PyTorch DataLoader, workers=4 in Keras predict_generator (HiDRA), or tf.ConfigProto(intra_op_parallelism_threads=4, inter_op_parallelism_threads=1) in TensorFlow (tCNNS). Inference time was measured using either torch.cuda.synchronize() for GPU-compatible models or time.perf_counter() from Python's time module for CPU-only models. Each prediction time in seconds is reported as mean ± standard deviation, represented by error bars (n=10 in each bar). The source data are provided in Supplementary Data 35.

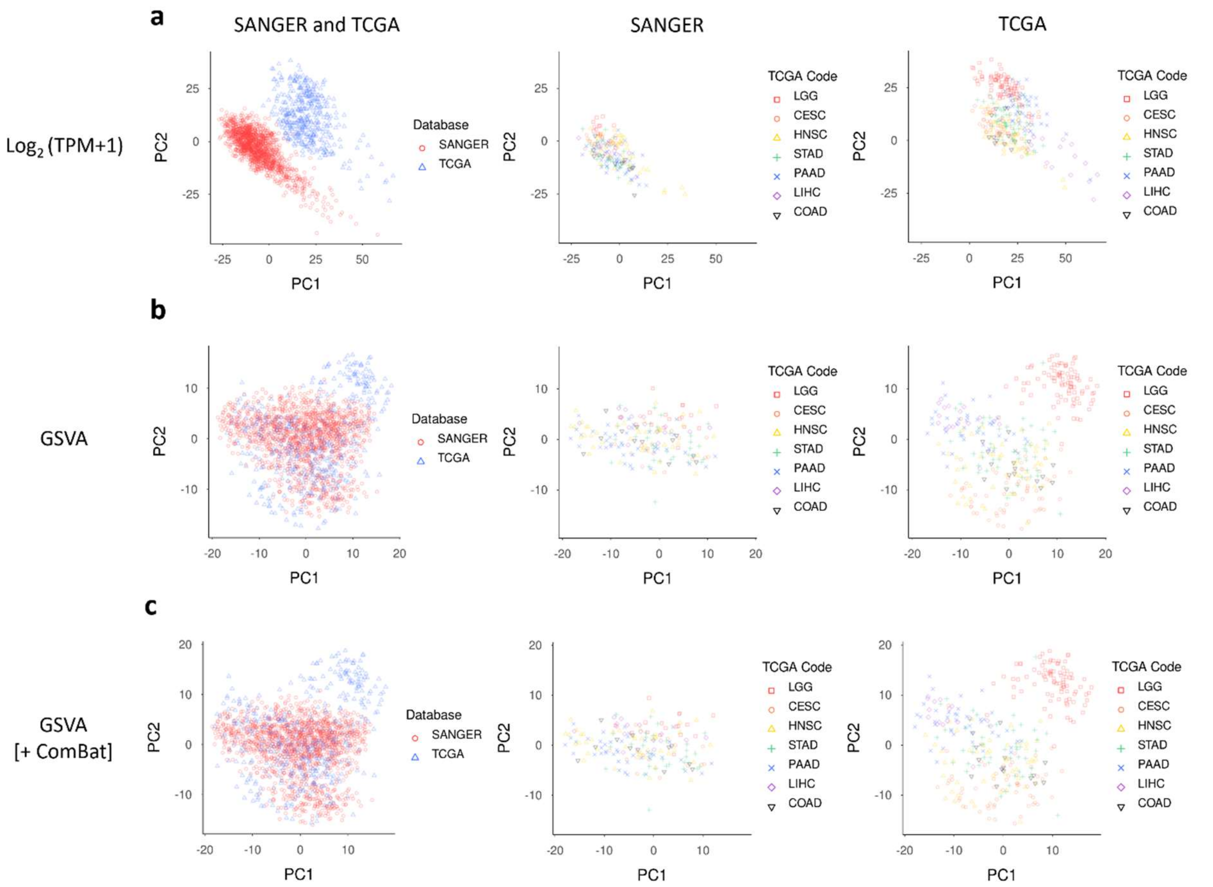

Supplementary Figure 34. PCA plots comparing TPM and GSVA scores from SANGER Cell Model Passports and TCGA datasets. Of the 1,431 cell lines in SANGER Cell Model Passports, only 546 cell lines corresponding to the same cancer tissue types as the 414 samples from 399 TCGA patients were retained. From these two dataset, three data representations were visualized using PC2 plots: (**a**) log2-scaled TPM values with pseudo-count 1, (**b**) GSVA scores computed from the raw data, and (**c**) GSVA scores after applying ComBat batch correction using the SANGER Cell Model Passports as the reference and cancer tissue type as the matching variable. In (**a**), only 1,503 genes that were shared between the two datasets and annotated in BIOCARTA pathways were used. The top 7 most common tissue types among the 414 TCGA samples are displayed in SANGER Cell Model Passports (middle) and TCGA (right). The source data are provided in Supplementary Data 36.

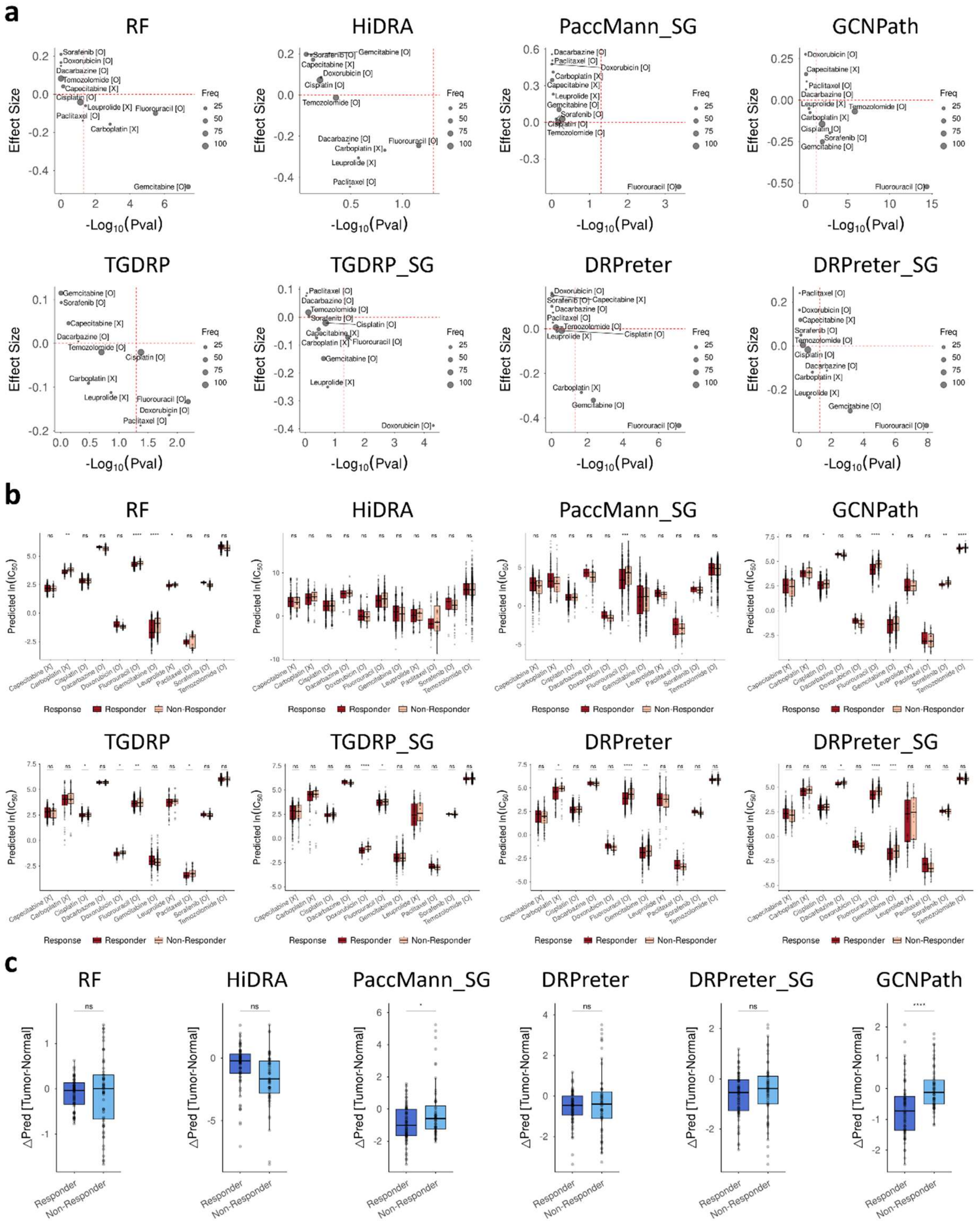

Supplementary Figure 35. Benchmark test with clinical data from the TCGA dataset, after batch correction with reference ComBat. Most models were trained ten times using the entire GDSC1+2 dataset before application. The gene expression data utilized for each DRP model were processed with ComBat batch correction using SANGER Cell Model Passports as the reference and cancer tissue type as the matching variable. tCNNS and GraphDRP were excluded because their required input features

are not available in TCGA dataset. Additionally, TGSA, TGSA_SG, DRPreter_SA, and DRPreter_SA_SG—which are unable to predict drugs not included in the GDSC dataset—were also not included. Since the response data for each drug were limited, the predictions were not aggregated across models. In (**a, b**), 'O' and 'X' in brackets show whether the drugs were present in the GDSC1+2 dataset (e.g., cisplatin [O] was screened in GDSC). In (**a-c**), p-values were obtained from a one-tailed Mann–Whitney test, and effect sizes in (**a, b**) were calculated as the difference in average predictions between responder and non-responder classes. Drugs with significant predicted effectiveness for responders appear in the bottom right ($p < 0.05$, effect size < 0). Other regions indicate low predicted significance (left) or inconsistency between predicted and actual responses (up). In (**c**), the prediction distributions across 13 tumor and matched normal sample pairs were shown. For TGDRP and TGDRP_SG, mutation and copy-number variation (CNV) profiles from matched-normal samples were unavailable. In (**a, c**), boxes span the IQR, with whiskers extending 1.5×IQR. The source data are provided in Supplementary Data 38. *: $p \leq 0.05$, **: $p \leq 0.01$, ***: $p \leq 0.001$, ****: $p \leq 0.0001$.

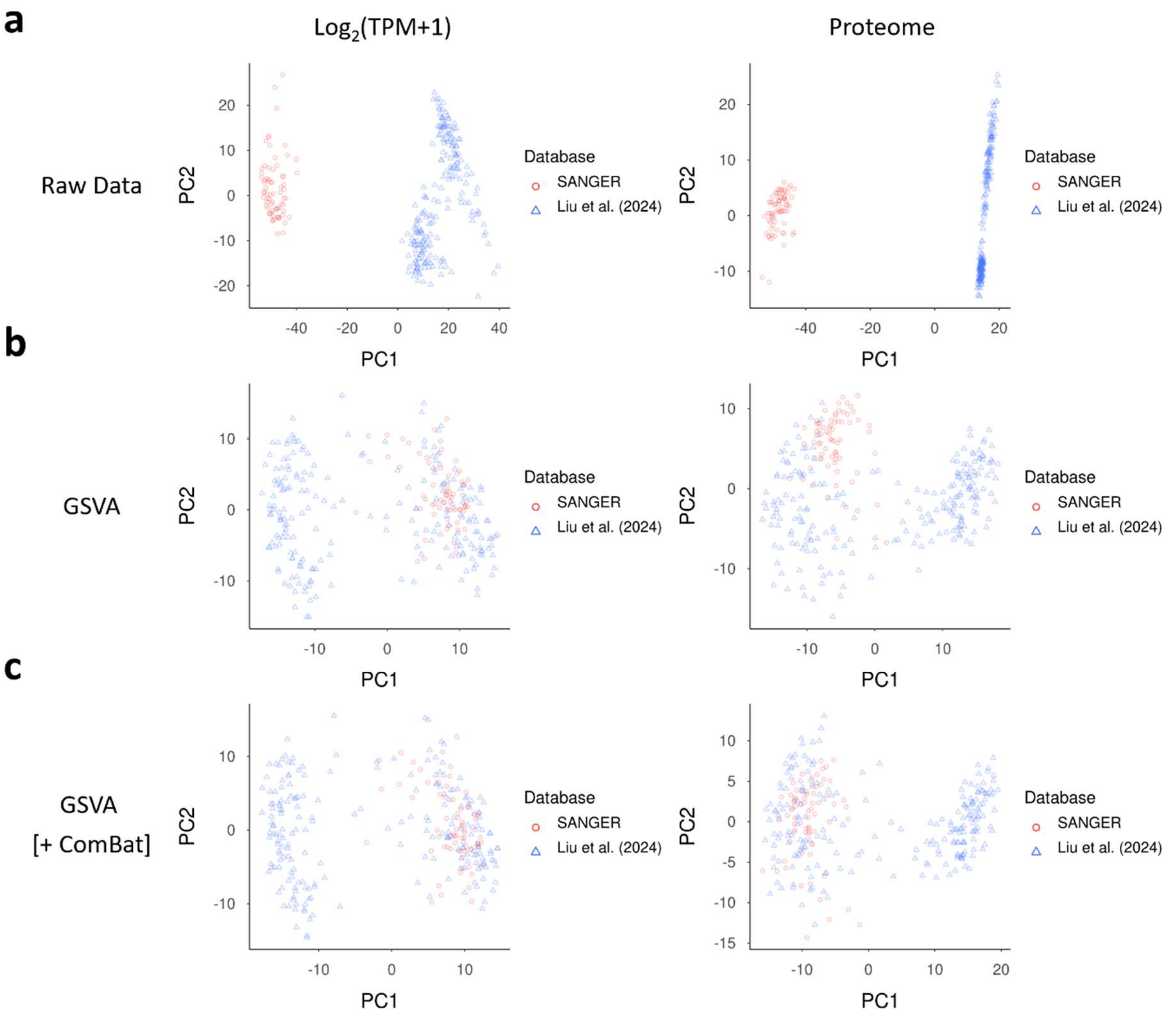

Supplementary Figure 36. PCA plots comparing raw data and GSVA scores from SANGER Cell Model Passports and the clinical dataset from Liu et al. (2024)[1]. Of the 1,431 cell lines in the SANGER Cell Model Passports, only 72 small cell lung cancer (SCLC) cell lines were retained. These were compared against log2-scaled TPM data with a pseudo-count of 1 (left) and proteomic data (right) from 107 patient cohorts reported by Liu et al. (2024)[1]. Three data representations were visualized using PC2 plots: (**a**) raw data, (**b**) GSVA scores computed from the raw data, and (**c**) GSVA scores after applying ComBat batch correction using SANGER Cell Model Passports as the reference and cancer/normal status as the matching variable. In (**a**), only 1,443 genes and 1,014 proteins that were shared between the two datasets and annotated in BIOCARTA pathways were used. The source data are provided in Supplementary Data 41.

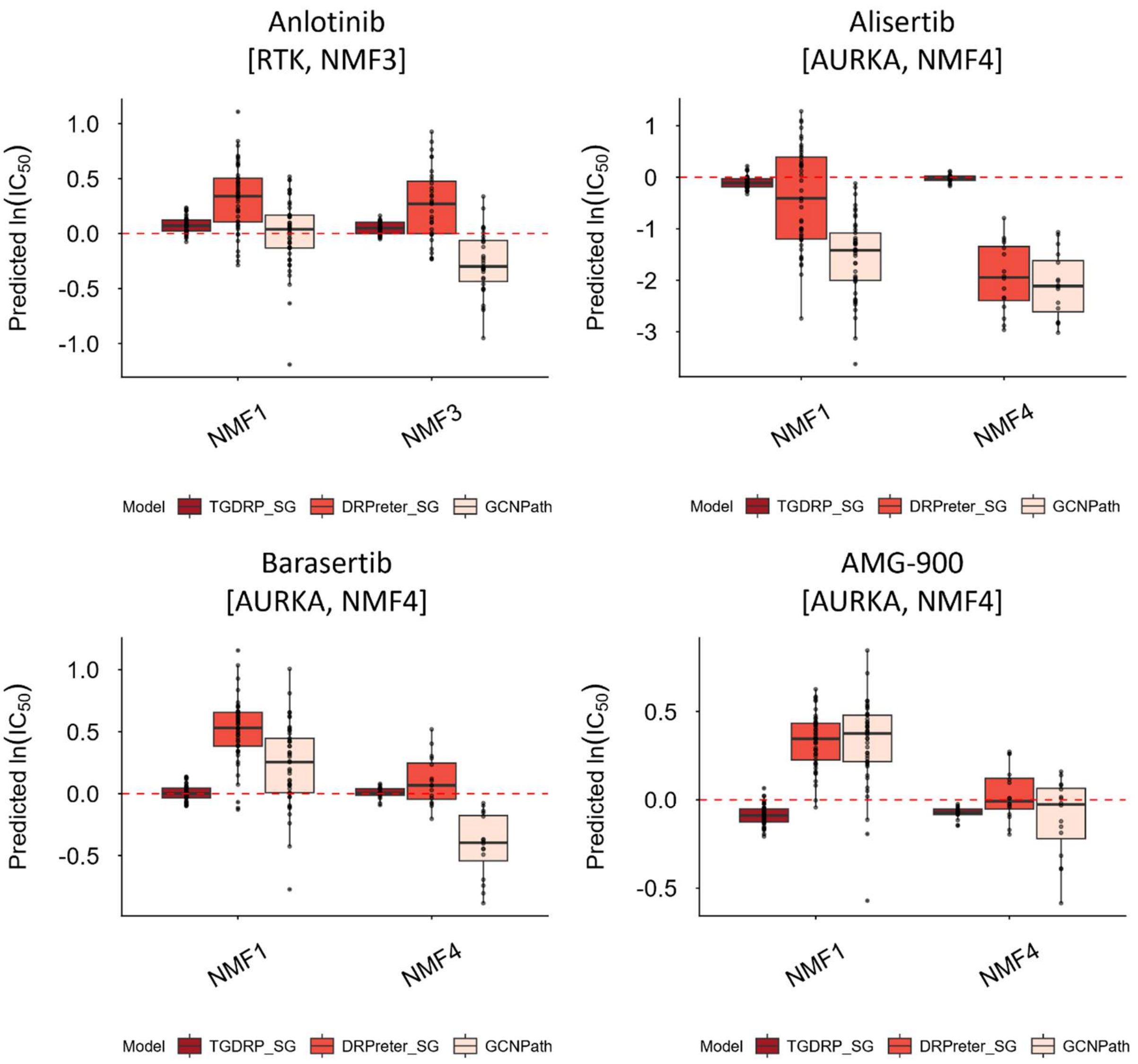

Supplementary Figure 37. Application of GCNPath, TGDRP_SG, and DRPreter_SG in SCLC with clinical data. All GCNPath, TGDRP_SG, DRPreter_SG models trained ten times with the entire GDSC1+2 as a target label dataset were utilized, and prediction values were averaged across models. For the clinical analysis using gene expression data from Liu et al. (2024)[1], drug responses to four compounds were predicted across tumor and matched-normal samples from patients classified as NMF1, NMF3, and NMF4 (n=45, 31 and 16, respectively). The gene expression data utilized for each DRP model were processed with ComBat batch correction using SANGER Cell Model Passports as the reference and cancer/normal status as the matching variable. Known targets and optimal subtypes for each drug are summarized. The source data are provided in Supplementary Data 42.

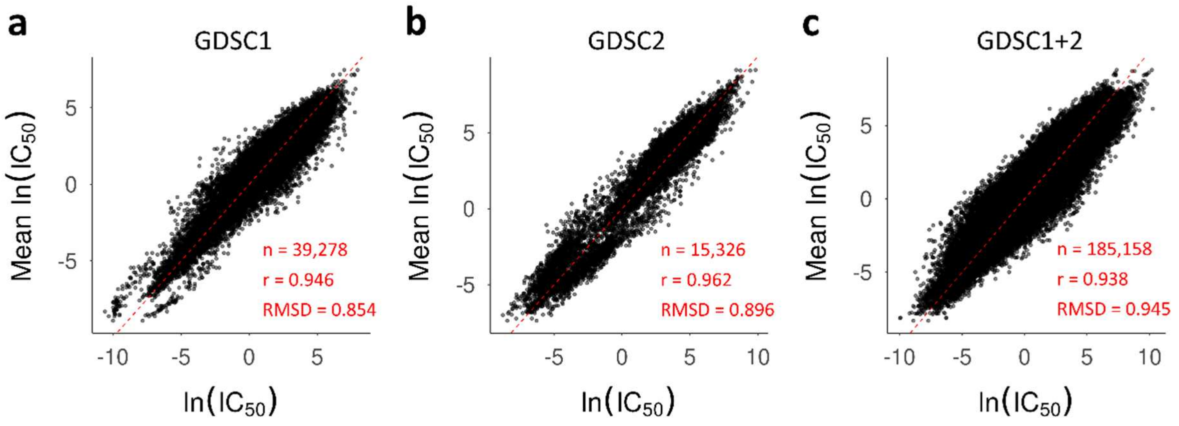

Supplementary Figure 38. Scatter plots of multiple ln(IC_{50}) values for each cell line and drug combination and their respective average values in GDSC datasets. Scatter plots were generated to visualize the IC_{50} values in (**a**) GDSC1 (n=39,278), (**b**) GDSC2 (n=15,326), and (**c**) GDSC1+2 (n=185,158). The number of duplicated ln(IC_{50}) values (n), PCC (r) and root mean square deviation (RMSD) are indicated in each scatter plot using red text. The source data are provided in Supplementary Data 46.

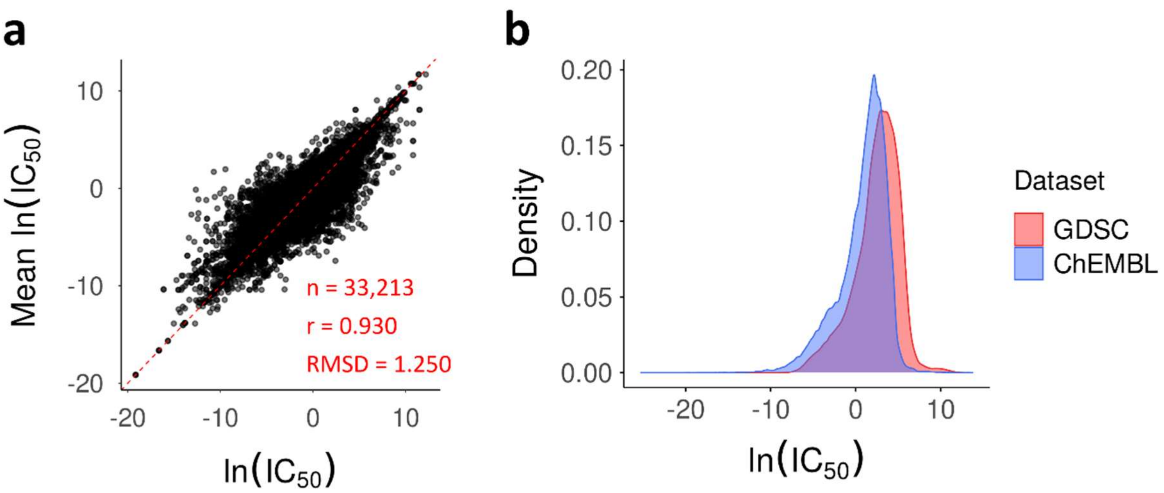

Supplementary Figure 39. The ln(IC_{50}) distribution in ChEMBL dataset. (**a**) Scatter plots of multiple ln(IC_{50}) values for each cell line and drug combination and their respective average values in the ChEMBL dataset (n=33,213). (**b**) The distribution of ln(IC_{50}) values from the ChEMBL and GDSC1+2 datasets. For (**b**), the number of duplicated ln(IC_{50}) values (n), PCC (r) and RMSD are indicated in the scatter plot using red text. The source data are provided in Supplementary Data 50.

Supplementary Tables

Supplementary Table 1. The ability of various deep learning models to predict anticancer drug sensitivity.

Model	Cell Module	Drug Module	Cell-Drug Interaction	Description	Ref
tCNNS [2019]	1D-CNN	1D-CNN	X	Utilizes twin 1D-CNNs to process atom one-hot vectors for drugs and mutations and CNVs for cell lines, respectively.	2
PaccMann [2019]	Attention	Attention	Attention	Utilizes attention modules to detect drug–gene interactions.	3
HiDRA [2021]	Attention	Attention	Attention	Utilizes attention modules to detect both drug–pathway and drug–gene interactions.	4
GraphDRP [2022]	1D-CNN	GCN [GIN]	X	Utilizes a GCN to process drug structures as atom–bond graphs.	5
TGDRP [2022]	GCN [GAT]	GCN [GIN]	X	Utilizes GCNs to integrate cell line mutations, gene expression data and CNVs with a protein–protein interaction network.	6
TGSA [2022]	FCN	FCN	X	Leverages similarities of drug structures and cell line gene expressions to enhance the predictions of TGDRP.	6
DRPreter [2022]	GCN [GAT]	GCN [GIN]	Attention	Utilizes GCNs to retrieve pathway information from each pathway subnetwork and cell line gene expressions and attention module to detect drug–pathway interactions.	7
DRPreter SA [2022]	FCN	FCN	X	Leverages similarities of drug structures and cell line gene expressions to enhance the predictions of DRPreter.	7
**GCNPath [Proposed]**	GCN [RGCN]	GCN [GAT]	X	Integrates pathway-based feature reduction and GCNs to process cell line transcriptomic data.	-
MOLI [2019]	FCN	X	X	Utilizes cell line mutations, transcriptomics and CNVs. Does not adopt a drug information processing module, making this model inadequate for predicting drug responses to unseen chemicals.	8
PathDSP [2021]	FCN	FCN	X	Utilizes pathway-based feature reduction of cell line mutations, transcriptomics and CNVs. Requires drug target information, so cannot make predictions for drugs without any known targets.	9
AGMI [2021]	GCN, nGRN, gGRN	GCN [GIN]	X	Utilizes GCNs to integrate cell line mutations, transcriptomics and CNVs with multiple gene interaction networks. While the GitHub repository includes the model architecture, the code for drug preprocessing was not found, making it unclear which specific drug features were used.	10
Precily [2022]	FCN	FCN	X	Utilizes pathway-based feature reduction of cell line transcriptomics. Requires a time-consuming tuning process using the Hyperband algorithm for training from scratch.	11
iBT-Net [2023]	FCN	Attention [Transformer]	RNN [LSTM]	Utilizes a transformer to represent the interactions between drug atoms and bonds. Introduces a broad learning system and incremental module for the prediction of external	12

			clinical data without time-consuming fine-tuning processes. The GitHub repository for the model architecture and preprocessing data was not found.

1D-CNN: one-dimensional convolution neural network, GCN: graph convolution network, GIN: graph isomorphism network, GAT: graph attention network, FCN: fully connected network, UGCN: uniform GCN, GRU: gated recurrent unit, nGRN: node-level GRU, gGRU: graph-level GRU, RNN: recurrent neural network, LSTM: long short-term memory

* For models listed below the middle boundary line of the table, the reasons for their exclusion from the benchmark are detailed in the Description column.

Supplementary Table 2. Basic topological information of the PCN graphs.

Network	Number of Nodes	Number of Edges	Degree (Mean)	Degree (Min)	Degree (Max)	Path length (Mean)	Path length (Min)	Path length (Max)	Number of Components
STRING_700	292	1460	10	5	31	3.826719	1	8	1
STRING_900	292	1460	10	5	32	3.829261	1	8	1
RegNetwork	292	1460	10	5	104	2.824742	1	5	1
GSVA correlation	292	1460	10	5	24	4.079626	1	8	1
Random edges	292	1460	10	5	21	2.711717	1	4	1

* In each PCN, 292 BIOCARTA pathways were utilized.

* STRING_700 and STRING_900: protein–protein interactions with confidence scores equal to or greater than 0.7 and 0.9, respectively.

Supplementary Table 3. The number of cell lines, drugs, and ln(IC_{50}) values utilized for training and testing each model.

Model	GDSC1+2			GDSC1			GDSC2			Omics Type	Omics Source Database	Parameters
	Cell	Drug	ln(IC_{50})	Cell	Drug	ln(IC_{50})	Cell	Drug	ln(IC_{50})			
BMTMKL	808	432	312,778	803	321	223,615	806	236	167,269	MUT+CNV Proteome + EXP [TPM] + EXP [Microarray] +	GDSC	-
RF	972	432	371,751	965	321	265,448	963	236	198,551	EXP [TPM]	GDSC	-
tCNNS	969	432	371,803	968	321	266,076	961	236	198,275	MUT + CNV	GDSC	3,322,809
HiDRA	947	432	364,450	942	321	260,594	941	236	194,576	EXP [Microarray]	GDSC	1,937,764
PaccMann	395	432	150,204	391	321	107,879	389	236	79,704	EXP [TPM]	GSDC	48,252,129
PaccMann_SG	972	432	371,751	965	321	265,448	963	236	198,551	EXP [TPM]	SANGER	47,494,169
GraphDRP	969	432	371,803	968	321	266,076	961	236	198,275	MUT + CNV	GDSC	873,761
TGDRP	700	432	269,519	696	321	191,356	697	236	144,744	EXP [TPM] + MUT + CNV	CCLE	4,110,689
TGSA	700	432	269,519	696	321	191,356	697	236	144,744	EXP [TPM] + MUT + CNV	CCLE	10,998,273
TGDRP_SG	965	432	369,051	958	321	263,502	956	236	197,114	EXP [TPM] + MUT + CNV	SANGER	4,110,689
TGSA_SG	965	432	369,051	958	321	263,502	956	236	197,114	EXP [TPM] + MUT + CNV	SANGER	10,998,273
DRPreter	696	432	267,965	692	321	190,164	693	236	143,981	EXP [TPM]	CCLE	4,513,617
DRPreter_SA	696	432	267,965	692	321	190,164	693	236	143,981	EXP [TPM]	CCLE	115,790,337
DRPreter_SG	972	432	371,751	965	321	265,448	963	236	198,551	EXP [TPM]	SANGER	4,513,617
DRPreter_SA_SG	972	432	371,751	965	321	265,448	963	236	198,551	EXP [TPM]	SANGER	115,790,337
GCNPath	972	432	371,751	965	321	265,448	963	236	198,551	EXP [TPM]	SANGER	2,549,489

* SANGER: SANGER Cell Model Passports.

* SG: model trained using cell line data from SANGER Cell Model Passports as cell-line input data.

* DRPreter_SA: DRPreter with a similarity augmentation module.

Supplementary Table 4. RMSEs of the models tested with the GDSC datasets.

Test Type	Unblinded Test			Cell-Blind Test			Drug-Blind Test			Strict-Blind Test		
**Dataset**	**GDSC1+2**	**GDSC1**	**GDSC2**	**GDSC1+2**	**GDSC1**	**GDSC2**	**GDSC1+2**	**GDSC1**	**GDSC2**	**GDSC1+2**	**GDSC1**	**GDSC2**
BMTMKL	1.144±0.010	1.170±0.004	1.230±0.009	**1.143±0.030**	**1.170±0.023**	**1.231±0.038**	-	-	-	-	-	-
RF	1.127±0.009	1.154±0.009	1.174±0.008	1.170±0.022	1.190±0.029	1.269±0.027	3.006±0.434	2.882±0.306	2.848±0.483	2.963±0.172	3.194±0.417	2.991±0.311
tCNNS	1.022±0.032[#]	1.050±0.040	1.060±0.021[#]	1.438±0.056	1.419±0.046	1.590±0.045	2.469±0.230[#]	2.509±0.305	2.551±0.562[#]	2.635±0.134[#]	2.648±0.200	2.683±0.232
HiDRA	0.991±0.011	1.019±0.009	1.028±0.010	1.242±0.023	1.260±0.031	1.345±0.057	**2.370±0.218**	2.506±0.212	2.394±0.493	2.548±0.102	2.615±0.152	2.613±0.259
PaccMann	1.092±0.028	1.123±0.095	1.056±0.019	1.285±0.044	1.313±0.070	1.365±0.072	2.435±0.267	2.522±0.342	2.355±0.498	2.584±0.152	2.696±0.173	2.611±0.326
PaccMann_SG	1.045±0.037	1.155±0.280	1.023±0.022	1.248±0.034	1.277±0.025	1.340±0.044	2.430±0.271	2.497±0.319	2.381±0.515	2.552±0.133	2.651±0.169	2.602±0.287
GraphDRP	1.201±0.042	1.155±0.070	1.219±0.038	1.455±0.056	1.437±0.046	1.621±0.057	2.616±0.267	2.687±0.425	2.680±0.441	2.782±0.154	2.817±0.227	2.890±0.233
TGDRP	0.862±0.008	0.862±0.006	0.906±0.012	1.240±0.038	1.250±0.025	1.346±0.052	2.418±0.256	2.495±0.339	2.404±0.539	2.548±0.135	2.585±0.253	2.583±0.324
TGSA	0.861±0.007	0.858±0.007	0.900±0.011	1.255±0.033	1.258±0.033	1.368±0.048	2.418±0.258	2.479±0.334	2.406±0.550	2.549±0.126	2.579±0.248	2.583±0.326
TGDRP_SG	0.847±0.009	0.854±0.005	0.886±0.010	1.226±0.026	1.235±0.034	1.333±0.038	2.440±0.223	2.463±0.290	2.428±0.579	2.549±0.109	2.583±0.232	2.598±0.310
TGSA_SG	0.845±0.008	0.850±0.005	**0.880±0.009**	1.223±0.038	1.236±0.040	1.336±0.034	2.440±0.220	**2.454±0.287**	2.423±0.570	2.552±0.111	2.587±0.236	2.589±0.326
DRPreter	0.866±0.011	0.881±0.009	0.923±0.011	1.233±0.030	1.249±0.042	1.350±0.036	2.434±0.256	2.516±0.315	2.428±0.492	2.532±0.139	**2.564±0.151**	2.567±0.317
DRPreter_SA	0.876±0.011	0.890±0.007	0.926±0.009	1.245±0.041	1.265±0.048	1.371±0.055	2.413±0.243	2.519±0.314	2.433±0.534	2.549±0.129	2.585±0.151	2.553±0.316
DRPreter_SG	0.849±0.006	0.862±0.006	0.897±0.008	1.225±0.024	1.241±0.023	1.318±0.024	2.465±0.255	2.588±0.271	2.369±0.509	2.525±0.111	2.569±0.168	2.545±0.279
DRPreter_SA_SG	0.865±0.010	0.873±0.012	0.902±0.007	1.234±0.024	1.245±0.032	1.352±0.040	2.461±0.228	2.566±0.251	2.391±0.506	2.542±0.119	2.587±0.177	**2.524±0.288**
**GCNPath**	**0.838±0.010**	**0.840±0.008**	0.883±0.014	1.238±0.027	1.266±0.027	1.347±0.027	2.409±0.288	2.463±0.239	**2.353±0.383**	**2.513±0.121**	**2.564±0.135**	2.539±0.193

* Each model was trained and tested with GDSC as target label datasets with 10-fold outer cross-validation in unblinded, cell-blind and drug-blind tests and 25-fold outer cross-validation in strict-blind tests, which resulted in 10 RMSEs and 25 RMSEs for each GDSC dataset, respectively. The performance of each model is indicated as the average plus or minus the standard deviation, and the best performance in each test is represented in boldface. The source data are provided in Supplementary Data 9.

* SG: model trained using cell line data from SANGER Cell Model Passports as cell-line input data.

* DRPreter_SA: DRPreter with a similarity augmentation module.

[#] Unexpectedly, tCNNS produced values of infinity in 6 of the 165 tests: the unblinded test in GDSC2 (n=1), the drug-blind test in GDSC2 (n=2), the unblinded test in GDSC1+2 (n=1), the drug-blind test in GDSC1+2 (n=1), and the strict-blind test in GDSC1+2 (n=1). These outliers were excluded from the calculations.

Supplementary Table 5. PCCs of the models tested with the GDSC datasets.

Test Type	Unblinded Test			Cell-Blind Test			Drug-Blind Test			Strict-Blind Test		
**Dataset**	**GDSC1+2**	**GDSC1**	**GDSC2**	**GDSC1+2**	**GDSC1**	**GDSC2**	**GDSC1+2**	**GDSC1**	**GDSC2**	**GDSC1+2**	**GDSC1**	**GDSC2**
BMTMKL	0.906±0.002	0.896±0.001	0.901±0.002	**0.906±0.005**	**0.896±0.005**	**0.901±0.006**	-	-	-	-	-	-
RF	0.909±0.002	0.899±0.001	0.910±0.002	0.901±0.004	0.893±0.004	0.894±0.003	0.199±0.186	0.206±0.116	0.383±0.115	0.230±0.087	0.028±0.145	0.265±0.123
tCNNS	0.934±0.002#	0.926±0.005	0.934±0.001#	0.856±0.008	0.847±0.011	0.832±0.007	0.389±0.075#	0.319±0.124	0.467±0.068#	0.267±0.048#	0.174±0.075	0.349±0.076
HiDRA	0.931±0.002	0.923±0.001	0.932±0.001	0.889±0.004	0.880±0.005	0.881±0.010	**0.488±0.075**	0.368±0.120	0.527±0.081	0.371±0.032	0.253±0.046	0.413±0.087
PaccMann	0.916±0.004	0.907±0.014	0.928±0.004	0.884±0.008	0.874±0.011	0.879±0.012	0.461±0.112	0.370±0.187	0.537±0.117	0.398±0.075	0.287±0.062	0.451±0.073
PaccMann_SG	0.923±0.007	0.901±0.045	0.933±0.003	0.888±0.007	0.878±0.004	0.882±0.008	0.458±0.118	0.373±0.174	0.531±0.087	0.406±0.075	0.298±0.058	0.458±0.081
GraphDRP	0.907±0.005	0.911±0.010	0.913±0.004	0.849±0.005	0.843±0.011	0.826±0.011	0.377±0.096	0.232±0.188	0.374±0.136	0.268±0.079	0.200±0.112	0.299±0.084
TGDRP	0.948±0.001	0.945±0.001	0.947±0.002	0.889±0.005	0.881±0.004	0.881±0.010	0.464±0.115	0.364±0.165	0.487±0.210	0.384±0.084	0.294±0.085	0.439±0.120
TGSA	0.948±0.001	0.946±0.001	0.948±0.002	0.886±0.006	0.879±0.007	0.876±0.009	0.468±0.116	0.370±0.158	0.491±0.210	0.381±0.081	0.300±0.080	0.436±0.119
TGDRP_SG	0.950±0.001	0.946±0.001	0.950±0.001	0.891±0.004	0.885±0.006	0.883±0.007	0.459±0.096	0.378±0.167	0.490±0.162	0.387±0.072	0.300±0.089	0.432±0.107
TGSA_SG	0.950±0.001	0.947±0.001	**0.951±0.001**	0.892±0.006	0.884±0.007	0.882±0.007	0.459±0.093	0.383±0.160	0.495±0.160	0.387±0.073	0.301±0.085	0.433±0.113
DRPreter	0.947±0.002	0.943±0.001	0.945±0.001	0.891±0.005	0.881±0.008	0.880±0.007	0.479±0.091	0.377±0.164	0.485±0.190	0.398±0.093	0.334±0.057	0.449±0.092
DRPreter_SA	0.946±0.002	0.941±0.001	0.945±0.001	0.888±0.008	0.878±0.010	0.876±0.011	0.483±0.080	0.360±0.162	0.483±0.178	0.380±0.102	0.318±0.052	0.453±0.074
DRPreter_SG	0.949±0.001	0.945±0.001	0.949±0.001	0.892±0.003	0.883±0.004	0.886±0.004	0.456±0.126	0.348±0.162	0.528±0.128	0.412±0.085	0.328±0.047	0.466±0.073
DRPreter_SA_SG	0.947±0.001	0.944±0.001	0.948±0.001	0.890±0.003	0.883±0.005	0.879±0.006	0.457±0.116	0.324±0.141	0.510±0.133	0.393±0.091	0.313±0.039	**0.468±0.062**
**GCNPath**	**0.951±0.002**	**0.948±0.001**	0.950±0.002	0.889±0.005	0.878±0.004	0.880±0.005	0.486±0.117	**0.405±0.143**	**0.538±0.110**	**0.420±0.055**	**0.343±0.048**	0.464±0.071

* Each model was trained and tested with GDSC as target label datasets with 10-fold outer cross-validation in unblinded, cell-blind and drug-blind tests and 25-fold outer cross-validation in strict-blind tests, which resulted in 10 PCCs and 25 PCCs in each GDSC dataset. The performance of each model is indicated as the average plus or minus the standard deviation, and the best performance in each test is represented in boldface. The source data are provided in Supplementary Data 10.

* SG: model trained using cell line data from SANGER Cell Model Passports as cell-line input data.

* DRPreter_SA: DRPreter with a similarity augmentation module.

# Unexpectedly, tCNNS produced values of infinity in 6 of the 165 tests: the unblinded test in GDSC2 (n=1), the drug-blind test in GDSC2 (n=2), the unblinded test in GDSC1+2 (n=1), the drug-blind test in GDSC1+2 (n=1), and the strict-blind test in GDSC1+2 (n=1). These outliers were excluded from the calculations.

Supplementary Table 6. SCCs of the models tested with the GDSC datasets.

Test Type	Unblinded Test			Cell-Blind Test			Drug-Blind Test			Strict-Blind Test		
**Dataset**	**GDSC1+2**	**GDSC1**	**GDSC2**	**GDSC1+2**	**GDSC1**	**GDSC2**	**GDSC1+2**	**GDSC1**	**GDSC2**	**GDSC1+2**	**GDSC1**	**GDSC2**
BMTMKL	0.876±0.001	0.871±0.002	0.862±0.002	**0.876±0.006**	**0.871±0.006**	**0.863±0.008**	-	-	-	-	-	-
RF	0.880±0.003	0.875±0.002	0.875±0.003	0.870±0.005	0.867±0.005	0.853±0.005	0.197±0.175	0.183±0.100	0.368±0.077	0.221±0.102	0.057±0.147	0.238±0.111
tCNNS	0.914±0.003#	0.910±0.006	0.909±0.002#	0.815±0.010	0.814±0.013	0.775±0.009	0.342±0.074#	0.300±0.085	0.445±0.072#	0.230±0.038#	0.170±0.072	0.270±0.077
HiDRA	0.907±0.003	0.903±0.002	0.902±0.002	0.854±0.004	0.850±0.006	0.835±0.012	**0.445±0.072**	0.337±0.100	0.486±0.059	0.332±0.031	0.226±0.044	0.348±0.059
PaccMann	0.890±0.005	0.890±0.006	0.895±0.004	0.849±0.012	0.848±0.006	0.832±0.015	0.414±0.092	0.356±0.138	**0.501±0.104**	0.363±0.053	0.249±0.060	0.399±0.049
PaccMann_SG	0.903±0.003	0.900±0.006	0.905±0.003	0.856±0.007	0.851±0.006	0.839±0.008	0.425±0.105	0.346±0.125	0.500±0.084	**0.369±0.055**	0.255±0.064	**0.413±0.055**
GraphDRP*	0.880±0.005	0.891±0.011	0.881±0.005	0.808±0.007	0.809±0.013	0.768±0.014	0.328±0.087	0.205±0.152	0.334±0.132	0.220±0.067	0.166±0.094	0.209±0.087
TGDRP	0.929±0.002	0.930±0.001	0.924±0.002	0.855±0.008	0.853±0.005	0.837±0.012	0.382±0.105	0.324±0.137	0.438±0.116	0.313±0.069	0.249±0.056	0.323±0.074
TGSA	0.929±0.002	0.930±0.001	0.925±0.002	0.851±0.008	0.851±0.008	0.830±0.011	0.385±0.109	0.331±0.129	0.441±0.114	0.312±0.065	0.253±0.049	0.322±0.072
TGDRP_SG	0.931±0.002	0.931±0.001	0.926±0.002	0.858±0.005	0.857±0.006	0.839±0.009	0.386±0.113	0.334±0.141	0.418±0.105	0.311±0.060	0.261±0.067	0.311±0.065
TGSA_SG	0.931±0.002	0.932±0.001	**0.927±0.002**	0.858±0.008	0.857±0.007	0.837±0.008	0.386±0.113	0.338±0.137	0.425±0.101	0.313±0.060	0.261±0.063	0.318±0.065
DRPreter	0.928±0.002	0.927±0.002	0.921±0.002	0.858±0.007	0.854±0.010	0.837±0.010	0.414±0.096	0.326±0.117	0.413±0.130	0.335±0.079	0.277±0.061	0.351±0.060
DRPreter_SA	0.927±0.002	0.926±0.002	0.921±0.002	0.854±0.010	0.849±0.011	0.834±0.014	0.413±0.080	0.310±0.136	0.413±0.130	0.318±0.091	0.268±0.061	0.351±0.050
DRPreter_SG	0.931±0.001	0.930±0.001	0.925±0.002	0.859±0.005	0.856±0.004	0.843±0.007	0.376±0.124	0.323±0.128	0.465±0.091	0.341±0.082	0.273±0.041	0.355±0.044
DRPreter_SA_SG	0.929±0.002	0.928±0.002	0.924±0.002	0.857±0.005	0.855±0.006	0.835±0.007	0.371±0.118	0.285±0.120	0.450±0.096	0.321±0.089	0.263±0.041	0.358±0.040
**GCNPath**	**0.933±0.002**	**0.933±0.002**	**0.927±0.003**	0.855±0.008	0.849±0.006	0.835±0.008	0.417±0.119	**0.373±0.109**	0.470±0.094	0.355±0.040	**0.292±0.061**	0.373±0.052

* Each model was trained and tested with GDSC as target label datasets with 10-fold outer cross-validation in unblinded, cell-blind and drug-blind tests and 25-fold outer cross-validation in strict-blind tests, which resulted in 10 SCCs and 25 SCCs in each GDSC dataset. The performance of each model is indicated as the average plus or minus the standard deviation, and the best performance in each test is represented in boldface. The source data are provided in Supplementary Data 11.

* SG: model trained using cell line data from SANGER Cell Model Passports as cell-line input data.

* DRPreter_SA: DRPreter with similarity augmentation module.

# Unexpectedly, tCNNS produced values of infinity in 6 of the 165 tests: the unblinded test in GDSC2 (n=1), the drug-blind test in GDSC2 (n=2), the unblinded test in GDSC1+2 (n=1), the drug-blind test in GDSC1+2 (n=1), and the strict-blind test in GDSC1+2 (n=1). These outliers were excluded from the calculations.

Supplementary Table 7. Performance of the models pretrained by the respective developers with the GDSC datasets.

Model	Dataset	Num of ln(IC_{50})	RMSE	PCC	SCC
PaccMann_MSE	GDSC1+2	150204	2.074	0.642	0.629
PaccMann_MSE	GDSC1	107879	1.882	0.710	0.705
PaccMann_MSE	GDSC2	79704	2.390	0.561	0.527
PaccMann_PCC	GDSC1+2	150204	2.089	0.641	0.626
PaccMann_PCC	GDSC1	107879	1.903	0.700	0.694
PaccMann_PCC	GDSC2	79704	2.403	0.571	0.536
GraphDRP	GDSC1+2	371803	2.074	0.679	0.620
GraphDRP	GDSC1	266076	1.558	0.807	0.753
GraphDRP	GDSC2	198275	2.560	0.603	0.516
DRPreter	GDSC1+2	267965	2.261±0.043	0.610±0.014	0.562±0.014
DRPreter	GDSC1	190164	2.564±0.044	0.495±0.016	0.408±0.015
DRPreter	GDSC2	143981	1.677±0.083	0.815±0.018	0.784±0.017
TGDRP	GDSC1+2	269519	2.447	0.510	0.444
TGDRP	GDSC1	191356	2.612	0.448	0.339
TGDRP	GDSC2	144744	2.135	0.667	0.616
TGDRP_Pre	GDSC1+2	269519	2.404	0.501	0.417
TGDRP_Pre	GDSC1	191356	2.502	0.438	0.319
TGDRP_Pre	GDSC2	144744	2.226	0.651	0.568
TGSA	GDSC1+2	269519	2.386	0.497	0.414
TGSA	GDSC1	191356	2.478	0.451	0.331
TGSA	GDSC2	144744	2.189	0.635	0.554
TGSA_Pre	GDSC1+2	269519	2.408	0.526	0.467
TGSA_Pre	GDSC1	191356	2.554	0.461	0.370
TGSA_Pre	GDSC2	144744	2.011	0.723	0.673

* Source data are provided in Supplementary Data 22.

* PaccMann_MSE, PaccMann_PCC: PaccMann pretrained by minimizing the MSE loss or maximizing the PCC, respectively.

* DRPreter models were pretrained with ten random seeds (2, 16, 33, 61, 79, 100, 220, 653, 1004, and 4001), so the performance is indicated as the average plus or minus the standard deviation.

* TGDRP_Pre, TGSA_Pre: TGDRP and TGSA with self-supervised learning of the GCN drug module with the ZINC15 and ChEMBL datasets, respectively.

Supplementary Table 8. Performance of the models with the ChEMBL dataset.

Model	RMSE	PCC	SCC
RF	3.585±0.028	0.150±0.003	0.132±0.002
tCNNS#	47.874±8.705	0.002±0.041	-0.005±0.044
HiDRA	**3.402±0.029**	**0.188±0.006**	0.155±0.007
PaccMann_SG	16.976±38.204	0.050±0.039	0.054±0.044
GraphDRP	3.638±0.110	0.089±0.037	0.098±0.026
TGDRP	3.671±0.038	0.164±0.020	0.148±0.017
TGSA	3.693±0.040	0.167±0.015	0.150±0.016
TGDRP_SG	3.718±0.053	0.168±0.010	0.151±0.010
TGSA_SG	3.718±0.034	0.168±0.012	0.152±0.010
DRPreter	3.629±0.045	0.165±0.018	0.149±0.019
DRPreter_SG	3.624±0.061	0.159±0.017	0.143±0.016
GCNPath	3.453±0.052	0.179±0.006	**0.162±0.006**

* Each model was trained ten times with the entire GDSC1+2 and tested using ChEMBL as target label datasets, which resulted in 10 RMSEs, PCCs, and SCCs for each model. The performance is indicated as the average plus or minus the standard deviation, and the best performance is represented in boldface. The source data are provided in Supplementary Data 28.

* SG: model trained using cell line data from SANGER Cell Model Passports as cell-line input data.

* PaccMann trained with TPM data of 457 cell lines from GDSC was excluded because it can predict only 60,539 ln(IC_{50}) values.

* DRPreter models with a similarity augmentation module (DRPreter_SA, DRPreter_SA_SG) were excluded because they cannot predict the responses of drugs not included during the training phase.

# tCNNS models output values of infinity for 384,027 of a total of 2,375,800 ln(IC_{50}) values. These outliers were excluded from the calculations.

Supplementary Table 9. Performance of the models trained with the ChEMBL dataset or external cell line RNA datasets.

Model	Gene Expression Training Dataset	Gene Expression Testing Dataset	RMSE	PCC	SCC
		GDSC	3.698±0.026	0.122±0.003	0.100±0.003
RF	SANGER	CCLE	3.608±0.025	0.143±0.003	0.124±0.003
		SANGER	3.585±0.028	0.150±0.003	0.132±0.002
		GDSC	**3.402±0.029**	**0.188±0.006**	**0.155±0.007**
HiDRA	GDSC	CCLE	5.012±1.218	0.132±0.023	0.130±0.026
		SANGER	4.700±1.134	0.127±0.025	0.125±0.026
		GDSC	24.315±35.615	0.035±0.021	0.034±0.023
PaccMann_SG	SANGER	CCLE	14.027±28.882	0.056±0.041	0.063±0.044
		SANGER	16.976±38.204	0.050±0.039	0.054±0.044
		GDSC	3.763±0.050	0.138±0.019	0.133±0.016
TGDRP	CCLE	CCLE	3.671±0.038	0.164±0.020	0.148±0.017
		SANGER	3.745±0.043	0.143±0.017	0.137±0.014
		GDSC	3.781±0.044	0.128±0.016	0.122±0.015
TGSA	CCLE	CCLE	3.693±0.040	0.167±0.015	0.150±0.016
		SANGER	3.759±0.026	0.132±0.016	0.125±0.015
		GDSC	3.810±0.062	0.152±0.013	0.145±0.012
TGDRP_SG	SANGER	CCLE	3.770±0.057	0.161±0.011	0.147±0.011
		SANGER	3.718±0.053	0.168±0.010	0.151±0.010
		GDSC	3.801±0.048	0.148±0.014	0.143±0.013
TGSA_SG	SANGER	CCLE	3.773±0.042	0.155±0.014	0.144±0.012
		SANGER	3.718±0.034	0.168±0.012	0.152±0.010
		GDSC	3.669±0.058	0.148±0.017	0.136±0.019
DRPreter	CCLE	CCLE	3.629±0.045	0.165±0.018	0.149±0.019
		SANGER	3.667±0.042	0.159±0.016	0.143±0.018
		GDSC	3.662±0.066	0.144±0.020	0.131±0.019
DRPreter_SG	SANGER	CCLE	3.673±0.062	0.156±0.016	0.138±0.014
		SANGER	3.624±0.061	0.159±0.017	0.143±0.016
		GDSC	3.582±0.073	0.155±0.008	0.143±0.009
GCNPath	SANGER	CCLE	**3.546±0.064**	**0.174±0.007**	**0.159±0.008**
		SANGER	**3.453±0.052**	**0.179±0.006**	**0.162±0.006**

* Each model was trained ten times with the entire GDSC1+2 dataset and tested using ChEMBL as target label datasets, which resulted in 10 RMSEs, PCCs, and SCCs for each model. The performance is indicated as the average plus or minus the standard deviation, and the best performance is represented in boldface. The source data are provided in Supplementary Data 28.

* SG: model trained using cell line data from SANGER Cell Model Passports as cell-line input data.

* PaccMann trained with TPM data of 457 cell lines from GDSC was excluded because it can predict only 60,539 $\ln(IC_{50})$ values with those cell lines.

* DRPreter models with a similarity augmentation module (DRPreter_SA, DRPreter_SA_SG) were excluded because they cannot predict the responses of drugs not included during the training phase.

* tCNNS and GraphDRP were excluded because they do not utilize RNA expression data.

Supplementary Table 10. Preprocessing methods of gene expression data for each DRP model.

**Models**	**TPM from SANGER Cell Model Passports and CCLE**	**Microarray from GDSC (RMA-normalized in default)**
RF	-	-
HiDRA	Log_2(TPM+1) plus<br>Sample-wise standardization	Sample-wise standardization
PaccMann_SG	ArcSinh normalization<br>(Instead of Log_2(TPM+1))	(None)
TGDRP, TGSA,<br>TGDRP_SG, TGSA_SG,<br>DRPreter, DRPreter_SG	Log_2 (TPM+1) plus<br>Gene-wise standardization#	Gene-wise standardization#
GCNPath	Log_2(TPM+1) plus<br>GSVA plus<br>Pathway-wise standardization	GSVA plus<br>Pathway-wise standardization

Gene-wise standardization was performed using the mean and standard deviation of each gene for each dataset, rather than transforming external datasets using the values derived from the training dataset.

Supplementary Table 11. Top 5 pathways determinant for drug sensitivity or resistance, detected by Grad-CAM in GCNPath.

Cell	Drug	Prediction	Pathway
LB647-SCLC	Etoposide	0.768	BIOCARTA_P38MAPK_PATHWAY
			BIOCARTA_LIS1_PATHWAY
			BIOCARTA_SARS_PATHWAY
			BIOCARTA_PROTEASOME_PATHWAY
			BIOCARTA_MET_PATHWAY
NCI-H748	Cisplatin	1.571	BIOCARTA_IFNG_PATHWAY
			BIOCARTA_MET_PATHWAY
			BIOCARTA_IL22BP_PATHWAY
			BIOCARTA_EOSINOPHILS_PATHWAY
			BIOCARTA_CBL_PATHWAY
NCI-H847	Lurbinectedin	-1.095	BIOCARTA_CCR5_PATHWAY
			BIOCARTA_IL12_PATHWAY
			BIOCARTA_CARM_ER_PATHWAY
			BIOCARTA_SARS_PATHWAY
			BIOCARTA_RHODOPSIN_PATHWAY
COR-L279	Pyrrolobenzo diazepine	2.325	BIOCARTA_EPONFKB_PATHWAY
			BIOCARTA_PML_PATHWAY
			BIOCARTA_SARS_PATHWAY
			BIOCARTA_IL12_PATHWAY
			BIOCARTA_STATHMIN_PATHWAY
NCI-H209	Deruxtecan	-2.501	BIOCARTA_CIRCADIAN_PATHWAY
			BIOCARTA_TNFR1_PATHWAY
			BIOCARTA_GABA_PATHWAY
			BIOCARTA_PDZS_PATHWAY
			BIOCARTA_RAC1_PATHWAY
IST-SL1	Calicheamicin	-0.631	BIOCARTA_CREM_PATHWAY
			BIOCARTA_IL2_PATHWAY
			BIOCARTA_RAC1_PATHWAY
			BIOCARTA_EPONFKB_PATHWAY
			BIOCARTA_G1_PATHWAY

Supplementary Table 12. Drug features of atoms and bonds utilized in GCNPath.

Feature Type	Feature	Size
Atom	Atom type	36 [one-hot embedding]
	Atom mass (×0.01)	1
	Atom is in ring	1
	Atom is aromatic	1
	Atom degree	11 [one-hot embedding]
	Number of hydrogens	5 [one-hot embedding]
	Hybridization	5 [one-hot embedding]
	Chirality type	2 [one-hot embedding]
	Formal charge	5 [one-hot embedding]
	Explicit valence	6 [one-hot embedding]
	Implicit valence	7 [one-hot embedding]
	Number of radical electrons	5 [one-hot embedding]
	Total	85
Bond	Bond type	4 [one-hot embedding]
	Bond conjugated	1
	Bond is in ring	1
	Bond stereotype	4 [one-hot embedding]
	Total	10

Supplementary Notes

Supplementary Note 1. Gene and protein networks are successfully compressed into pathway crosstalk network (PCN) graphs

For accurate drug sensitivity prediction with GCNPath, each cell line is represented by multiple pathway crosstalk network (PCN) graphs. These graphs abstract biological pathways as nodes, with edges representing their interactions or overlaps, providing a functional view of cellular mechanisms beyond individual genes and proteins. Constructed using the separation score, a metric that estimates pathway distances by calculating the average shortest distance between genes in networks, these graphs offer a systematic representation of pathway relationships. To assess the reliability of the separation score, we analyzed its correlation with gene overlap ratios among pathway pairs. We hypothesized that higher separation scores would indicate lower gene overlap, reflecting greater pathway dissimilarity. This analysis, performed on PCN graphs from STRING and RegNetwork using 292 BIOCARTA pathways, confirmed that gene overlap declined as separation scores increased (**Supplementary Figure 1a-c; Supplementary Data 1**), validating the metric's effectiveness in measuring pathway distances.

We also explored the topological differences among the PCN graphs by analyzing their pairwise edge overlap ratios. Three types of PCN graphs were constructed: (1) STRING, (2) RegNetwork, and (3) a pathway correlation network based on gene set variation analysis (GSVA) pathway scores. STRING focuses on physical or functional PPIs, RegNetwork on GRNs driven by transcriptional regulation, and the correlation network captures co-expression patterns between pathways. The edge overlap ratios among these networks ranged from 17.13% to 37.81%, except for comparisons with random graphs (1.10–1.44%) and between STRING_700 and STRING_900 (75.89%), the latter sharing significant overlap due to their common origin (**Supplementary Figure 1d; Supplementary Data 1**). This demonstrates that each PCN graph contains distinct pathway crosstalk information. Visualization of the topological features of the PCN graphs further reveals unique pathway connections (**Supplementary Figure 2; Supplementary Table 2; Supplementary Data 2**).

Supplementary Note 2. Discussion and Limitation on treatment response data in TCGA (Part of Methods "Processing of TCGA data")

As for the clinical endpoint, we used treatment response, not survival, for validation on TCGA samples. Treatment response annotations were extracted from TCGA clinical metadata using the TCGAbiolinks R package, which includes structured fields describing patient responses to administered therapies. Some references[13,14] have introduced TCGA response data as following Response Evaluation Criteria in Solid Tumors (RECIST), which evaluates anticancer drug efficacy based on changes in tumor size.

Although the metadata does not explicitly state whether RECIST criteria were applied, the available labels (e.g., complete response, partial response, stable disease, progressive disease) closely mirror RECIST-based terminology, which is the prevailing standard for evaluating solid tumor response in clinical settings. Following prior studies (e.g., Shen et al.[15]), we binarized the response categories to define responders as patients with complete or partial responses (CR, PR), and non-responders as those with stable or progressive disease (SD, PD). This classification enabled a standardized evaluation across multiple cancer types, despite variability in original study protocols.

We recognize that RECIST is not formally applicable to certain tumor types (e.g., hepatocellular carcinoma, brain tumors), as noted in recent oncology guidance (e.g., NIH RECIST guidelines presentation). Although some of these tumor types are included in the TCGA cohorts retrieved via TCGAbiolinks, we attempted to mitigate heterogeneity in endpoint definitions by restricting our analysis to patients with monotherapy records and uniquely defined response labels. This filtering ensured that model evaluation was performed under the most uniform conditions possible given the available data.

Notably, all cancer types included in our analysis were solid tumors, and we did not include any liquid malignancies (e.g., leukemias). Thus, in cases where RECIST criteria were applied, response metrics would reflect changes in solid tumor size, consistent with established clinical practice.

Supplementary Note 3. Preparing omics datasets, training for each model, and exploitation of pretrained models (Part of Methods "Benchmark tests with GDSC datasets")

We summarize the omics datasets (**Supplementary Table 3**) and versions used for training and testing each model. When specific versions were available in the corresponding databases or GitHub repositories, those were used. For example, DRPreter was trained with transcripts per million (TPM) data from CCLE (DepMap 21Q4 version), while GraphDRP and PaccMann used omics data from their respective repositories. tCNNS was trained and tested with the same omics data as GraphDRP, as both rely on identical datasets. For models without documented dataset versions, we used one of the following: (1) TPM data from SANGER Cell Model Passports (v.2.9.0), (2) TPM data from Cancer Cell Line Encyclopedia (CCLE, DepMap 23Q2 version), or (3) microarray data from Genomics of Drug Sensitivity in Cancer (GDSC, Release 8.4). For TGSA, multi-omics data were obtained from CCLE (DepMap 23Q2), including somatic mutations and log2-scaled copy number ratios with a pseudo-count of 1. The same data were also obtained from SANGER Cell Model Passports (v.2.9.0), filtering non-coding mutations and converting copy number ratios to log2 scale with a pseudo-count of 1.

We highlight several considerations in the training and testing process for each model. In blind tests, we acknowledge that DRPreter_SA, DRPreter_SG_SA, and TGSA are indirectly exposed to unseen cell lines and/or drugs through cell-line and drug similarity graphs. Nevertheless, these models were included in the benchmark tests with GDSC datasets to evaluate whether incorporating similarity information can improve performance in blind tests. The similarity graphs were constructed using cell lines and drugs with available gene expression data, drug structure data, and/or corresponding response data. As a result, three pairs of similarity graphs were derived for each model based on GDSC1, GDSC2, and GDSC1+2 datasets. The number of nodes in the similarity graphs corresponded to the number of cell lines and drugs used for $\ln(IC_{50})$ prediction (**Supplementary Table 3**).

Notably, the TGSA implementation in GitHub does not incorporate drug–drug similarities, in contrast to the method described in the original publication, and we adhered to the GitHub version for our experiments. For PaccMann, we utilized version 2 (PaccMannV2), as recommended in its GitHub repository. Notably, the numbers of cell lines utilized in training DRPreter, TGDRP, TGSA and PaccMann were much lower than those used in our model. To minimize the impact of varying training sample sizes, we performed de novo training of these models with SANGER Cell Model Passports data

(**Supplementary Figure 3a; Supplementary Table 3**). For tCNNS and GraphDRP, these models predict the $\ln(IC_{50})$ values transformed into the [0, 1] range with the function $y = 1/(1 + (e^x)^{-0.1})$. We applied this transformation to the actual $\ln(IC_{50})$ values and reversed the transformation to obtain the predicted values.

We also used pretrained models from GitHub, incorporating all available weights, including those with different training conditions, architectures, or strategies. For example, DRPreter was trained with ten random seeds, and PaccMann models were trained to minimize MSE or maximize PCC (PaccMann_MSE and PaccMann_PCC). Additionally, pretrained TGDRP and TGSA models utilizing ZINC15 and ChEMBL datasets were included (TGDRP_Pre and TGSA_Pre). Predictions were made with GDSC1, GDSC2, and GDSC1+2 as target label datasets, and performance metrics (RMSE, Root Mean Square Error; PCC, Pearson Correlation Coefficient; SCC, Spearman Correlation Coefficient) were calculated. Additionally, RMSE values for each cell line and drug ($RMSE_C$ and $RMSE_D$, respectively) derived from the prediction values of GDSC1+2 were also estimated. Lastly, we analyzed similarity patterns in $RMSE_C$ and $RMSE_D$ between pretrained and scratch-trained models by calculating PCCs across unblinded, cell-blind, drug-blind, and strict-blind tests.

Supplementary Note 4. Preparing gene expression datasets and analysis of batch correction for feature preprocessing (Part of Methods "Benchmark tests with ChEMBL dataset")

We summarize the preprocessing methods for gene expression features in each RNA-based model. For GCNPath, GSVA scores from external datasets were standardized using the mean and standard deviation of each pathway, calculated from TPM data in the SANGER Cell Model Passports, following the original procedure. This used the fit_transform function from the scikit-learn package (v.1.2.2)[16]. For gene-based models (e.g., TGDRP, TGSA, DRPreter), differences in data distribution across datasets made transformations based on the training dataset unsuitable (**Fig. 5a; Supplementary Data 33**). Thus, each external dataset was standardized independently using its own mean and standard deviation for each gene. For PaccMann_SG, TPM data from the SANGER Cell Model Passports and CCLE were normalized using the hyperbolic arcsine (arcsinh) transformation instead of log2, as outlined in the GitHub issue (https://github.com/PaccMann/paccmann_predictor/issues/6). This transformation was applied using the asinh function in R (v.4.3.3). Microarray data from GDSC were not arcsine-normalized, as they were already processed using robust multiarray analysis (RMA). HiDRA performed sample-wise standardization for all datasets as described in the GitHub repository.

In addition to ChEMBL predictions, the impact of batch correction via gene- and pathway-based preprocessing was examined using principal component analysis (PCA) plots and boxplots. We selected 1,509 genes from 292 BIOCARTA pathways for each gene expression dataset. Of these, 1,506, 1,498, and 1,410 genes overlapped with data from SANGER Cell Model Passports, CCLE, and GDSC, respectively. Gene-based preprocessing methods included gene- or sample-wise standardization and arcsine normalization (except for GDSC microarray data, which were already normalized). For pathway-based methods, GSVA scores were calculated as described in **Methods ("Processing of cell line data")**. Additionally, other pathway scores were computed using ssGSEA[17], singscore[18], and stingscore[19]. The gsva function from the GSVA R package (v.1.50.5) was used for ssGSEA with the parameter method="ssgsea", and the multiScore function from the singscore package (v1.22.0) for singscore and stingscore calculations.

For ssGSEA, the default parameter option includes normalization step to ensure comparability across samples by adjusting pathway scores, but this can lead to scores influenced by sample distributions, thus failing to produce fully reproducible values. To address this, pathway scores were

calculated without normalization by setting ssgsea.norm=FALSE. For stingscore, gene expression data were mapped to 100 genes with stable expression across cancers, selected using the getStableGenes function (type="carcinoma"). These genes are also included in the TPM data from the SANGER Cell Model Passports and BIOCARTA pathways. Of these, 100 and 93 genes overlapped with CCLE and GDSC datasets, respectively.

Gene expression data were filtered to include only the 404 cell lines used for ChEMBL predictions. PCA plots were generated using 1,408 genes or 292 BIOCARTA pathways shared across all datasets. Pairwise PCC values were calculated from gene and pathway expression data for these 404 cell lines, with PCC distributions compared using boxplots for identical and different cell pairs.

Supplementary References

1. Liu, Q. *et al.* Proteogenomic characterization of small cell lung cancer identifies biological insights and subtype-specific therapeutic strategies. *Cell* **187**, 184-203. e128 (2024).
2. Liu, P., Li, H., Li, S. & Leung, K. S. Improving prediction of phenotypic drug response on cancer cell lines using deep convolutional network. *BMC Bioinformatics* **20**, 408, doi:10.1186/s12859-019-2910-6 (2019).
3. Manica, M. *et al.* Toward Explainable Anticancer Compound Sensitivity Prediction via Multimodal Attention-Based Convolutional Encoders. *Mol Pharm* **16**, 4797-4806, doi:10.1021/acs.molpharmaceut.9b00520 (2019).
4. Jin, I. & Nam, H. HiDRA: Hierarchical Network for Drug Response Prediction with Attention. *J Chem Inf Model* **61**, 3858-3867, doi:10.1021/acs.jcim.1c00706 (2021).
5. Nguyen, T., Nguyen, G. T. T., Nguyen, T. & Le, D. H. Graph Convolutional Networks for Drug Response Prediction. *IEEE/ACM Trans Comput Biol Bioinform* **19**, 146-154, doi:10.1109/TCBB.2021.3060430 (2022).
6. Zhu, Y. *et al.* TGSA: protein-protein association-based twin graph neural networks for drug response prediction with similarity augmentation. *Bioinformatics* **38**, 461-468, doi:10.1093/bioinformatics/btab650 (2022).
7. Shin, J., Piao, Y., Bang, D., Kim, S. & Jo, K. DRPreter: Interpretable Anticancer Drug Response Prediction Using Knowledge-Guided Graph Neural Networks and Transformer. *Int J Mol Sci* **23**, doi:10.3390/ijms232213919 (2022).
8. Sharifi-Noghabi, H., Zolotareva, O., Collins, C. C. & Ester, M. MOLI: multi-omics late integration with deep neural networks for drug response prediction. *Bioinformatics* **35**, i501-i509 (2019).
9. Tang, Y.-C. & Gottlieb, A. Explainable drug sensitivity prediction through cancer pathway enrichment. *Scientific reports* **11**, 3128 (2021).
10. Feng, R. *et al.* in *2021 IEEE International Conference on Bioinformatics and Biomedicine (BIBM).* 1295-1298 (IEEE).
11. Chawla, S. *et al.* Gene expression based inference of cancer drug sensitivity. *Nat Commun* **13**, 5680, doi:10.1038/s41467-022-33291-z (2022).
12. Zhan, Y., Guo, J., Philip Chen, C. & Meng, X.-B. iBT-Net: an incremental broad transformer network for cancer drug response prediction. *Briefings in Bioinformatics* **24**, bbad256 (2023).
13. Ding, Z., Zu, S. & Gu, J. Evaluating the molecule-based prediction of clinical drug responses in cancer. *Bioinformatics* **32**, 2891-2895 (2016).
14. Hostallero, D. E., Wei, L., Wang, L., Cairns, J. & Emad, A. Preclinical-to-clinical anti-cancer drug response prediction and biomarker identification using TINDL. *Genomics, Proteomics & Bioinformatics* **21**, 535-550 (2023).
15. Shen, B. *et al.* A systematic assessment of deep learning methods for drug response prediction: from in vitro to clinical applications. *Brief Bioinform* **24**, doi:10.1093/bib/bbac605

(2023).

16 Pedregosa, F. *et al.* Scikit-learn: Machine learning in Python. *the Journal of machine Learning research* **12**, 2825-2830 (2011).

17 Barbie, D. A. *et al.* Systematic RNA interference reveals that oncogenic KRAS-driven cancers require TBK1. *Nature* **462**, 108-112 (2009).

18 Foroutan, M. *et al.* Single sample scoring of molecular phenotypes. *BMC Bioinformatics* **19**, 404, doi:10.1186/s12859-018-2435-4 (2018).

19 Bhuva, D. D., Cursons, J. & Davis, M. J. Stable gene expression for normalisation and single-sample scoring. *Nucleic Acids Research* **48**, e113-e113 (2020).
