# Supplementary material for: GCNPath: introspecting drug response prediction with pathway-guided graph convolution networks
Source: Commun Biol. 2026 Apr 1;9:720. doi: 10.1038/s42003-026-09957-5 (PMC13212977; doi:10.1038/s42003-026-09957-5)
Supplement: Supplementary file 2 — Reporting Summary [file 42003_2026_9957_MOESM2_ESM.pdf]

nature portfolio

Corresponding author(s): Minho Lee

Last updated by author(s): Jan 2, 2026

Reporting Summary

Nature Portfolio wishes to improve the reproducibility of the work that we publish. This form provides structure for consistency and transparency in reporting. For further information on Nature Portfolio policies, see our Editorial Policies and the Editorial Policy Checklist.

Statistics

For all statistical analyses, confirm that the following items are present in the figure legend, table legend, main text, or Methods section.

n/a	Confirmed	
☐	☒	The exact sample size (*n*) for each experimental group/condition, given as a discrete number and unit of measurement
☒	☐	A statement on whether measurements were taken from distinct samples or whether the same sample was measured repeatedly
☐	☒	The statistical test(s) used AND whether they are one- or two-sided<br>*Only common tests should be described solely by name; describe more complex techniques in the Methods section.*
☒	☐	A description of all covariates tested
☐	☒	A description of any assumptions or corrections, such as tests of normality and adjustment for multiple comparisons
☐	☒	A full description of the statistical parameters including central tendency (e.g. means) or other basic estimates (e.g. regression coefficient) AND variation (e.g. standard deviation) or associated estimates of uncertainty (e.g. confidence intervals)
☐	☒	For null hypothesis testing, the test statistic (e.g. *F*, *t*, *r*) with confidence intervals, effect sizes, degrees of freedom and *P* value noted<br>*Give P values as exact values whenever suitable.*
☒	☐	For Bayesian analysis, information on the choice of priors and Markov chain Monte Carlo settings
☒	☐	For hierarchical and complex designs, identification of the appropriate level for tests and full reporting of outcomes
☐	☒	Estimates of effect sizes (e.g. Cohen's *d*, Pearson's *r*), indicating how they were calculated

Our web collection on statistics for biologists contains articles on many of the points above.

Software and code

Policy information about availability of computer code

Data collection	We utilized the following packages to collect data in code format: webchem R package (v.1.2.0), ChEMBL web resource client python package (version 0.10.8), and TCGABiolinks R package (version 2.25.3). The detailed description was depicted in Methods and Data availability statement. All codes for data collection with those packages are available at the subdirectories of GCNPath2026/_IC50_Prediction/project in the GitHub repository (https://github.com/MinhoLee-DGU/GCNPath2026). All data we downloaded are publicly available.
Data analysis	We utilized the following packages to analyze and process data in code format: GSVA R package (v.1.44.5), igraph R package (v.1.3.5), dnet R package (v.1.1.7), scikit-learn python package (v.1.2.2), dgllife python package (v.0.2.9), Pytorch python package (v.1.11.0), and Pytorch Geometric python package (v.2.1.0). The detailed description was depicted in Methods and Supplementary Information. All codes for data analysis and processing are available at the subdirectories of GCNPath2026/_IC50_Prediction/project in the GitHub repository (https://github.com/MinhoLee-DGU/GCNPath2026).

For manuscripts utilizing custom algorithms or software that are central to the research but not yet described in published literature, software must be made available to editors and reviewers. We strongly encourage code deposition in a community repository (e.g. GitHub). See the Nature Portfolio guidelines for submitting code & software for further information.

Data

Policy information about availability of data

All manuscripts must include a data availability statement. This statement should provide the following information, where applicable:

- Accession codes, unique identifiers, or web links for publicly available datasets
- A description of any restrictions on data availability
- For clinical datasets or third party data, please ensure that the statement adheres to our policy

Cell-line TPM data were obtained from the SANGER Cell Model Passports database (version 2.9.0; https://cog.sanger.ac.uk/cmp/download/rnaseq_all_20220624.zip; "rnaseq_tpm_20220624.csv") and the CCLE DepMap database (versions 21Q4 and 23Q2; https://depmap.org/portal/data_page/?tab=allData&releasename=DepMap%20Public%2021Q4&filename=CCLE_expression_full.csv; https://depmap.org/portal/download/all/?releasename=DepMap+Public+23Q2&filename=OmicsExpressionProteinCodingGenesTPMLogp1.csv). Cell-line microarray data were retrieved from GDSC Release 8.4 (https://www.cancerrxgene.org/gdsc1000/GDSC1000_WebResources/Data/preprocessed/Cell_line_RMA_proc_basalExp.txt.zip). Protein–protein interaction (PPI) and gene regulatory network (GRN) data were obtained from STRING (version 11.5; https://string-db.org/cgi/download?sessionId=btooBqgiAEcP&species_text=9606) and RegNetwork (https://regnetworkweb.org/download/human.zip; "human.source"; https://regnetworkweb.org/download/RegulatoryDirections.zip; "new_kegg.human.reg.direction.txt"), respectively. BIOCARTA pathway information was downloaded from MSigDB (version 2023.1.Hs; https://www.gsea-msigdb.org/gsea/msigdb/download_file.jsp?filePath=/msigdb/release/2023.1.Hs/msigdb_v2023.1.Hs_files_to_download_locally.zip; "c2.cp.biocarta.v2023.1.Hs.entrez.gmt", "c2.cp.biocarta.v2023.1.Hs.symbols.gmt").
The PubChem compound identifiers (CIDs) of drugs from GDSC were retrieved using the PubChem Identifier Exchange Service (https://pubchem.ncbi.nlm.nih.gov/idexchange/idexchange.cgi) and the webchem R package (version 1.2.0). Corresponding SMILES representations were obtained from PubChem (https://pubchem.ncbi.nlm.nih.gov). Drug sensitivity data, along with cell-line and drug annotation information, were sourced from GDSC Release 8.4 (https://www.cancerrxgene.org/downloads/drug_data?screening_set=GDSC1; https://www.cancerrxgene.org/downloads/drug_data?screening_set=GDSC2; https://www.cancerrxgene.org/celllines; https://www.cancerrxgene.org/compounds). Drug sensitivity data from ChEMBL Release 33 were downloaded using the ChEMBL web resource client Python package (version 0.10.8).
TCGA clinical and TPM data (version 37.0), along with standardized drug names, were obtained using the TCGABiolinks R package (version 2.25.3) and GDISC (https://gdisc.bme.gatech.edu/Data/DrugCorrection.csv; https://gdisc.bme.gatech.edu/Data/GoodlookingTable.csv), respectively. SCLC clinical data and TPM data were collected from Liu, Qian, et al.36 via their supplementary tables (Table S1 and S6A), respectively. All raw data were publicly available, and preprocessing raw data was specified and reproducible according to the Github repository (https://github.com/MinhoLee-DGU/GCNPath2026; "IC50_Prediction/project" subdirectory). Source data are provided with this paper. Source data with large volumes were separately uploaded to the same Github repository ("_Supplementary_Data" subdirectory).

Research involving human participants, their data, or biological material

Policy information about studies with human participants or human data. See also policy information about sex, gender (identity/presentation), and sexual orientation and race, ethnicity and racism.

Reporting on sex and gender	N/A
Reporting on race, ethnicity, or other socially relevant groupings	N/A
Population characteristics	N/A
Recruitment	N/A
Ethics oversight	N/A

Note that full information on the approval of the study protocol must also be provided in the manuscript.

Field-specific reporting

Please select the one below that is the best fit for your research. If you are not sure, read the appropriate sections before making your selection.

☒ Life sciences ☐ Behavioural & social sciences ☐ Ecological, evolutionary & environmental sciences

For a reference copy of the document with all sections, see nature.com/documents/nr-reporting-summary-flat.pdf

Life sciences study design

All studies must disclose on these points even when the disclosure is negative.

Sample size	Sample sizes were chosen based on data availability in public domain. In benchmark tests with GDSC datasets, DRPreter, TGDRP, TGSA, and PaccMann can train or test on fewer drug sensitivity data than out model due to the smaller number of samples in the cell-line data they utilize. Therefore, we separately trained their model with (1) their original cell-line datasets and (2) the SANGER Passport TPM data that our model utilizes.
Data exclusions	In the external tests with ChEMBL dataset, we filtered out the following drug response data: those with missing response values, those for

Data exclusions	which cell-line omics data in any model or drug structures were unavailable, those for drugs already screened in GDSC datasets, and those through high-throughput screening methods, such as those from NCI-60. For predictions of TCGA dataset, we filtered out the following drug response data: those with missing or multiple response values, those for which drug structures or patient TPM data were unavailable, those for which the number of responses in each drug was less than ten.
Replication	We did not conduct any replication experiments. All the data were sourced from the public domain.
Randomization	In benchmark tests with GDSC datasets, we conducted 25- and 10-fold outer cross validations, dividing the data into cell-and-drug-blind (strict-blind) splits and the other modes (cell-and-drug-stratified, cell-blind, drug-blind ones), respectively. In blind tests, cell-line and/or drug data were randomly assigned to training, validation, and testing datasets without any overlap.
Blinding	In benchmark tests with GDSC datasets, we conducted several cell-and/or-drug-blind tests as mentioned earlier. In external tests with ChEMBL dataset, we excluded response data screened with drugs from GDSC, which are equivalent to the drug-blind tests as mentioned previously.

Reporting for specific materials, systems and methods

We require information from authors about some types of materials, experimental systems and methods used in many studies. Here, indicate whether each material, system or method listed is relevant to your study. If you are not sure if a list item applies to your research, read the appropriate section before selecting a response.

Materials & experimental systems

n/a	Involved in the study
☒	☐ Antibodies
☒	☐ Eukaryotic cell lines
☒	☐ Palaeontology and archaeology
☒	☐ Animals and other organisms
☒	☐ Clinical data
☒	☐ Dual use research of concern
☒	☐ Plants

Methods

n/a	Involved in the study
☒	☐ ChIP-seq
☒	☐ Flow cytometry
☒	☐ MRI-based neuroimaging

Plants

Seed stocks	N/A
Novel plant genotypes	N/A
Authentication	N/A
